# Exchangeable HaloTag Ligands for Super-Resolution Fluorescence Microscopy

**DOI:** 10.1021/jacs.2c11969

**Published:** 2023-01-30

**Authors:** Julian Kompa, Jorick Bruins, Marius Glogger, Jonas Wilhelm, Michelle S. Frei, Miroslaw Tarnawski, Elisa D’Este, Mike Heilemann, Julien Hiblot, Kai Johnsson

**Affiliations:** † Department of Chemical Biology, Max Planck Institute for Medical Research, Jahnstrasse 29, Heidelberg 69120, Germany; ‡ Institute of Physical and Theoretical Chemistry, Goethe-University Frankfurt, Max-von-Laue Str. 7, Frankfurt 60438, Germany; § Institute of Chemical Sciences and Engineering (ISIC), École Polytechnique Fédérale de Lausanne (EPFL), Lausanne 1015, Switzerland; ∥ Protein Expression and Characterization Facility, Max Planck Institute for Medical Research, Jahnstrasse 29, Heidelberg 69120, Germany; ⊥ Optical Microscopy Facility, Max Planck Institute for Medical Research, Jahnstrasse 29, Heidelberg 69120, Germany

## Abstract

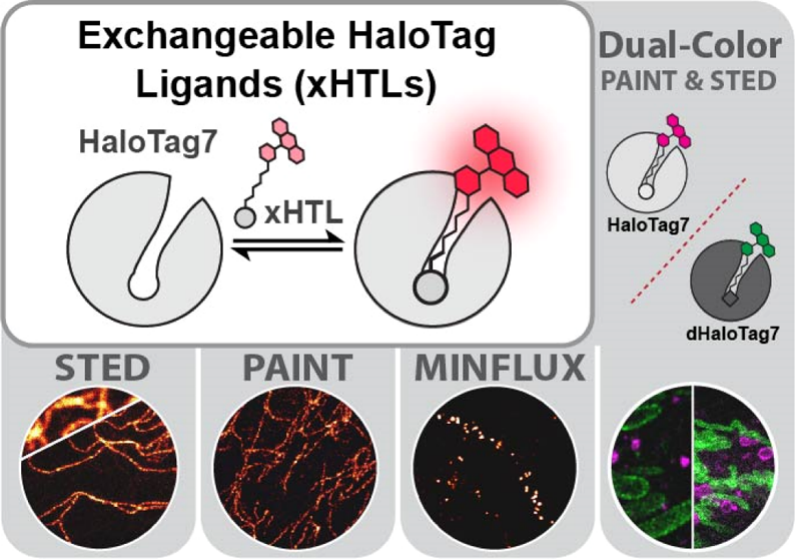

The specific and covalent labeling of the protein HaloTag with fluorescent probes in living cells makes it a powerful tool for bioimaging. However, the irreversible attachment of the probe to HaloTag precludes imaging applications that require transient binding of the probe and comes with the risk of irreversible photobleaching. Here, we introduce exchangeable ligands for fluorescence labeling of HaloTag (xHTLs) that reversibly bind to HaloTag and that can be coupled to rhodamines of different colors. In stimulated emission depletion (STED) microscopy, probe exchange of xHTLs allows imaging with reduced photobleaching as compared to covalent HaloTag labeling. Transient binding of fluorogenic xHTLs to HaloTag fusion proteins enables points accumulation for imaging in nanoscale topography (PAINT) and MINFLUX microscopy. We furthermore introduce pairs of xHTLs and HaloTag mutants for dual-color PAINT and STED microscopy. xHTLs thus open up new possibilities in imaging across microscopy platforms for a widely used labeling approach.

## Introduction

**Figure 1:**
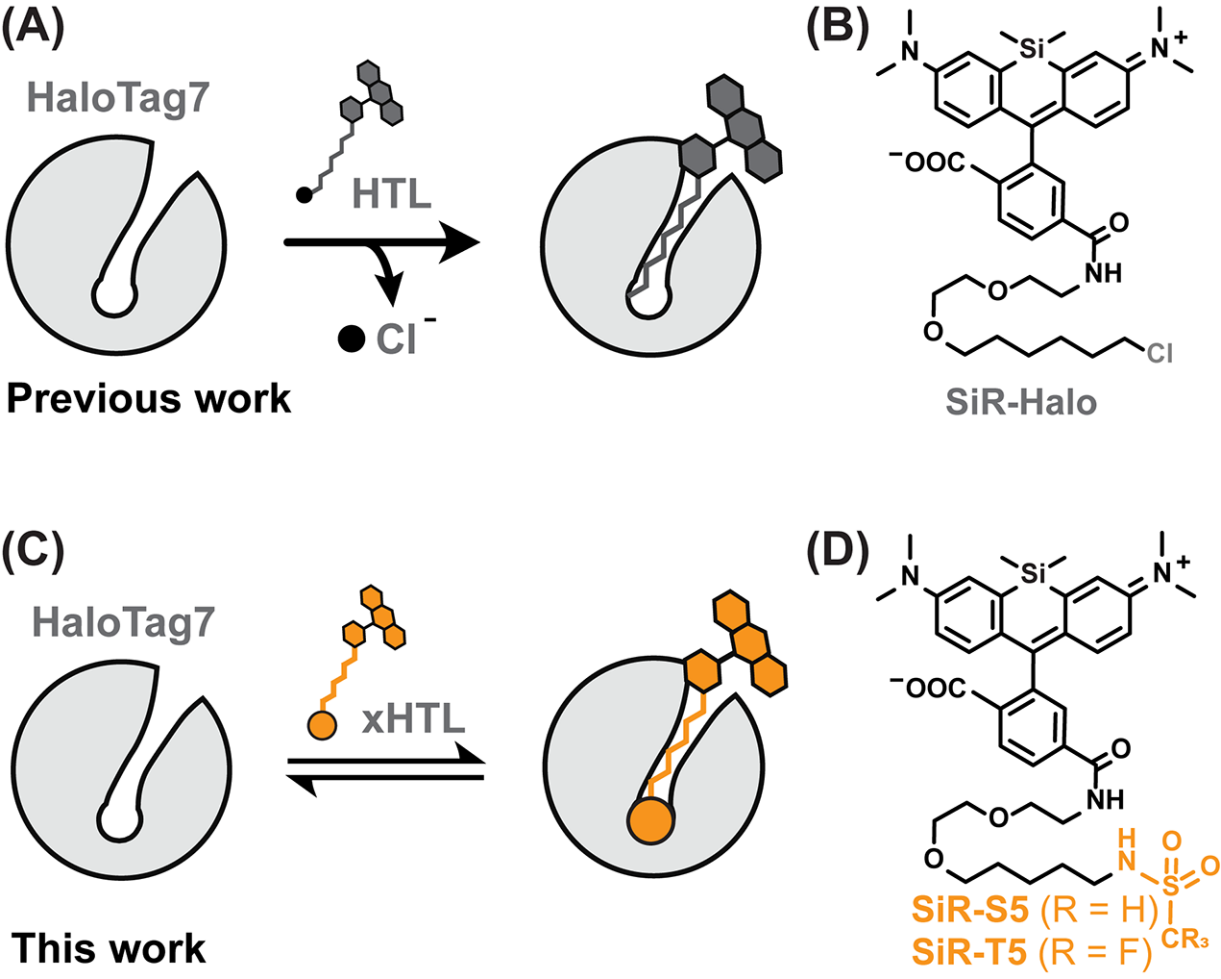
(A) Scheme of covalent HaloTag7 labeling with a fluorescent ligand. (B) Structure of silicon rhodamine (SiR) modified with chloroalkane (SiR-CA = SiR-Halo^35^). (C) Scheme of non-covalent HaloTag7 labeling with a fluorescent exchangeable HaloTag Ligand (*x*HTL). (D) Structure of SiR-*x*HTLs consisting of methylsulfonamide (S5) or trifluoromethylsulfonamide (T5) ligands attached to the rhodamine.

Technical advances in super-resolution microscopy (SRM) have revolutionized our understanding in cell biology.^1^ However, to fully exploit these advances the challenge of specifically labeling biomolecules with suitable fluorophores has to be overcome. A popular approach for labeling proteins with synthetic fluorophores in living cells is based on self-labeling protein (SLP) tags such as SNAP-tag,^2^ CLIP-tag,^3^ or HaloTag7^4,5^ as these proteins specifically and covalently react with synthetic fluorescent probes. The combination of HaloTag7 with rhodamine-based dyes is particularly attractive for bioimaging as HaloTag7 reacts rapidly with chloroalkane (CA) HaloTag ligands (HTLs) fused to rhodamines^6^ (Figure 1A,B). Rhodamines are available in different colors^7^ with high brightness and good photostability, required to resist the photobleaching induced by, for example, stimulated emission depletion (STED)^8^ microscopy.^9^ Furthermore, rhodamine derivatives can be engineered to possess fluorogenicity and cell permeability due to the dynamic equilibrium of rhodamines between a fluorescent zwitterion (i.e., open form) and a nonfluorescent spirocyclic form (i.e., closed form).^10^ The nonfluorescent and apolar spirocyclic form increases the membrane permeability of the probes, while binding to HaloTag7 shifts the equilibrium back to the fluorescent zwitterion, resulting in fluorogenicity. Rhodamine spirocyclization can also be tuned to generate self-blinking probes for single-molecule localization microscopy (SMLM).^11^

Despite the utility of the covalent labeling of HaloTag7 with rhodamine-based probes in SRM,^12,13^ the irreversible nature of the labeling can become a limitation. For example, photobleaching of fluorescent probes still poses a problem in multiframe acquisition in STED microscopy.^14^ Exchangeable fluorescent probes, that are constantly replenished from a large buffer reservoir, provide an elegant way to reduce such photobleaching.^15^ For example, non-covalent labeling approaches based on weak-affinity DNA hybridization show reduced photobleaching in STED microscopy due to continuous probe exchange.^14^ However, a bleached HaloTag7-bound rhodamine cannot be exchanged.

Non-covalent binding/unbinding of fluorescent probes to a target is also the basis for a number of single-molecule-based SRM methods. For example, transient and repetitive DNA hybridization of a fluorescently labeled imager strand can be exploited to reach nanometer resolution in DNA-points accumulation for imaging in nanoscale topography (DNA-PAINT)^16^ and DNA-PAINT MINFLUX^17^ microscopy. While DNA-PAINT is driven by an easy implementation and low technical requirements,^18^ this approach remains exclusively applicable to fixed samples and often entails the use of antibody conjugation, resulting in an inherent loss in resolution.^19^ The specific and covalent labeling of HaloTag7 with fluorescent probes however is incompatible with such an approach. Protein- or peptide-based tags that reversibly bind to fluorescent probes have been developed for applications in SRM microscopy.^20−22^ Fluorogen-binding proteins are proteins that bind non-covalently to fluorophores as shown for UnaG (binding to bilirubin),^21^ IRFPs (binding to biliverdin^23,24^), Fluorogen-activating proteins (FAPs binding to malachite green,^25^ BODIPY dyes,^26^ or GFP-like chromophores^22,27^), or FAST (binding to 4-hydroxybenzylidene-rhodanine and derivatives^28−30^). However, these fluorescent probes are inferior in terms of brightness and photostability to rhodamines used in conjunction with HaloTag7. In live-cell STED microscopy, for example, rhodamine-labeled HaloTag7 still allows to image more consecutive frames than FAST-tag despite not being able to exchange bleached dyes.^30^

Here, we introduce exchangeable HaloTag Ligands (xHTLs) for the reversible fluorescence labeling of HaloTag7 with rhodamines (Figure 1C), opening up new possibilities in bioimaging for a powerful labeling tool. The rapid ligand exchange of xHTLs enables PAINT and MINFLUX microscopy. In live-cell STED microscopy, probe exchange of xHTLs allows extended multiframe imaging. Introducing xHTL/HaloTag pairs with different substrate specificities furthermore enables PAINT and STED microscopy in dual color.

## Results and Discussion

### xHTL Development and Characterization

Taking advantage of the crystal structure of HaloTag7 labeled with tetramethylrhodamine (TMR), we used computational screening to identify tentative xHTLs (Figure S1). In brief, putative xHTLs were designed by varying the terminal alkane chain length (C_4_–C_7_) as well as by replacing the chlorine atom with other functional groups (Figure S1A). After docking of 2000 molecules into the HaloTag7 crystal structure (PDB ID: 6Y7A, Figure S1B), the binding energies (Δ*G*_bind_) of each of these were predicted (Figure S1C). TMR-linked PEG_2_-C_5_-methylsulfonamide (S5) was one of the compounds that presented a lower Δ*G*_bind_ compared to regular TMR-CA (ΔΔ*G*_bind_ = −4.2 kcal/mol, Figure S1C), which displays a *K*_D_ < 1 μM.^6^ We synthesized S5 as well as a fluorinated triflamide derivative T5 in order to improve cell permeability^31^ and coupled both to fluorogenic rhodamine dyes^10,32−34^ such as SiR^35^ (Figure 1D).

**Figure 2:**
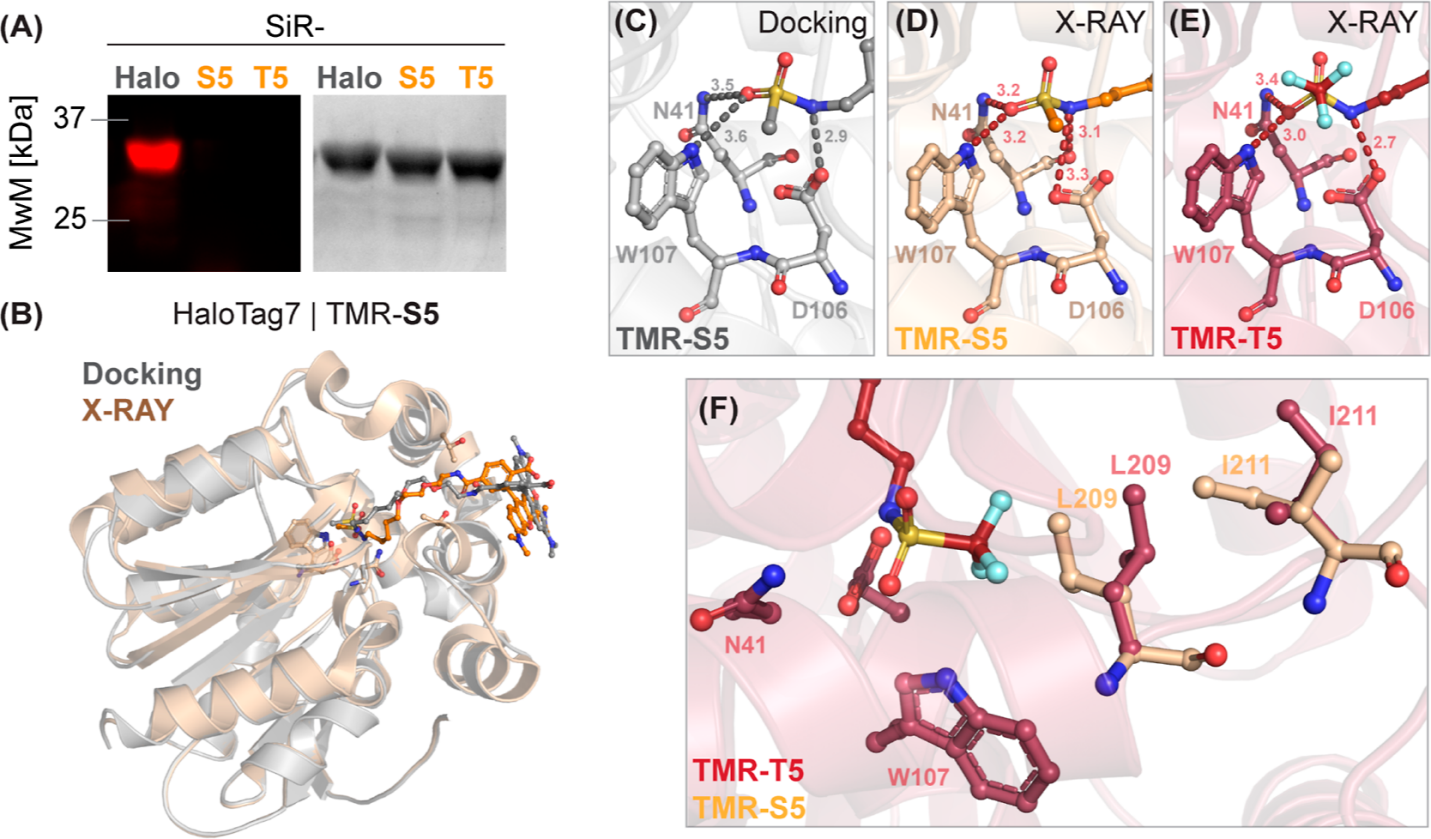
(A) HaloTag7 covalent labeling experiments. SDS-PAGE followed by in-gel fluorescence scanning and Coomassie-staining after incubating SiR-(*x*)HTL probes with HaloTag7. MwM—Molecular weight marker. (B) Structural comparison of the TMR-S5/HaloTag7 complex docking and X-ray structure (PDB-ID: 7ZJ0, 1.5 Å resolution). (C) Magnification on the binding pocket of the TMR-S5/HaloTag7 complex docking. Binding energy was reduced compared to redocking for TMR-CA (ΔΔ*G*_bind_ = −4.2 kcal/mol). (D) Binding pockets of the TMR-S5/HaloTag7 and (E) TMR-T5/HaloTag7 (PDB-ID: 7ZJY, 1.5 Å resolution) complex crystal structures. Polar interactions are highlighted with dashed lines. Distances in Å. (F) Structural comparison between the binding pockets of the TMR-S5 and TMR-T5/HaloTag7 complex crystal structures.

Both ligands show no covalent labeling (Figure 2A), and the *K*_D_ values of HaloTag7 for SiR-S5 and SiR-T5 were determined to be in the sub-micromolar range (Table 1). The measured k_on_ for these derivatives was above 10^6^ M^–1^ s^–1^ (Table 1). Consequently, the *k*_off_ for the bound probe can be estimated to be around 1 s^–1^. High *k*_off_ values are favorable in PAINT microscopy as fast exchange rates reduce the overall imaging duration.^36^ Also, fast exchange rates are essential to ensure efficient probe exchange to overcome photobleaching.^37^

**Table 1:** Binding Properties of Rhodamine-xHTLs to HaloTag7

HaloTag7/ligand		*K*_D_^a^ [nM]		*k*_on_^b^ [10^6^M^–1^s^–1^]		*k*_off_^c^[s^–1^]	
TMR	S5	311	(275–351)	6.0	(5.8–6.1)	1.9	(1.6–2.2)
CPY	S5	142	(115–176)	2.0	(1.9–2.0)	0.3	(0.2–0.4)
SiR	S5	109	(91–130)	4.0	(4.4–4.9)	0.4	(0.2–0.6)
TMR	T5	166	(150–185)	5.6	(5.5–5.8)	0.7	(0.5–1.1)
CPY	T5	60	(48–75)	4.4	(4.3–4.5)	0.3	(0.2–0.4)
SiR	T5	67	(48–93)	5.5	(5.1–5.8)	0.4	(0.2–0.5)

a *K*_D_ obtained from fluorescence polarization assay.

b *k*_on_ obtained from stopped-flow measurement.

c *k*_off_ was calculated from *K*_D_ and *k*_on_. Errors provided as 95% confidence intervals.

The binding mode of TMR-S5 and TMR-T5 to HaloTag7 was further studied through X-ray crystallography. First, the crystal structure of HaloTag7 in complex with TMR-S5 (PDB ID: 7ZJ0, 1.5 Å resolution, Figure S2, Table S2) was compared to the computed docking pose of TMR-S5 in HaloTag7 (Figure 2B). While the S5 moiety occupies a similar position in both the docking experiment and the crystal structure (Figure 2C,D, rmsd = 1.0 ± 0.3 Å on five atoms), the TMR shows a different position on the HaloTag7 surface in the docking experiment (Figure S3A,B). The alkane-PEG chain occupies the same hydrophobic channel but presents a different conformation (Figure S3C). The crystal structures of HaloTag7 in complex with TMR-S5 and TMR-T5 (PDB ID: 7ZJY, 1.5 Å resolution, Figure S4, Table S2) revealed that the sulfonamides occupy the active site of HaloTag7, while TMR is bound as in the covalently labeled HaloTag7 (PDB ID: 6Y7A, Figures S2 and S4). The main difference resides in a reorientation of the residues L209 and I211 in order to accommodate the larger trifluoromethyl group in the active site (Figure 2F). The L209/I211 conformational change induced by the TMR-T5 binding in the HaloTag7 active site allows a more favorable orientation of the sulfonamide group to form hydrogen bonds with active side residues than observed for TMR-S5 (Figure 2D,E); while the TMR-S5 nitrogen-sulfonamide forms a 3.3 Å hydrogen bond with the catalytic D106 carboxylic acid moiety, the corresponding hydrogen bond in bound TMR-T5 is only 2.7 Å. This difference in binding interactions might contribute to the higher affinity of T5-based probes than those of S5-based probes.

**Figure 3:**
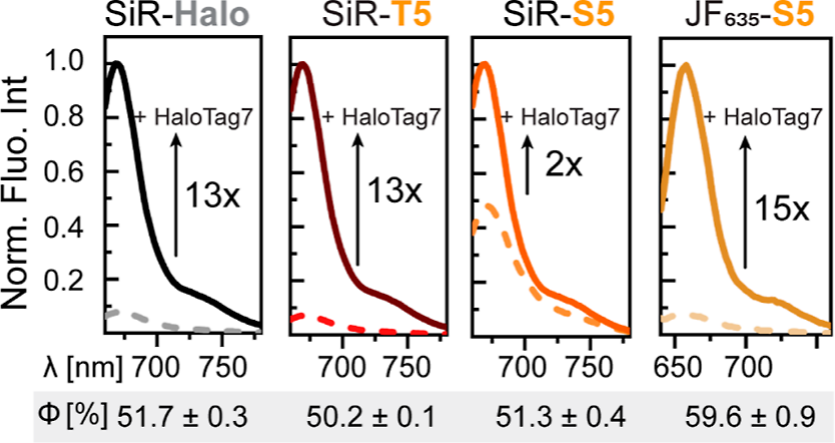
Fluorescence emission spectra of free (dashed lines) and HaloTag7-bound (plain line) SiR- or JF_635_-(*x*)HTLs. Quantum yields (Φ) in the presence of HaloTag7 are indicated below.

Application of non-covalent probes for live-cell microscopy requires to perform imaging in the presence of the dye (“no-wash”), and the availability of fluorogenic dyes would facilitate such experiments. Covalent binding of rhodamine-based probes to HaloTag7 shifts the equilibrium of spirocyclization toward the fluorescent zwitterion, resulting in fluorescence increase.^33^ To investigate if *x*HTLs show a similar behavior, we measured the fluorescence of SiR-T5, SiR-S5, and SiR-Halo in the presence (*F*) and the absence (*F*_0_) of an excess of HaloTag7. *F*/*F*_0_ measured for SiR-T5 (12.6 ± 4.0) upon HaloTag7 binding was comparable to the value measured for covalently bound SiR-Halo (Figure 3). In contrast, SiR- S5 showed only a 2-fold fluorescence increase upon HaloTag7 binding (Figure 3). The lower fluorogenicity of SiR-S5 results from its lower propensity to exist in the nonfluorescent, spirocyclic form than SiR-T5 and SiR-Halo (Figure S5A). It was previously shown that the equilibrium between the fluorescent zwitterion and the nonfluorescent spirocyclic form of rhodamine-based probes depends on the polarity of the ligand attached to the rhodamine^38^ and that *D*_50_ values between 50 and 75 are best suited to develop fluorogenic probes.^33^ When fluorophores such as JF_635_, which show a higher propensity to form the spirocyclic form than SiR,^10^ are coupled to S5, the *F*/*F*_0_ values obtained for JF_635_-S5 are comparable to those observed for SiR-Halo (Figure 2E). Among the different rhodamine-xHTL derivatives, MaP618-S5 (*F*/*F*_0_ = 29.9 ± 5.2) and JF_585_-T5 (*F*/*F*_0_ = 67.7 ± 6.7) showed the highest fluorogenicity (Figure S5B, Table S3). Furthermore, rhodamine-xHTLs show similar quantum yields (Φ) when bound to HaloTag7 as their covalently bound counterparts (Figure 3, Table S1).^39,40^ The affinity of xHTLs toward HaloTag7 is largely independent of the nature of the attached rhodamine (Table S1), yielding access to a wide color-range of *x*HTL-based probes.

### *x*HTL Live-Cell Imaging

**Figure 4:**
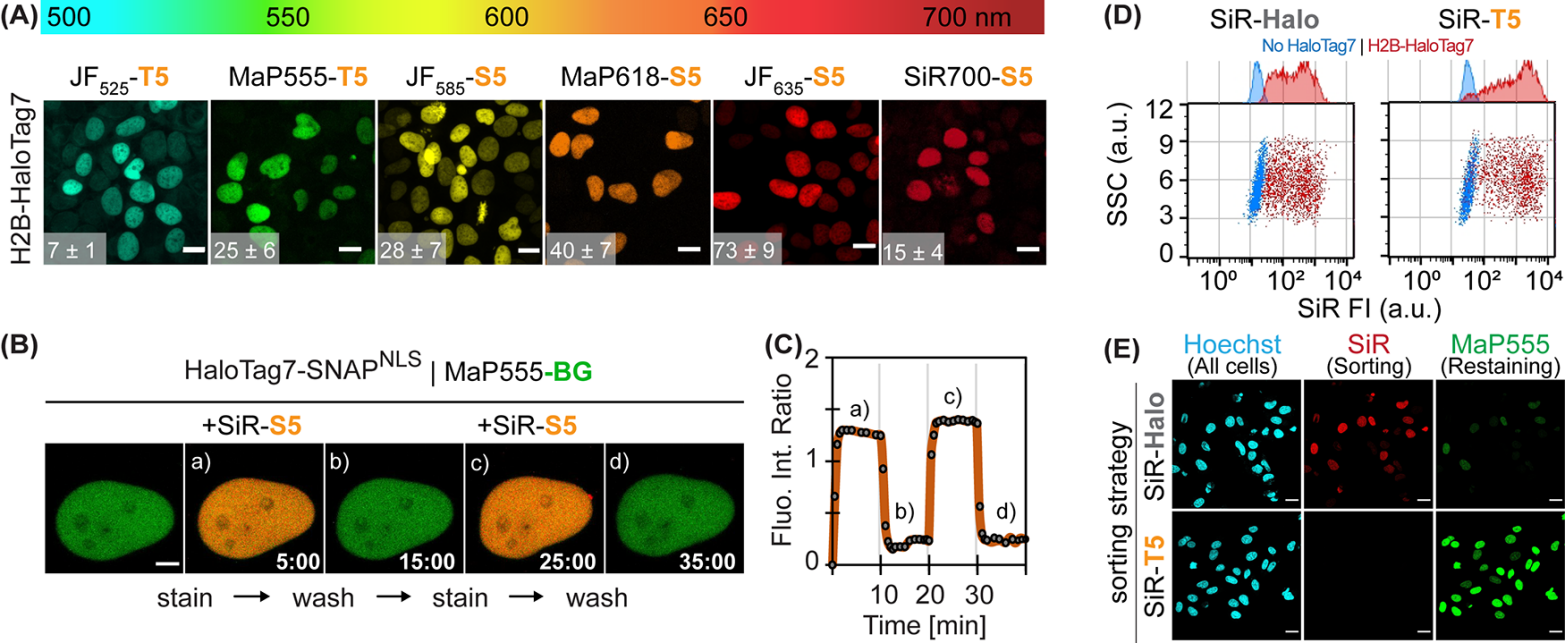
(A) Live-cell confocal images of different fluorescent *x*HTL probes covering the visible spectrum. Histone2B-HaloTag7 (H2B-HaloTag7) expressing U2OS cells stained with 500 nM *x*HTLs. Sum projections. Scale bars: 10 μm. Signal-over-background ratios (S/B) are indicated in the bottom left corner (*n* ≥ 50 cells, mean ± standard error of the mean). (B) Reversible cellular staining using *x*HTLs. Live-cell confocal images of U2OS expressing HaloTag7-SNAP^NLS^ (nuclear localization signal) covalently labeled with MaP555-BG and iteratively stained with 500 nM SiR-S5 or washed with an imaging medium (10 min cycles). Sum projections. Scale bar: 2 μm. Images recorded every 30 s. (C) Intensity-time-trace given of the experiments shown in B. Fluorescence intensity ratio is FI_SiR_/FI_MaP555_. (D) Flow cytometry profiles of cells stained with SiR-Halo or SiR-T5 (500 nM). U2OS expressing no HaloTag7 or Histone2B-HaloTag7 fusions. Sideward scatter (SSC) vs. SiR fluorescence intensity (SiR FI) are presented. (E) Confocal microscopy images of Histone2B-HaloTag7 16 h after cell sorting with SiR-Halo or -T5 (500 nM). Restaining using Hoechst (1 μg/mL) and MaP555-CA (500 nM).

We then evaluated the ability of the different rhodamine derivatives of S5 and T5 to stain live U2OS cells expressing Histone2B-HaloTag7. xHTL probes specifically stained the nucleus with signal-over-background (S/B) ranging from 7.2 ± 0.8 (JF_525_-T5) up to 73.1 ± 8.4 (JF_635_-S5, Figure 4A), except TMR-S5 and -T5, which were not able to stain live U2OS cells but allowed staining in fixed cells (Figure S6). For the far-red dyes, both S5 and T5 derivatives showed specific labeling. However, only T5 allowed specific labeling with MaP555 and JF_525_ in live cells. Specific labeling was also observed for HaloTag7 fusion proteins at other subcellular localizations (Figure S7A) as well as for other cell types (Figure S7B) including primary cells (cultured rat hippocampal neurons, Figure S7C). Higher cellular brightness was achieved with covalent labeling of HaloTag7 fusion proteins (Figure S8). Indeed, while covalent labeling should go to completion, a fraction of the HaloTag7 fusion protein remains unbound with exchangeable S5- and T5-based probes. Exchange of *x*HTL binding to HaloTag7 in cells was demonstrated by repetitive labeling and washing with SiR-S5 of U2OS cells expressing nuclear localized HaloTag7 (HaloTag7^NLS^), where labeling and wash-out was achieved within ∼1 min (Figure 4B,C, Video S1). SiR-T5 showed even faster labeling kinetics of Histone2B-HaloTag7 expressed in live U2OS cells than SiR-Halo (Figure S9). *x*HTLs such as SiR-T5 are also suited for fluorescence-activated cell-sorting (FACS, Figure 4D) of U2OS Histone2B-HaloTag7 cells, allowing to relabel sorted cells with another probe for subsequent experiments (Figure 4E).

### PAINT, MINFLUX, and STED Microscopy with *x*HTLs

**Figure 5:**
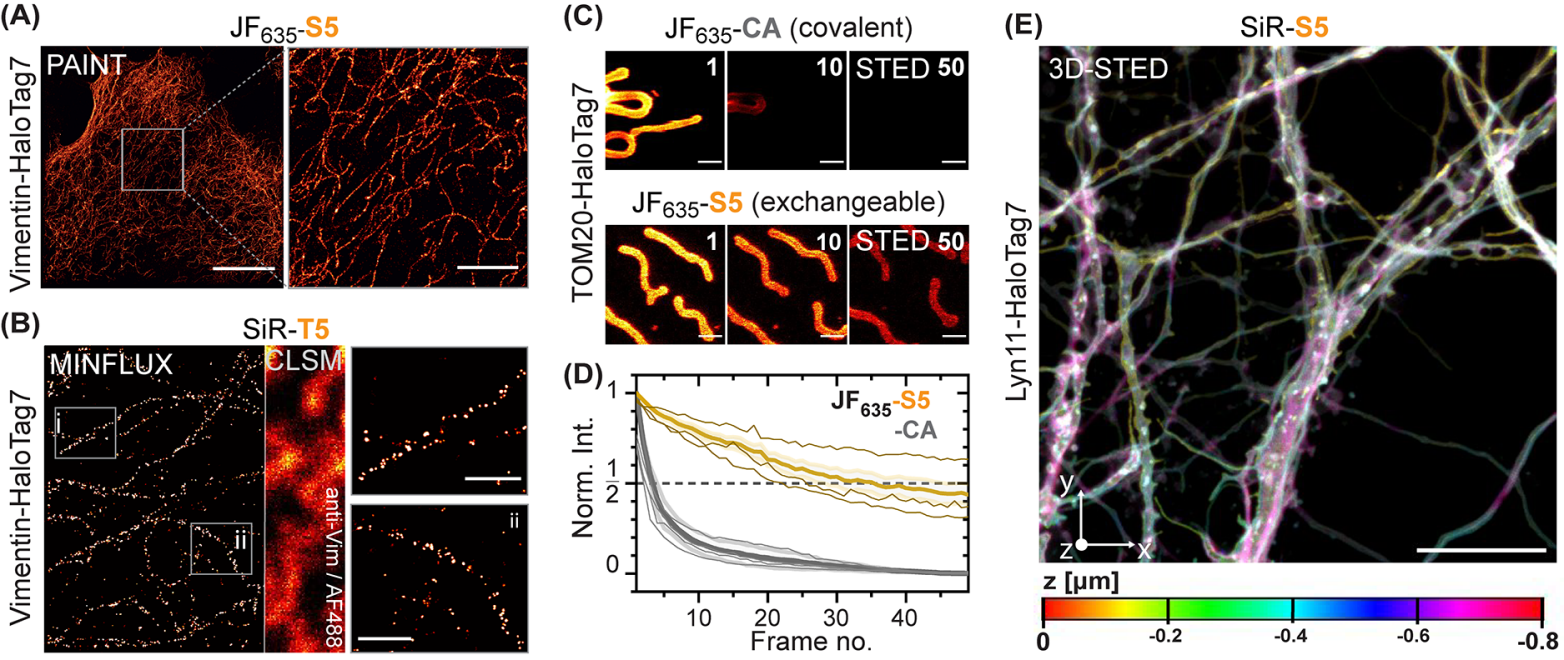
(A) HaloTag-PAINT image of a fixed U2OS cell endogenously expressing vimentin-HaloTag7^41^ labeled with JF_635_-S5 (5 nM). Scale bar: 10 μm (overview) and 2 μm (magnified region). (B) 2D-MINFLUX microscopy image of fixed U2OS cells expressing vimentin-HaloTag7 labeled with SiR-T5 (2 nM). Vimentin immunostaining (AF488) and confocal laser-scanning microscopy (CLSM) imaging was used as a reference. Magnification reveals vimentin-HaloTag7 molecules with a localization precision of ∼3.9 nm. Intensities are represented in arbitrary units from 0 to 3 (overview) or 0 to 12 (magnified region). Scale bars: 0.2 μm. (C) Multiframe STED images of U2OS mitochondria outer membrane (TOM20-HaloTag7) labeled with JF_635_-CA or JF_635_-S5. Frame numbers indicated in the top right corner. Scale bar: 1 μm. (D) Bleaching curves (thick lines: mean value and S.D., thin lines: individual experiments) from similar experiment as shown in C. (E) 3D-STED image of Lyn11-HaloTag7 (plasma membrane) from live cultured rat hippocampal neurons labeled with SiR-S5. An area of 40 × 40 μm (*x*–*y*, 80 nm pixel-size) was recorded in 20 nm z-stacks over 40 times (0.8 μm z-depth). Max. projection and depth color-coding. Scale bar: 10 μm.

In vitro characterization and cellular imaging experiments suggest that *x*HTLs possess specificity, affinity, and exchange rates that make them suitable for PAINT. Furthermore, the fluorogenicity of some xHTLs should reduce the background signal. We therefore investigated the performance of SiR-S5, SiR-T5, and JF_635_-S5 in PAINT experiments using fixed U2OS cells expressing vimentin-HaloTag7 fusions.^41^ All three xHTLs tested allowed to resolve single binding events (Video S2) such that PAINT images of intermediate filaments (vimentin-HaloTag7 fusions) in fixed U2OS cells could be reconstructed (Figure 5A). The spatial resolution was calculated using decorrelation analysis^42^ and was comparable to that achieved with DNA-PAINT^36^ probes using oligonucleotides coupled to antibodies yielding ∼32 nm in both cases (Figure S10A). Furthermore, we performed Fourier ring correlation (FRC) analysis,^43^ which reported a spatial resolution of 36 ± 8 nm and calculated the experimental localization precision (based on the nearest neighbor approach^44^) to 11 nm. These experiments also allowed to determine the bright times τ_B_ (Figure S10B), which corresponds to the average time period a fluorophore spends bound to HaloTag7 in its zwitterionic, open form. τ_B_ for the three tested *x*HTLs lay in the range of 0.37–0.71 s, corresponding to off-rates (*k*_off_) in the range of 1.4—2.7 s^–1^. JF_635_-S5 turned out to be particularly suited for PAINT microscopy by combining high off-rates (2.7 s^–1^) and reduced background, ultimately yielding the best resolution.

We then investigated if the transient staining could be compatible with MINFLUX imaging,^45^ a microscopy method that has been demonstrated to routinely reach single-digit nm resolution. Recently, DNA-PAINT has been shown to be compatible with MINFLUX imaging and thus xHTLs in principle could find applications in MINFLUX as well.^17^ Using SiR-T5 and fixed U2OS cells expressing vimentin-HaloTag7, single molecules could be imaged in 2D MINFLUX with ∼ 4.0 nm localization precision by using 220 photons in the last iteration step (*L* = 40 nm) (Figures 5C and S11). Thus, *x*HTLs can be used for the imaging of HaloTag7 fusion proteins using MINFLUX.

STED imaging requires high laser intensities that increase the risk of fluorophore bleaching and thereby reduce the capacity of this approach to study dynamic processes over a high number of frames. Exchangeable fluorophores can circumvent this issue by replacing bleached fluorophores with intact ones.^14^ In live-cell STED microscopy, SiR-T5 non-covalent labeling of vimentin-HaloTag7^41^ of U2OS allowed to acquire images in which intermediate filaments had similar diameters (full-width at half maximum, FWHM) as for covalent HaloTag7 labeling (Figure S12A,B). However, xHTL staining was significantly less susceptible to photobleaching compared to covalent labeling. The transient labeling with JF_635_-S5 allowed to trace mitochondria (TOM20-HaloTag7, outer mitochondrial membrane) in live cells over 50 consecutive frames, while covalent labeling with JF_635_-CA limited the imaging to less than 10 frames (Figure 5C,D, Video S3). A panel of fluorogenic xHTLs (MaP555-T5, MaP618-S5, JF_635_-S5, SiR-S5, and SiR-T5) was characterized in multiframe STED imaging of mitochondria in live U2OS cells. Overall, the STED-frame numbers with >50% signal intensity (τ_1/2_) was increased by 3- to 5-fold compared to covalent labeling for all fluorophores tested (Figure S12C–H). Using SiR-T5, it was possible to image with a maximum frequency of ∼1 frame/second in an 8 μm^2^ area at a high resolution (Video S4). *x*HTLs are compatible with STED imaging of rat hippocampal neurons in 2D (Figure S13) and in 3D [Figure 5E, (Video S5)]. The remaining photobleaching we observed might originate from HaloTag7 damage by either (i) photoinduced cross-linking and bleaching of the probes or (ii) photodamage to HaloTag7 through reactive oxygen species (ROS)^46^ preventing further probe binding or fluorogenic response. Overall, the observed increase in STED frame number acquisition establishes xHTLs as powerful probes for multiframe and volumetric imaging.

### xHTLs for Dual-Color Microscopy

**Figure 6:**
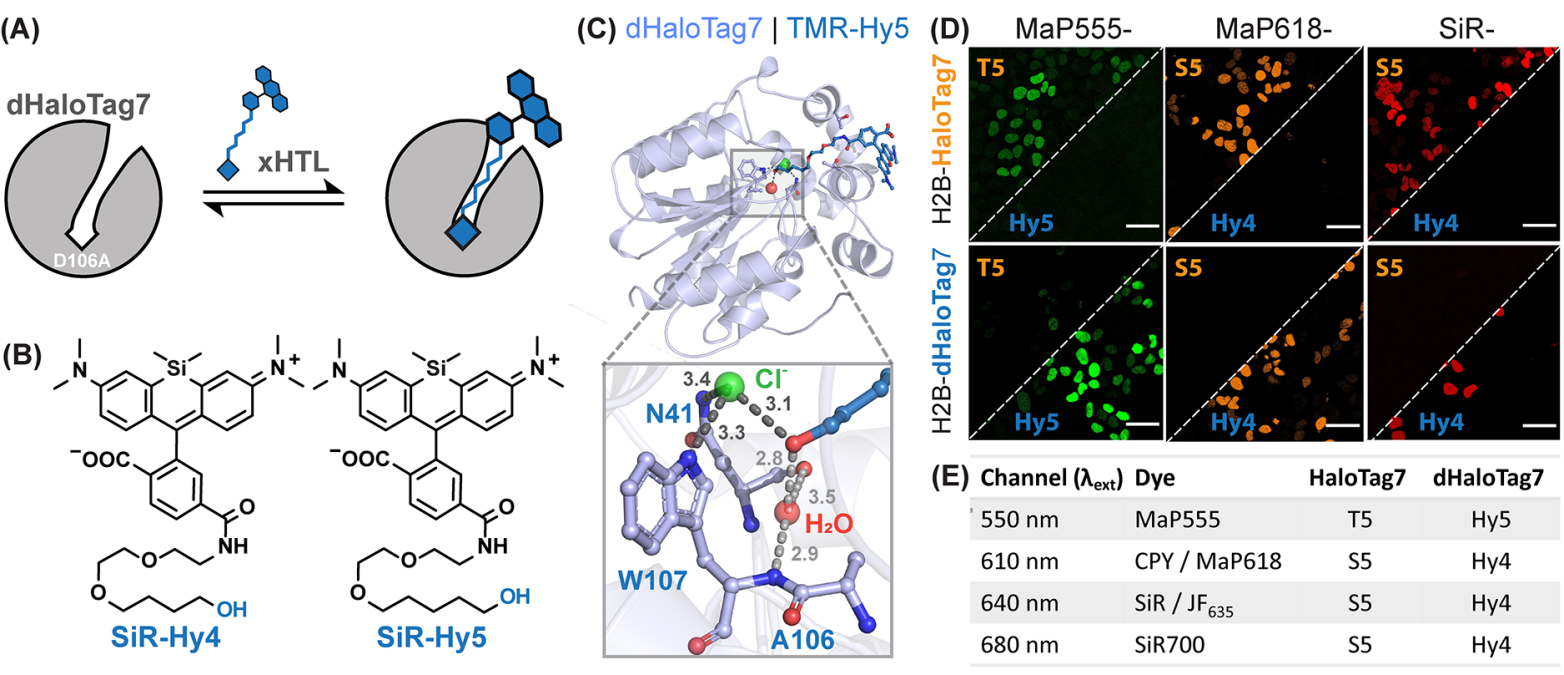
(A) Scheme of non-covalent dHaloTag7 labeling with a fluorescent *x*HTL. (B) Structure of SiR-*x*HTLs consisting of alkane-hydroxy (Hy4 and Hy5) ligands attached to rhodamines. (C) Structural analysis of the TMR-Hy5/dHaloTag7 complex (PDB-ID: 7ZIZ, 1.5 Å resolution). Magnification on the binding pocket (distances in Å). (D) Representative confocal images of live U2OS cells expressing H2B-HaloTag7 or H2B-dHaloTag7 labeled with annotated *x*HTLs. Scale bars: 10 μm. (E) Summarizing table of *x*HTL combinations for two-color live-cell fluorescence microscopy.

We then developed a second reversible labeling system with different ligand specificity, using a HaloTag7 mutant, for dual-color microscopy with xHTLs. Specifically, we mutated the active-site residue Asp106 in HaloTag7 to Ala (D106A), resulting in dHaloTag7^6^ (Figure 6A). The loss of the interaction of the (trifluoromethyl)sulfonamide of *x*HTLs with D106 resulted in up to 57-fold lower affinities of dHaloTag7 for these probes compared to HaloTag7 (Table S1). We then found that primary hydroxy derivatives with different alkyl chain lengths (Hy4 and Hy5; Figure 6B) displayed up to 108-fold higher binding affinity for dHaloTag7 over HaloTag7 (Table S1). Hy4 and Hy5 also possess fast non-covalent binding to dHaloTag7 (Figure S14A,B, Table 2) and, coupled to appropriate fluorophores, fluorogenicity comparable to S5 probes (Figure S14C). For example, MaP618-Hy4 showed a fluorescence intensity increase upon dHaloTag7 binding up to 37.1 ± 6.5-fold (Table S3). Structural analysis of the complex of TMR-Hy5 and dHaloTag7 (Figure 6C and S15, PDB-ID: 7ZIZ, 1.5 Å resolution) revealed the presence of a structural chloride ion and a water molecule in the binding site, which were not present in any of the S5 and T5 structures. While the chloride forms hydrogen bonds with the W107 and N41 main chains, the water molecule occupies the space freed by the D106A mutation and interacts with the W107 and N41 side chains. Both chloride and water molecules form hydrogen bonds with the Hy5 ligand.

**Table 2:** Binding Properties of Rhodamine-xHTLs to dHaloTag7

dHaloTag7/ligand		*K*_D_^a^[nM]		*k*_on_^b^ [10^6^M–1s–1]		*k*_off_^c^[s–1]	
CPY	Hy4	385	(253–589)	9.4	(9.0–9.8)	3.6	(2.3–5.8)
SiR	Hy4	272	(170–436)	13.3	(12.2–14.4)	5.1	(3.1–8.5)
MaP555	Hy5	186	(123–189)	9.3	(8.9–9.7)	2.9	(1.8–3.1)
SiR	Hy5	86	(74–100)	11.2	(10.7–11.7)	1.0	(0.8–1.2)

a *K*_D_ obtained from fluorescence polarization assay.

b *k*_on_ obtained from stopped-flow measurement.

c *k*_off_ was calculated from *K*_D_ and *k*_on_. Errors provided as 95% confidence intervals.

Hy4 and Hy5 *x*HTL can be used for the specific staining of nuclei of live U2OS cells expressing Histone2B-dHaloTag7 (Figure S16A). Hy5-based xHTLs showed higher binding affinity than the corresponding Hy4-based *x*HTLs, which was required in the case of MaP555-Hy5 for selective staining of intracellular dHaloTag7. For red-shifted fluorophores, such as SiR, both Hy5 and Hy4 derivatives showed specific labeling.

We then tested if the affinity differences measured for S5/T5 and Hy4/Hy5 on HaloTag7 and dHaloTag7 are sufficient for dual-color microscopy. In in vitro titrations, probes based on Hy4, Hy5, or S5 had >50-fold higher affinities for their cognate HaloTag variant, whereas T5-based probes showed lower specificity. In live-cell imaging experiments, we identified conditions enabling *x*HTLs to stain their cognate HaloTag7, without staining dHaloTag7 and vice versa (Figures 6D and S16). For dual-color live-cell imaging, we suggest choosing for each color channel the probes displayed in Figure 6E. For applications that require high fluorogenicity, we recommend MaP618 or JF_635_-based probes. For photon-demanding microscopy such as STED microscopy, we recommend the brighter CPY or SiR-probes.

**Figure 7:**
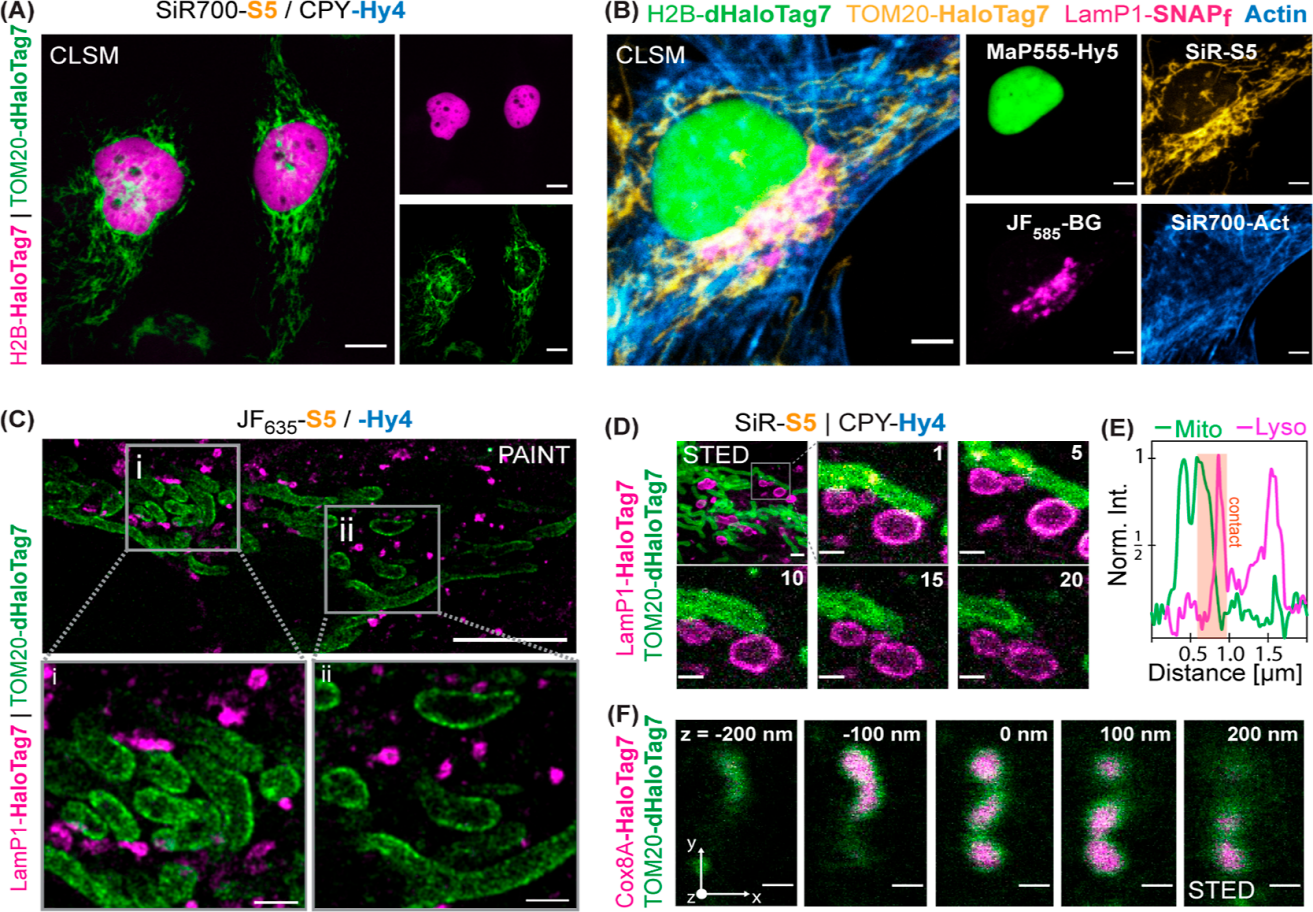
(A) Dual-color live-cell confocal images using combinable *x*HTLs. U2OS cells expressing H2B-HaloTag7 and TOM20-dHaloTag7 labeled with SiR700-S5 and CPY-Hy4 (500 nM). Scale: 10 μm. (B) Four-color confocal image of a U2OS cell live stained using orthogonal *x*HTLs, SNAP-tag. and a SiR700-actin probe (c). MaP555-Hy5, SiR-S5, and JF_585_-BG were used to label H2B-dHaloTag7 (nucleus), TOM20-HaloTag7 (mitochondria surface), and LamP1-SNAP-tag (lysosome), respectively. Scale: 5 μm. (C) Dual-target Exchange-PAINT image of mitochondria and lysosomes of fixed U2OS cells using combinable *x*HTLs. Cells expressing TOM20-HaloTag7 and LamP1-dHaloTag7 via T2A fusion. Sequential labeling and imaging using JF_635_-S5 (5 nM, magenta) and JF_635_–Hy4 (3 nM, green). Scale bars: 10 μm (overview) or 1 μm (magnified region). (D) Dual-color time-lapse STED images of mitochondria-lysosome dynamics in live U2OS cells. Cells labeled with 500 nM *x*HTLs. Imaging over 20 consecutive frames, 2 frames/minute, 10 μm^2^ area. Frame numbers indicated in the top right corner. SiR- and CPY-*x*HTLs were chosen for their higher brightness in STED imaging. Scale bars: 2 μm (overview) or 0.5 μm (magnified region). (E) Line-scan profile across a lysosomal vesicle mitochondria contact site. (F) 3D-STED images of xHTL-stained U2OS mitochondria. Cells express Cox8A-HaloTag7 (inner membrane) and TOM20-dHaloTag7 (outer membrane) via T2A fusion and were labeled with SiR-S5 and CPY-Hy4 (500 nM). An area of 2.44 × 3.20 μm (*x*–*y*) was recorded in 50 nm z-stacks over 40 times; z-plains are indicated in the top right corner. Scale bar: 0.5 μm.

These *x*HTL pairs enabled dual-color co-staining of live U2OS cells expressing both HaloTag variants (Figures 7A and S17). They can be further combined with established labeling strategies, as demonstrated by four-color confocal imaging of live U2OS cells using HaloTag7 labeled with SiR-S5, dHaloTag7 labeled with MaP555-Hy5, SNAP-tag labeled with BG-JF_585_, and SiR700-actin^38^ (Figure 7B).

In PAINT microscopy, JF_635_-Hy4 imaged vimentin-dHaloTag7 expressed in U2OS cells with a similar resolution as obtained with JF_635_-S5 and HaloTag7 (Figure S18A). Dual-target single-molecule imaging was demonstrated by an Exchange-PAINT approach^47^ in fixed U2OS cells by sequentially imaging mitochondria (TOM20-dHaloTag7) using JF_635_-Hy4 and then either lysosomes (LamP1-HaloTag7, Figure 7C) or the endoplasmic reticulum (CalR-HaloTag7-KDEL, Figure S19A) using JF_635_-S5.

In live-cell STED microscopy, MaP555-Hy5, MaP618-Hy4, JF_635_-Hy4, and SiR-Hy4 allowed imaging of mitochondria (TOM20-dHaloTag7) over significantly higher frame numbers than what was possible using covalently labeled HaloTag7 (up to 73 frames with >50% signal intensity for SiR-Hy4, Figure S18B, Video S6). Dual-color, live-cell STED imaging of lysosomes (LamP1-HaloTag7) and mitochondria (TOM20-dHaloTag7) was performed using SiR-S5 and CPY-Hy4 using a single depletion laser (λ_dep_ = 775 nm). In these experiments, it was possible to detect dynamic mitochondria-lysosome contact sites at super-resolution over 20 frames at ∼2 frames/min frame rate (Figures 7D,E, S19C). Finally, the same approach was employed for 3D imaging of mitochondria in live U2OS cells stained at their outer membrane (TOM20-dHaloTag7) and in their matrix (Cox8A-HaloTag7) in a volume of 15.6 μm^3^ (50 nm z-stacks, Figure 7F). In addition to the here demonstrated applications in live cells, HaloTag7 and dHaloTag7 also allow multicolor STED microscopy in fixed samples.^48^

## Conclusions

Covalent HaloTag technology has become one of the most popular labeling tools in fluorescence microscopy.^49^ Here, we introduce fluorescent and exchangeable HaloTag ligands xHTLs that can be used with the current HaloTag system for live-cell fluorescence staining. *x*HTLs are available in different colors using rhodamine fluorophores with photophysical properties ideally suited for various types of super-resolution microscopy. Specifically, xHTLs extend the HaloTag platform toward PAINT and MINFLUX applications and improve multiframe STED microscopy. Furthermore, both PAINT and STED microscopy can be performed in dual color using pairs of *x*HTLs together with HaloTag7 and dHaloTag7. These features make *x*HTLs an important advance for live-cell imaging especially at high resolution.^9^

## Supporting Information Available

Synthetic pathways, extended material and method section, spectroscopic characterization, structural analysis, in vitro tests, confocal and wide-field fluorescence microscopy images of mammalian cells and hippocampal neurons, super-resolution microscopy images, binding and photophysical properties of dyes, list of plasmids and stable cell lines, list of microscopy settings, NMR spectra, and protein sequences (PDF)
Video S.1 Exchangeable HaloTag7 staining with confocal imaging of a live U2OS cell expressing HaloTag7-SNAP-tag^NLS^ and covalent MaP555-BG counterstain and transient (un-)staining with SiR-S5 (500 nM) every 10 min (ratiometric projection). Images were recorded every 30 s (AVI). Video S.2 HaloTag-PAINT imaging with fixed USOS Vimentin-HaloTag7 cells stained using JF_635_-S5 (5 nM) and spatio-temporal separated single-binding events in consecutive images acquired at 10 Hz and HILO mode. Scale bar: 10 μm (AVI). Video S.3 Multiframe STED imaging of live U2OS cells expressing TOM20-HaloTag7 with JF_635_-CA or -S5 (500 nM). Scale: 1 μm (AVI). Video S.4 Multiframe STED imaging at high-resolution and max. frame rate showing live U2OS cells expressing TOM20-HaloTag7 stained with SiR-T5 (500 nM). 200 frames at a frame-rate of ∼1 frame/sec with minimal photobleaching. Scale: 1 μm (AVI). Video S.5 3D-STED of HaloTag7 expressed on the plasma membrane (Lyn11 fusion) of a live rat hippocampal neuron with area of 40 × 40 μm (*x*–*y*, 80 nm pixel-size) recorded in 20 nm z-stacks over 50-times (1 μm z-depth). Depth color-coding. Scale bar: 10 μm. (AVI). Video S.6 Multiframe STED imaging of live U2OS cells expressing either TOM20-HaloTag7 or -dHaloTag with SiR-Halo or -Hy4, respectively (500 nM). Scale: 1 μm (AVI) (ZIP)

## Notes

This work was supported by the Max Planck Society and the Deutsche Forschungsgemeinschaft (DFG, German Research Foundation), TRR 186 and SFB1177; INST 161/778-1 FUGG.

## Abbreviations

CA: chloroalkane
CPY: carbopyronine
FAST: fluorescence-activating and absorption-shifting tag
FAP: fluorescence-activating protein
FWHM: full-with at half maximum
GFP: green fluorescent protein
HTL: HaloTag Ligand
IRFP: infra-red fluorescent protein
JF: Janelia Fluor dyes
MaP: Max-Planck dyes
PAINT: point-accumulation in nanoscale topography
PEG: polyethylene glycol
rmsd: root-mean-square deviation
SDS-PAGE: sodium dodecyl sulfate-polyacrylamide gel electrophoresis
SiR: silicon rhodamine
SMLM: single-molecule localization microscopy
SRM: super-resolution-microscopy
SSC: sideward scatter
STED: stimulated emission-depletion
TMR: tetramethyl rhodamine
xHTL: exchangeable HaloTag Ligand
