# Supplementary material for: Exchangeable HaloTag Ligands for Super-Resolution Fluorescence Microscopy
Source: J Am Chem Soc. 2023 Jan 30;145(5):3075–83. doi: 10.1021/jacs.2c11969 (PMC9912333; doi:10.1021/jacs.2c11969)
Supplement: Supplementary file 1 — ja2c11969_si_001.pdf [file ja2c11969_si_001.pdf]

Supplementary Information for

Exchangeable HaloTag Ligands for Super-Resolution Fluorescence microscopy

Julian Kompa[†], Jorick Bruins[†], Marius Glogger[‡], Jonas Wilhelm[†], Michelle S. Frei[†,§,#], Miroslaw Tarnawski[‖], Elisa D'Este[⊥], Mike Heilemann[‡], Julien Hiblot[†,*], Kai Johnsson[†,§*]

[†] Department of Chemical Biology, Max Planck Institute for Medical Research, Jahnstrasse 29, 69120 Heidelberg, Germany

[‡] Institute of Physical and Theoretical Chemistry, Goethe-University Frankfurt, Max-von-Laue Str. 7, 60438 Frankfurt, Germany

[§] Institute of Chemical Sciences and Engineering (ISIC), École Polytechnique Fédérale de Lausanne (EPFL), 1015 Lausanne, Switzerland

[‖] Protein Expression and Characterization Facility, Max Planck Institute for Medical Research, Jahnstrasse 29, 69120 Heidelberg, Germany

[⊥] Optical Microscopy Facility, Max Planck Institute for Medical Research, Jahnstrasse 29, 69120 Heidelberg, Germany

Present Address:

[#] Department of Pharmacology, University of California, 92161 San Diego, USA.

* Email: julien.hiblot@mr.mpg.de

* Email: johnsson@mr.mpg.de

Table of content

SUPPLEMENTARY METHODS 4

Molecular docking 4
Chemical Procedures 4
S5 / T5-xHTL 5
Hy4 / Hy5-xHTL 6
Deprotection 7
Fluorophore coupling 7
Molecular biology & Biochemistry 9
Plasmids 9
Protein production and purification 9
Covalent labeling of HaloTag7 protein and in-gel fluorescence scanning 9
Protein crystallization and X-ray diffraction data collection 10
Fluorophore concentrations 10
Water-Dioxane titration 11
Quantum yield 11
Extinction coefficients 11
Fluorescence increase upon protein binding assay 11
Affinity measurement by fluorescence polarisation assays 12
Stopped-Flow kinetic fluorescence polarisation assay 12
Cell biology and microscopy 13
Stable cell-line establishment 13
Neuron preparation and maintenance 13
rAAV production and neuron transduction 13
Cell fixation and staining 13
Live-cell staining 13
Flow cytometry 13
Confocal Fluorescence Microscopy 14
Wide-field Fluorescence Microscopy 14
PAINT Imaging 15
PAINT-Image processing and determination of single-molecule binding kinetics 15
MINFLUX Microscopy 16
Live-cell STED Microscopy 16
Statistical analysis and reproducibility 17

SUPPLEMENTARY FIGURES 21

Figure S. 1 | Computational screening of xHTLs candidates. 21
Figure S. 2 | Structural analysis of the TMR-S5/HaloTag7 complex. 22
Figure S. 3 | Structural comparison of the TMR-S5 docking results with the TMR-S5/HaloTag7 X-ray structure. 23
Figure S. 4| Structural analysis of the TMR-T5/HaloTag7 complex. 24
Figure S. 5 | Spectral and fluorogenic property tuning of xHTLs. 25
Figure S. 6 | Live-cell staining characterization of TMR-xHTLs. 25
Figure S. 7 | Live-cell staining compatibility of SiR-xHTLs with different cellular targets and cell types. 26
Figure S. 8 | xHTL live-cell staining characterization 27
Figure S. 9 | Live-cell labeling kinetics 27
Figure S. 10 | Comparison of HaloTag-based PAINT and DNA-PAINT. 28
Figure S. 11 | Characterization of xHTLs performance in MINFLUX microscopy. 29
Figure S. 12 | STED photobleaching resistance of xHTLs compared to covalent HTLs. 30
Figure S. 13 | Multi-frame STED imaging of xHTLs in rat hippocampal neurons 31
Figure S. 14 | Development of a second exchangeable xHTL/HaloTag7 protein pair. 32
Figure S. 15 | Structural analysis of the TMR-Hy5/dHaloTag7 complex. 33
Figure S. 16 | (d)HaloTag7-specific xHTLs Hy4/Hy5 are combinable with S5/T5. 34
Figure S. 17 | xHTL/HaloTag pairs for dual-color imaging 35
Figure S. 18 | Super-resolution fluorescence microscopy enabled by Hy4/5 and dHaloTag7. 36
Figure S. 19 | Dual-color super-resolution microscopy using xHTLs. 37

SUPPLEMENTARY TABLES 38

Table S. 1 | xHTL binding and spectral characterization 38
Table S. 2 | Data collection and refinement statistics for the crystal structure. 39
Table S. 3 | Fluorescence intensity increase of fluorogenic xHTL probes upon target binding. 40
Table S. 4 | Single-molecule binding kinetics and image resolution in HT-PAINT 40

PROTEIN SEQUENCES 40

NMR SPECTRA 41

REFERENCES 50

Supplementary Methods

Molecular docking

In-silico docking was conducted using the Schroedinger 2020-1 software package. All ligands were sketched with the 2D Sketcher module and diversified with the R-group creator and enumeration tool based on the TMR-CA lead-structure (**Fig. S1A**). The resulting compound libraries were processed in the Schroedinger ligprep module to obtain their 3D-geometry and most probable ionization states (Epik module, pH 7.4 ± 2.0). The crystal structure of HaloTag7 labeled with TMR-CA (PDB ID: 6Y7A, **Fig. S1B**)[1] was prepared in the Prime module. Default settings (pH value 7.4) were used including removal of crystallization ligands and water molecules. A cubic 800 nm^3 docking grid was generated around the HaloTag7 ligand molecule (reacted TMR-CA) and subsequent docking was performed using Glide[2] in standard precision configuration yielding up to 10 docking poses per ligand. During the docking process, polar interactions with the amino acids N41, D106 and W107 were applied as constrains.

Initial docking results were filtered by their glide score (gscore), which rewards favorable interactions and penalizes steric clashes using a static protein model. Docking results were assessed by their predicted binding energy (*i.e.* ΔG_{bind}, Prime MM-GBSA module) using the zwitterionic open form of TMR and in comparison to TMR-CA ($\Delta\Delta G = {}^{TMR\text{-}CA}\Delta G - {}^{TMR\text{-}candidate}\Delta G$, **Fig. S1C**). For high accuracy, ΔG_{bind} was calculated from induced-fit docking[3] with a final subset of candidates, giving a higher degree of flexibility to residues located in the ligand area: W107, F149, F205, P206, L209. Maestro was used for inspection of favourable protein/ligand interactions and PyMOL[4] software for presentation of protein/ligand poses.

Chemical Procedures

All chemical reagents and solvents for synthesis were purchased from commercial suppliers (Acros, Fluka, Merck KGaA, Roth, Sigma-Aldrich, TCI, TOCRIS) and used without further purification. Water-free solvents were stored over molecular sieves and used directly from a sealed-bottle. 6-Carboxyrhodamine derivatives were either prepared according to literature procedures by V. Nasufovic, B. Réssy, D. Schmidt (all MPIMF, Heidelberg) or purchased from commercial sources (Sigma-Aldrich, AAT Bioquest, **Method Table 1**).

Reactions performed under air and moisture exclusion were carried out in heat-dried glassware and under inert argon atmosphere using Schlenk techniques. Evaporation *in vacuo* was achieved at 40 °C and 10 – 850 mbar at a rotary evaporator (Heidolph) or by lyophilization on a lyophilizer (Christ) equipped with a vacuum pump (Vacuubrand). Reaction progress was monitored by analytical thin-layer chromatography (TLC) or liquid chromatography coupled to mass spectrometry (LC-MS). TLC was accomplished on commercially available SiO_2-plates (POLYGRAM® SIL G/UV254, 0.2 mm layer pre-coated polyester sheet, 40 x 80 mm, Roth) in appropriate solvent mixtures and visualized by using 254/366 nm UV-light or staining solutions ($KMnO_4$, Ninhydrin, Vanillin) and gentle heating. LC-MS was performed on a LCMS2020 (Shimadzu) connected to a Nexera UHPLC system. Column: C_{18} 1.7 µm, 50 × 2.1 mm (ACQUITY UPLC BEH, Waters). Buffer A: 0.1% Formic acid (FA)/MiliQ® water (ddH_2O), buffer B: acetonitrile (MeCN). Typical gradient was from 10% to 90% B within 6 min with 0.5 mL min^{-1} flow.

Method Table 1. Source and spectral properties according to literature of fluorophores used in this work.

Fluorophore	reference	source	λ_{abs} [nm]	ε [$M^{-1}\cdot cm^{-1}$]
JF_{525}	Grimm *et al.* (2017)[5]	i	525	138,000[b]
TMR	Mudd *et al.* (2015)[6]	AAT Bioquest	555	89,000[a]
MaP555	Wang *et al.* (2020)[7]	i	558	142,000[b]
JF_{585}	Grimm *et al.* (2017)[5]	Sigma-Aldrich	585	52,500[b]
CPY	Butkevich *et al.* (2016)[8]	AAT Bioquest	616	152,000[b]
MaP618	Wang *et al.* (2020)[7]	ii	616	5,500[b]
JF_{635}	Grimm *et al.* (2017)[5]	Sigma-Aldrich	635	17,000[b]
SiR	Lukinavičius *et al.* (2013)[9]	AAT Bioquest	646	120,000[b]
JF_{646}	Grimm *et al.* (2015)[10]	i	646	106,000[b]
SiR700	Lukinavičius *et al.* (2016)[11]	i	687	100,000[b]

a In PBS pH 7.4, **b** in activity buffer + 0.1% SDS

i provided by B. Réssy / D. Schmidt, **ii** provided by V. Nasufovic.

Flash column purification was performed using a Biotage (Isolera™ One) flash system equipped with pre-packed SiO_2 columns (SiliaSep™ Flash Cartridges, 40 – 63 µm, 60 Å). Depending on the batch size 12 g, 25 g or 40 g columns with 37, 75 or 100 mL min^{-1} flow rate were used. Typical gradients were 10 to 50% ethyl acetate in n-hexane or 1 to 10% methanol in dichloromethane (DCM) within 10 column volumes (CV). Small-scale preparative reversed-phase high-performance liquid chromatography (RP-HPLC) was carried out on an UltiMate 3000 system (Thermo Fisher Scientific). Column: C_{18} 5 µm, 21.2 × 250 mm (Supelco). Buffer A: 0.1% TFA in MiliQ® water, buffer B: MeCN. Typical gradient was from 20% to 90% B within 45 min with 8 mL min^{-1} flow. A 2998 PDA detector allows automated product collection based on the absorption wavelength of fluorescent labels (at 280, 550, 620 or 650 nm). Large-scale RP-HPLC-MS purification (> 3 mg) was carried-out with a LCMS-

2020 unit (Shimadzu) coupled with a prominence LC-20AP UFLC (Shimadzu). Column: C_{18} 5 µm, 30 × 250 mm (Shimadzu). Buffer A: 0.1% FA in ddH_2O, buffer B: MeCN. Typical gradient was from 10% to 90% B within 45 min with 20 mL min^{-1} flow. UV/Vis absorption was recorded using a SPD-M20A UV-VIS photodiode array detector and the desired compounds were collected based on the calc. mass-to-charge ratio (m/z) using a DUIS-2020 dual ion source (Shimadzu).

^{1}H-, ^{13}C- and ^{19}F-NMR spectra were recorded in deuterated solvents on a *Bruker Avance* III HD 400 NMR spectrometer at 298 K at 400 MHz (^{1}H), 101 MHz (^{13}C) or 377 MHz (^{19}F). Chemical shifts are expressed as parts per million (ppm, δ) and referenced to the solvent signals (^{1}H / ^{13}C) as internal standards: $CDCl_3$ (7.26 / 77.16 ppm), CD_3OD (3.31 / 49.00 ppm), DMSO-d_6 (2.05 / 39.52) and MeCN-d_3 (1.94 / 118.26 ppm) while ^{19}F-signals were not referenced. Coupling constants J are reported in Hz. Signal descriptions include: s = singlet, d = doublet, t = triplet, q = quartet, p =pentet, s – sextet, h – heptet and m = multiplet. ^{1}H-qNMR was performed using dry 1,4-dioxane (Acros, 3.57 ppm in DMSO-d_6, 8H) as a reference standard to quantify fluorophore concentrations. HRMS validation of synthesized chemical compounds was performed on a Bruker maXis II™ ETD spectrometer with electron spray ionization (ESI) by the *Mass Spectrometry* facility (MPIMF, Heidelberg).

S5 / T5-xHTL

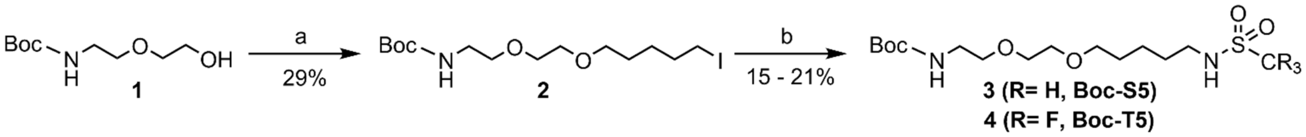

Method Figure 1. Synthetic route to xHTL precursor Boc-S5 (3) and Boc-T5 (4).
a. 1,5-Diiodopentan, NaH, THF/DMF, 3 h, 0 °C to rt. **b.** Methane sulfonamide or trifluoromethane sulfonamide, acetone, K_2CO_3 overnight, 60 °C. Boc: -COO-C(CH_3)$_3$.

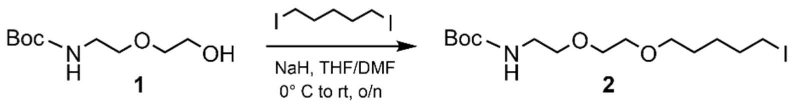

***tert*-Butyl-*N*-[2-[2-(5-iodopentyloxy)ethoxy]ethyl]carbamate (2):** A heat-dried reaction tube was charged with 200 mL of a 2/1 ratio (v/v) of dry tetrahydrofuran (THF) and *N*,*N*-dimethylformamide (DMF) under Schlenk conditions. *tert*-Butyl(2-(2-hydroxyethoxy)ethyl)carbamate (**1**, BocNH-PEG$_2$-OH, 2 mL, 10.4 mmol, 1 eq.) was added and dissolved under vigorous stirring. The mixture was cooled to 0 °C and NaH (455 mg, 60% immersion on mineral oil, 13.8 mmol, 1.1 eq.) was added portion-wise. The evolving gas was carefully released and the mixture was left stirring at 0 °C for 30 min under an inert gas atmosphere. 1,5-Diiodopentan (6.4 mL, 41.4 mmol, 4 eq.) was added directly into the suspension at 0 °C, the reaction was warmed to rt and left stirring overnight. 10 mL aqueous 10% NH_4Cl and EtOAc were added and the aq. layer was extracted three times with 100 mL EtOAc. The org. layers were combined and washed with brine once and aq. 10% LiCl three times, dried over $MgSO_4$, filtered and the solvent was removed. Flash column chromatography (SiO_2. 20 to 80% EtOAc in n-hexane in 12 CV) afforded the desired compound **2** (1.2 g, 3.0 mmol, 29 %) as a colorless oil. **^{1}H NMR** (400 MHz, $CDCl_3$): δ 3.46 – 3.35 (m, 6H), 3.30 (t, *J* = 6.5 Hz, 2H), 3.19 – 3.11 (m, 2H), 3.02 (t, *J* = 7.0 Hz, 2H), 1.68 (dt, *J* = 14.5, 7.1 Hz, 2H), 1.50 – 1.39 (m, 2H), 1.36 – 1.29 (m, 2H), 1.27 (s, 9H). Spectrum 1 shown at end of SI. **^{13}C NMR** (101 MHz, $CDCl_3$): δ 155.96, 79.18, 71.07, 70.27, 70.23, 70.08, 40.36, 33.30, 28.53, 28.44 (3C), 27.16, 6.85. **HRMS** (m/z): [M + Na]$^+$ calcd. for $C_{14}H_{28}NO_4I$, 402.1136; found, 402.1136.

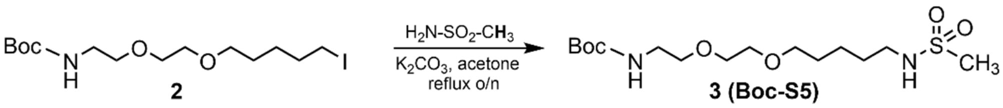

***tert*-Butyl (2-(2-((5-(methylsulfonamino)pentyl)-oxy)ethoxy)ethylcarbamate** (**3**, Boc-S5): Compound **2** (200 mg, 0.5 mmol, 1 eq.), potassium carbonate (276 mg, 2.0 mmol, 4 eq.) and methane sulfonamide (57 mg, 0.6 mmol, 1.2 eq.) was dissolved in 5 mL dry acetone in a dry round-bottom flask. The mixture was stirred at 60 °C overnight and purified by flash column chromatography (SiO_2. 20 to 80% EtOAc in n-hexane in 8 CV) to afford Boc-S5 (30 mg, 0.08 mmol, 15%) as a pale-yellow oil. **^{1}H NMR** (400 MHz, $CDCl_3$): δ 3.62 – 3.48 (m, 6H), 3.44 (t, *J* = 6.3 Hz, 2H), 3.29 (q, *J* = 6.6, 5.7 Hz, 2H), 3.10 (q, *J* = 6.8 Hz, 2H), 2.92 (s, 3H), 1.65 – 1.52 (m, 4H), 1,46 – 1.35 (m, 2H), 1.44 (s, 9H). Spectrum 2 shown at end of SI. **^{13}C NMR** (101 MHz, $CDCl_3$): δ 156.06, 79.25, 77.26, 70.98, 70.22, 70.06, 43.15, 40.28, 29.83, 28.91, 28.44 (3C), 23.19, 21.3. **HRMS** (m/z): [M + Na]$^+$ calcd. for $C_{16}H_{32}N_2O_6SNa^+$, 369.2051; found, 369.2051.

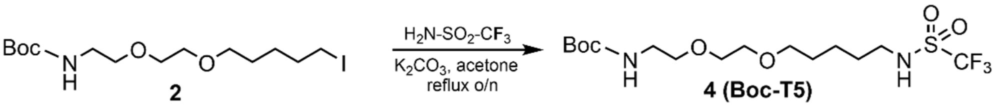

***tert*-Butyl (2-(2-((5-(trifluoromethylsulfonamino)-pentyl)oxy)ethoxy)ethylcarbamate** (**4**, Boc-T5): Compound **2** (200 mg, 0.5 mmol, 1 eq.), potassium carbonate (276 mg, 2.0 mmol, 4 eq.) and trifluoromethane sulfonamide (57 mg, 0.6 mmol, 1.2 eq.) was dissolved in 5 mL dry acetone in a dry round-bottom flask. The mixture was stirred at 60 °C overnight and purified by flash column chromatography (SiO_2. 10 to 60% EtOAc in n-hexane in 10 CV) to afford Boc-T5 (50 mg, 0.1 mmol, 25%) as a pale-yellow oil. **^{1}H NMR** (400 MHz, $CDCl_3$): δ 3.65 – 3.51 (m, 6H), 3.49 (t, *J* = 5.8 Hz, 2H), 3.30 (q, *J* = 6.3 Hz, 4H), 1.69 – 1.59 (m, 4H), 1,50 (td, *J* = 9.6, 7.0, 3.5 Hz, 2H), 1.44 (s, 9H). Spectrum 3 shown at end of SI. **^{13}C NMR** (101 MHz, $CDCl_3$): δ 156.34, 124.50 (q, CF_3), 77.36, 77.48, 70.99, 70.27 (3C), 60.58, 44.30, 29.83, 28.50 (4C), 23.2. **HRMS** (m/z): $[M + Na]^+$ calcd. for $C_{16}H_{29}N_2O_6SF_3Na^+$, 445.1591; found, 445.1587.

Hy4 / Hy5-xHTL

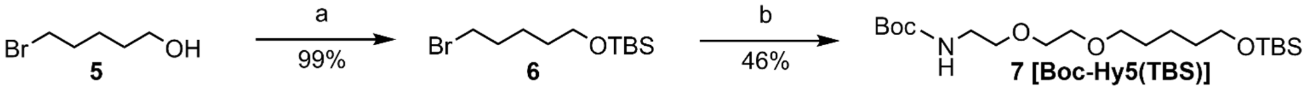

Method Figure 2 Synthetic route to xHTL precursor Boc-Hy5(TBS) (7).
a. TBS-Cl, DCM, imidazole, 1 h, 0 °C to rt. **b.** BocNH-PEG$_2$-OH, NaH, THF/DMF, o/n, 0 °C to rt.

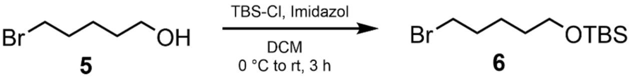

***tert*-Butyl (5-bromopentoxy)-dimethylsilane (6):** 1-Bromopentan-5-ol (**5**, 1.5 g, 9 mmol, 1 eq.) was dissolved in 20 mL dry DCM and imidazole (930 mg, 13.5 mmol, 1.5 eq.) was added. The mixture was cooled to 0 °C under vigorous stirring and *tert*-butyldimethylsilyl chloride (TBS-Cl, 1.5 g, 10 mmol, 1.1 eq.) was added portion-wise. After 1 h stirring at rt 10 mL aqueous 10% NH_4Cl, the aq. layer was extracted with 50 mL DCM. The org. layers were combined and washed with 100 mL brine, dried over $MgSO_4$, filtered and the solvent was removed. Flash column purification (SiO_2. 0 to 30% EtOAc/n-hexane in 8 CV) afforded **6** (2.5 g, 9 mmol, 99%) as a colorless liquid. **^{1}H NMR** (400 MHz, $CDCl_3$): δ 3.61 (t, *J* = 6.1 Hz, 2H), 3.41 (t, *J* = 6.8 Hz, 2H), 1.88 (m, 2H), 1.52 (m, 4H), 0.89 (s, 9H), 0.05 (s, 6H). **^{13}C NMR** (101 MHz, $CDCl_3$): δ 62.99, 33.96, 32.76, 32.05, 26.01 (3C), 24.73, -5.15 (2C). **HRMS** (m/z): $[M + H]^+$ calcd. for $C_{11}H_{25}BrOSi^+$, 281.0931, 283.0911; found, 281.0933, 283.0913.

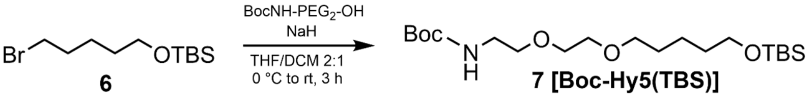

***tert*-Butyl (2,2,3,3-tetramethyl-4,8,11-trioxa-3-silapentadecan-15-yl)carbamate** (**7**, Boc-Hy5-TBS): The title compound was synthesized similarly as described for (**2**) from **6** (2 g, 7.1 mmol, 1 eq.) and afforded **Boc-Hy5-TBS** (1.3 g, 3.3 mmol, 46%) as a colorless liquid. **^{1}H NMR** (400 MHz, $CDCl_3$): δ 3.62 – 3.49 (m, 8H), 3.44 (t, *J* = 6.8 Hz, 2H), 3.29 (q, *J* = 5.3 Hz, 2H), 1.69 – 1.55 (m, 2H), 1.55 – 1.46 (m, 2H), 1.42 (s, 9H), 1.39 – 1.28 (m, 2H), 0.87 (s, 9H), 0.02 (s, 6H). Spectrum 4 shown at end of SI. **^{13}C NMR** (101 MHz, $CDCl_3$): δ 156.11, 71.57, 50.39, 70.32, 70. 12, 40.46, 37.75, 29.49, 28.58 (3C), 26.08 (3C), 22.45, 18.46, -5.17 (2C). **HRMS** (m/z): $[M + H]^+$ calcd. for $C_{20}H_{43}NO_5Si^+$, 406.2983; found, 406.2983.

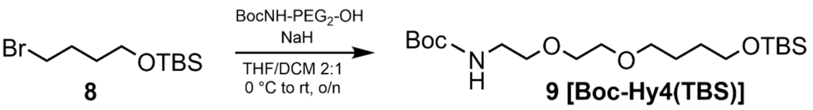

***tert*-Butyl (2,2,3,3-tetramethyl-4,8,11-trioxa-3-silatetradecan-14-yl)carbamate** (**9**, Boc-Hy4-TBS): The title compound was synthesized similarly as described for (**2**) from commercially available *tert*-butyl (3-bromobutoxy)dimethylsilane (**8**, 2 mL, 7.2 mmol, 1 eq.) with the difference that the reaction required 16 h until complete conversion of the starting material and afforded **Boc-Hy4-TBS** (820 mg, 2.7 mmol, 38%) as a colorless liquid. **^{1}H NMR** (400 MHz, $CDCl_3$): δ 3.66 – 3.51 (m, 8H), 3.48 (t, *J* = 6.5 Hz, 2H), 3.31 (s, 2H), 1.69 – 1.61 (m, 2H), 1.60 – 1.53 (m, 2H), 1.44 (s, 9H), 0.89 (s, 9H), 0.04 (s, 6H), Spectrum 5 shown at end of SI. **HRMS** (m/z): $[M + Na, \Delta TBST]^+$ calcd. for $C_{13}H_{27}NO_5Na$, 300,1718; found 300,1718.

Deprotection

For Boc and TBS protecting groups removal, compound **3**, **4**, **7** or **9** were dissolved in 3 mL/mmol of a 1/1 (v/v) mixture of trifluoroacetic acid (TFA) in dry DCM. It was stirred at rt for at least 3 h and the solvent was removed under a stream of argon gas. Residual TFA was removed by co-evaporation with DCM. Resulting xHTLs were used for fluorophore coupling without further purification.

***N*-(5-(2-(2-aminoethoxy)ethoxy)pentyl)methanesulfonamide** (**10, S5**). **^{1}H NMR** (400 MHz, MeOD): δ 3.65 – 3.50 (m, 6H), 3.43 (t, *J* = 6.4 Hz, 2H), 3.04 (t, *J* = 5.0 Hz, 2H), 2.97 (t, *J* = 6.8 Hz, 2H), 1.58 – 1.43 (m, 4H), 1.41 – 1.31 (m, 2H). **^{13}C NMR** (101 MHz, MeOD): δ 71.53, 70.68, 70.50, 67.22, 43.26, 40.04, 38.91, 30.26, 29.47, 23.56. **HRMS** (m/z): $[M + H]^+$ calcd. for $C_{10}H_{24}N_2O_4S$, 269.1530 found, 269.1528.

***N*-(5-(2-(2-aminoethoxy)ethoxy)pentyl)trifluoromethanesulfonamide** (**11, T5**). **^{1}H NMR** (400 MHz, $CDCl_3$): δ 3.70 – 3.58 (m, 6H), 3.51 (t, *J* = 6.2 Hz, 2H), 3.21 (t, *J* = 5.2, 2H), 3.12 (t, 6.4 Hz, 2H), 1.62 – 1.54 (m, 4H), 1.48 – 1.42 (m, 2H). **^{13}C NMR** (101 MHz, $CDCl_3$): δ 121.50 (q, CF_3),70.69, 69.92, 69.77, 68.47, 43.48, 39.28, 29.66, 28.61, 22.52. **^{19}F NMR** (377 MHz, $CDCl_3$) δ 79.52. **HRMS** (m/z): $[M + H]^+$ calcd. for $C_{10}H_{21}F_3N_2O_4S$, 323.1243 found, 323.1247.

5-(2-(2-aminoethoxy)ethoxy)pentan-1-ol (12, Hy5). **^{1}H NMR** (400 MHz, MeOD): δ 4.28 (t, J = 6.6 Hz, 2H), 3.70 – 3.61 (m, 2H), 3.57 (dd, *J* = 5.8, 2.9 Hz, 2H), 3.50 (dd, *J* = 5.7, 3.0 Hz, 2H), 3.40 (t, *J* = 6.6 Hz, 2H), 3.09 (s, 2H), 1.69 (dt, *J* = 14.9, 6.8 Hz, 2H), 1.54 (q, *J* = 7.1 Hz, 2H), 1.40 – 1.28 (m, 2H). **^{13}C NMR** (101 MHz, MeOD): δ 71.01, 70.31, 69.90, 68.16, 39.70, 28.89, 27.85, 22.18. **HRMS** (m/z): $[M + H]^+$ calcd. for $C_9H_{21}NO_3$, 192.1594 found, 192.1593.

5-(2-(2-aminoethoxy)ethoxy)butan-1-ol (13, Hy4). **^{1}H NMR** (400 MHz, MeOD): δ 3.72 – 3.61 (m, 6H), 3.57 (t, J = 6.2 Hz, 2H), 3.53 (t, *J* = 6.2 Hz, 2H), 3.15 – 3.11 (m, 2H), 1.72 – 1.54 (m, 4H). **HRMS** (m/z): $[M + H]^+$ calcd. for $C_8H_{19}NO_3$, 178.1438 found, 178.1439.

Fluorophore coupling

For fluorophore coupling, 6-carboxy rhodamines (1-5 mg, 1 eq.) were dissolved in ~100 µL/µmol dry DMSO-d_6. The mixture was added to *N*,*N*,*N*′,*N*′-tetramethyl-*O*-(*N*-succinimidyl)uroniumtetrafluorborat (TSTU, 1.2 eq.) and diisopropylethylamine (DIPEA, 10 eq.) were added to the solution. It was left stirring for at least 10 min, the xHTL **14** - **17** were dissolved in the same amount of dry DMSO-d_6 with 10 eq. DIPEA and both solutions were mixed and left stirring for 2 h at 40 °C. The desired products were obtained by RP-HPLC using a H_2O/MeCN (containing 0.1% TFA) linear gradient from 20 to 90%.

TMR-S5 – **^{1}H NMR** (400 MHz, CD_3CN): δ 8.11 (d, J = 8.1 Hz, 1H), 8.05 (dd, J = 8.1, 1.5 Hz, 1H), 7.58 (d, J = 1.5 Hz, 1H), 7.32 (t, J = 5.6 Hz, 1H), 6.78 (d, J = 8.8 Hz, 2H), 6.65 – 6.60 (m, 4H), 3.57 – 3.49 (m, 4H), 3.48 – 3.43 (m, 4H), 3.32 (t, J = 6.4 Hz, 2H), 3.06 (s, 12H), 2.95 (q, J = 6.7 Hz, 2H), 2.82 (s, 3H), 1.44 (h, J = 6.8, 6.4 Hz, 4H), 1.33 – 1.22 (m, 2H). Spectrum 6 shown at end of SI.

TMR-T5 – **^{1}H NMR** (400 MHz, CD_3CN): δ 8.28 (d, J = 8.1 Hz, 1H), 8.08 (dd, J = 8.2, 1.7 Hz, 1H), 7.69 (d, J = 1.7 Hz, 1H), 7.40 (t, J = 5.6 Hz, 1H), 7.04 (d, J = 9.4 Hz, 2H), 6.86 (dd, J = 9.4, 2.5 Hz, 2H), 6.79 (d, J = 2.5 Hz, 2H), 3.62 – 3.44 (m, 8H), 3.35 (t, J = 6.4 Hz, 2H), 3.20 (s, 12H), 1.57 – 1.40 (m, 4H), 1.36 – 1.24 (m, 4H). Spectrum 7 shown at end of SI.

TMR-Hy5 – **^{1}H NMR** (400 MHz, CD_3CN): δ 8.24 (d, *J* = 8.2 Hz, 1H), 8.09 (dd, *J* = 8.1, 1.7 Hz, 1H), 7.67 (d, *J* = 1.7 Hz, 1H), 7.36 (d, *J* = 5.7 Hz, 1H), 6.97 (d, *J* = 9.3 Hz, 2H), 6.81 (dd, *J* = 9.3, 2.5 Hz, 2H), 6.75 (d, *J* = 2.5 Hz, 2H), 3.60 – 3.44 (m, 7H), 3.42 (t, *J* = 6.4 Hz, 2H), 3.35 (t, *J* = 6.5 Hz, 2H), 3.17 (s, 12H), 1.43 (tt, *J* = 14.2, 6.4 Hz, 4H), 1.35 – 1.18 (m, 4H). Spectrum 8 shown at end of SI.

TMR-Hy4 – **^{1}H NMR** (400 MHz, CD_3CN): δ 8.29 (d, J = 8.2 Hz, 1H), 8.11 (dd, J = 8.2, 1.8 Hz, 1H), 7.71 (d, J = 1.8 Hz, 1H), 7.55 (t, J = 5.7 Hz, 1H), 7.05 (d, J = 9.4 Hz, 2H), 6.88 (dd, J = 9.4, 2.5 Hz, 2H), 6.81 (d, J = 2.4 Hz, 2H), 3.63 – 3.45 (m, 8H), 3.34 (t, J = 6.7 Hz, 2H), 3.21 (s, 12H), 1.47 – 1.38 (m, 2H), 1.30 – 1.20 (m, 2H). Spectrum 9 shown at end of SI.

Method Table 2. HRMS data of fluorophore derivatized xHTLs.

xHTL	Dye	Chem. Form.	$[M]_{calcd.}$	$[M]_{found}$	xHTL	Dye	Chem. Form.	$[M]_{calcd.}$	$[M]_{found}$
S5	JF_{525}	$C_{37}H_{41}N_4O_8F_4S$	777.2575	777.2575	Hy5	JF_{525}	$C_{36}H_{38}N_3O_7F_4$	700.2640	700.2640
	TMR	$C_{35}H_{45}N_4O_8S$	681.2953	681.2952		TMR	$C_{34}H_{42}N_3O_7$	604.3017	604.3016
	MaP555	$C_{37}H_{51}N_{46}O_9S_2$	787.3154	787.3152		MaP555	$C_{36}H_{47}N_5O_8S$	710.2118	710.2120
	JF_{585}	$C_{40}H_{47}N_4O_7F_4S$	803.3096	803.3097		CPY	$C_{37}H_{47}N_3O_6$	630.3538	630.3538
	CPY	$C_{38}H_{51}N_4O_7S$	707.3473	707.3473		MaP618	$C_{39}H_{53}N_5O_7S$	736.3738	736.3735
	MaP618	$C_{40}H_{51}N_6O_8S_2$	813.3674	813.3652		SiR	$C_{36}H_{48}N_3O_6Si$	646.3307	646.3306
	JF_{635}	$C_{39}H_{49}N_4O_7F_2SSi$	783.3054	783.3048		SiR700	$C_{38}H_{48}N_3O_6Si$	670.3307	670.3296
	JF_{646}	$C_{39}H_{51}N_4O_7SSi$	747.3242	747.3239		JF_{525}	$C_{36}H_{38}N_3O_7F_4$	700.2640	700.2640
	SiR	$C_{37}H_{51}N_4O_7SSi$	723.3242	723.3240	Hy4	TMR	$C_{33}H_{40}N_3O_7Na^+$	444.0431	444.0428
	SiR700	$C_{39}H_{51}N_4O_7SSi$	747.3242	747.3247		JF_{585}	$C_{38}H_{42}N_3O_6F_4$	712.3004	712.3005
T5	JF_{525}	$C_{37}H_{38}N_4O_8F_7S$	831.2290	831.2293		CPY	$C_{36}H_{46}N_3O_6$	616.3381	616.3378
	TMR	$C_{35}H_{42}N_4O_8F_3S$	735.2665	735.2670		MaP618	$C_{38}H_{51}N_5O_7S$	722.3582	722.3579
	MaP555	$C_{37}H_{48}N_6O_9F_3S_2$	841.2871	841.2870		JF_{635}	$C_{37}H_{44}N_3O_6F_2Si$	692.2962	692.2945
	JF_{585}	$C_{40}H_{44}N_4O_7F_7S$	857.2813	857.2813		JF_{646}	$C_{37}H_{46}N_3O_6Si$	659.3158	659.3150
	CPY	$C_{38}H_{48}N_4O_7SF_3$	761.3190	761.3193		SiR	$C_{35}H_{46}N_3O_6Si$	632.3150	632.3153
	SiR	$C_{37}H_{48}N_4O_7SF_3Si$	777.2966	777.2960		SiR700	$C_{37}H_{46}N_3O_6Si$	656.3150	656.3148

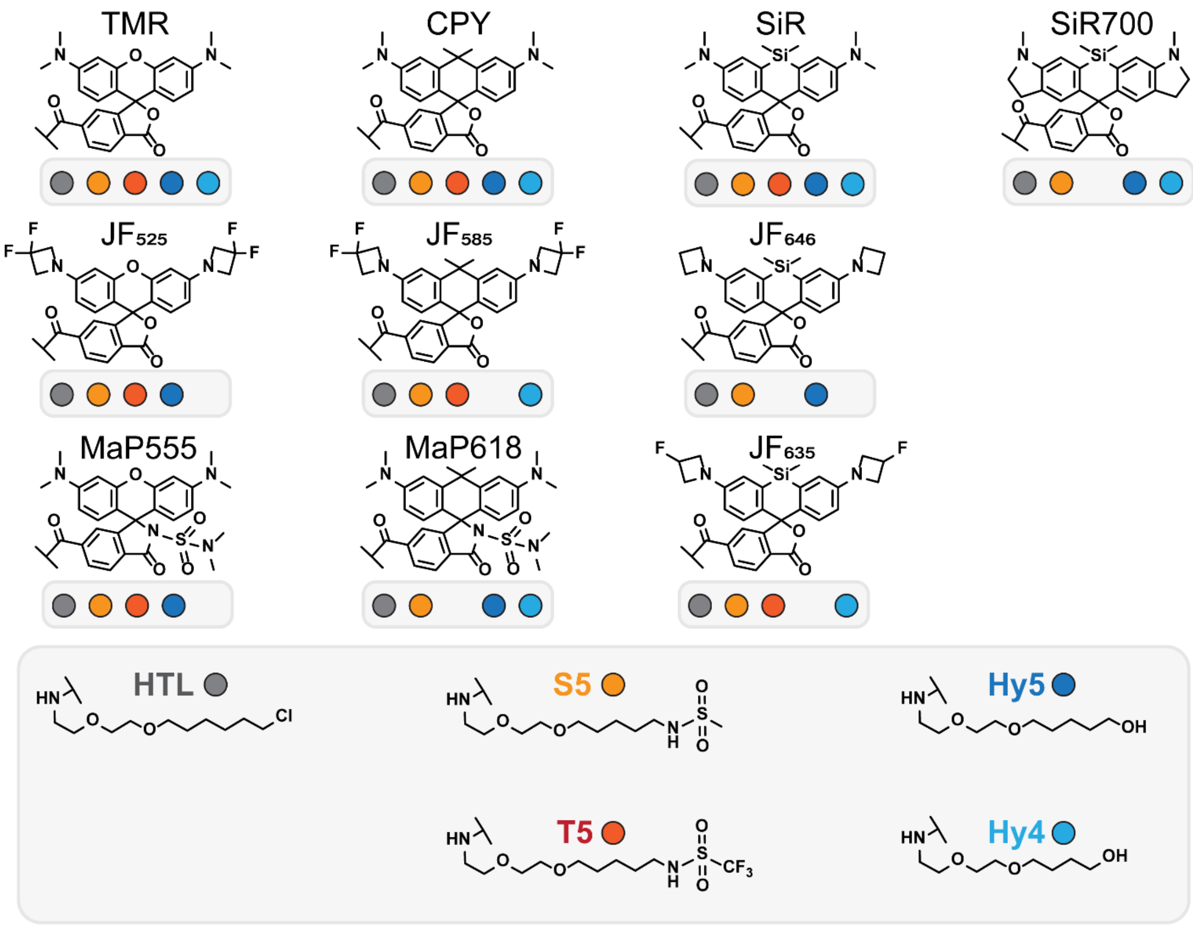

Method Figure 3. Chemical structures of fluorophore-derivatives of different HaloTag ligands (x)HTLs. Coloured dots indicate which fluorophore derivatives were used in this study.

Molecular biology & Biochemistry

Plasmids

HaloTag7 and its inactive ("dead") variant (HaloTag7-D106A = dHaloTag7)[12] were produced in *Escherichia coli* using a pET51b(+) expression vector (Novagen) carrying a N-terminal His_{x10}-tag and a tobacco etch virus (TEV)[13] protease cleavage site, as previously reported.[1] Mammalian protein expression was performed using the pcDNA5/FRT/TO expression vector (ThermoFisher Scientific). HaloTag variants were fused to different sub-cellular markers (H2B, CEP41, TOM20, COX8, CalR/KDEL, LAMP1, Ig-κ-/PDGFR or LifeAct) whose origins are reported in **Method Table 8**.[1, 14, 15] Proteins were co-expressed by translational fusion using the self-cleaving peptide sequence T2A.[16, 17] Molecular cloning was performed by Gibson assembly.[18] dHaloTag variant was obtained by introducing a point-mutation in HaloTag7 with the Q5®-site-directed mutagenesis kit (NEB) according to the manufacturer protocol. Plasmid sequences were verified by Sanger sequencing (Eurofins) and plasmid DNA was stored at -20 °C.

Protein production and purification

pET51b(+) plasmids were transformed in *E. coli* strain BL21(DE3)-pLysS (Novagen) and cultured at 37 °C in LB^{Amp} (**Method Table 3**) until an optical density at 600 nm (OD_{600}) of 0.8 was reached. Then, the temperature was reduced to 18 °C and transgene expression was induced with 0.5 mM isopropyl β-thiogalacopyranoside (IPTG). After overnight expression, cells were harvested by centrifugation (4 000 g, 15 min, 4 °C), resuspended in ice-cold extraction buffer (**Method Table 3**) including 1 mM phenylmethylsulphonyl fluoride (PMFS) and 0.25 mg/mL lysozyme. Cells were lyzed by sonication (SONOPLUS, 7 min, 50% on/off cycles, 70% amplitude) on wet ice. The lysate was cleared from the cell debris by centrifugation (20 min, 50 000 g, 4 °C). Proteins were purified via immobilized metal ion affinity chromatography (IMAC) using a HisTrap FF crude column (Cytiva, Marlborough, MA) and an ÄktaPure FPLC instrument (Cytiva) with IMAC wash and elution buffers (**Method Table 3**). Buffer was exchanged to activity buffer on a HiTrap® 26/10 Desalting Column (Cytiva) (**Method Table 3**) using the ÄktaPure FPLC.

Method Table 3. Buffer and media composition.

Buffer	Composition
LB^{Amp}	5 g/L yeast extract, 10 g/L peptone, 0.1 g/L ampicillin
Activity buffer	50 mM HEPES, 50 mM NaCl, pH 7.3
Extraction buffer	50 mM KH_2PO_4, 300 mM NaCl, 5 mM imidazole, pH 8.0
IMAC wash buffer	50 mM KH_2PO_4, 300 mM NaCl, 10 mM imidazole, pH 7.5
IMAC elution buffer	50 mM KH_2PO_4, 300 mM NaCl, 500 mM imidazole, pH 7.5
TEV-cleavage buffer	25 mM Na_2HPO4, 200 mM NaCl, pH 8.0
Laemmli Buffer	31.5 mM Tris-HCl, pH 6.8, 10% glycerol, 1% SDS, 0.005% Bromophenol Blue

Buffers/media were prepared in Milli-Q® water.

The protein solution was concentrated with Ultra 15 mL Centrifugal Filters (Amicon®, MWKO: 10 kDa). Proteins were quantified by UV-absorption at 280 nm (NanoDrop™ 2000c, ThermoFisher) using the molar extinction coefficient ε of (d)HaloTag7 (60 055 M^{-1} cm^{-1}) and concentration was adjusted to 500 µM with activity buffer. Finally, proteins were aliquoted, flash frozen in liquid nitrogen and stored at -80 °C. Furthermore, the correct molecular weight and purity of proteins were assessed by sodium dodecyl sulfate–polyacrylamide gel electrophoresis (SDS–PAGE). ~50 µg of isolated protein was denatured in Laemmli sample buffer (BioRad with 10 mM dithiothreitol (DTT)) at 95 °C for 10 min, loaded onto a stain-free gel (mini-PROTEAN® TGX™ Stain-Free™, 4 - 20%, Bio-Rad) besides a *PrecisionPlus Protein™ All Blue* (Bio-Rad) pre-stained marker and imaged on a ChemiDoc XRS+ device (Bio-Rad, **Method Table 4**). Additionally, the correct mass of purified proteins was confirmed by electrospray-ionization mass-spectrometry (ESI-MS) performed by the *Mass Spectrometry* facility (MPIMF, Heidelberg) on a maXis II ETD-instrument. Protein amino acid sequences are listed at the protein sequences section.

Covalent labeling of HaloTag7 protein and in-gel fluorescence scanning

Purified HaloTag7 protein (5 µM) was incubated in presence of 4x excess of the (x)HTLs (20 µM). Reaction was carried out in activity buffer for 30 min at 37 °C. 5 µg protein were separated by SDS-PAGE as previously explained.[1] Covalent labeling was revealed by in-gel fluorescence imaging performed on a ChemiDoc XRS+ imager (Bio-Rad) using either the green or red epi illumination (**Method Table 4**). These gels were further Coomassie stained (Bio-Rad) and imaged by white light converted UV trans illumination (**Method Table 4**) using the same imager.

Method Table 4. Imaging settings in-gel fluorescence

Channel	λ_{ext} [nm]	λ_{em} [nm]	Detection
Epi-red	520 - 545	605 ± 50	TMR
Epi-green	625 - 650	695 ± 5	SiR
Epi-white	/	590 ± 110	Coomassie

Protein crystallization and X-ray diffraction data collection

For protein crystallization, HaloTag7 and dHaloTag7 (D106A variant) proteins were produced and purified as previously described (carried out by A. Bergner)[1, 15]. In short, after IMAC purification the buffer was exchanged to TEV-cleavage buffer (**Method Table 3**). TEV protease[13] (weight ratio 1:30) were added to protein sample and cleavage was performed at 30 °C overnight. The solution was then filtered (0.22 µm) and the cleaved protein was harvested by reverse IMAC purification on a HisTrap FF crude column (Cytiva) on an ÄktaPure FPLC (Cytiva) as explained earlier but collecting the flow through. The protein was then concentrated and further purified by size exclusion chromatography on a HiLoad 26/600 Superdex 75 pg column (Cytiva) exchanging the buffer to activity buffer. The protein was concentrated again to a final concentration of 5 µM. 10 µM TMR-xHTL was added to the protein for at least 4 h at room temperature (rt). Afterwards, the protein was concentrated to ~250 µL reaching a final concentration between 10 – 15 mg/mL of HaloTag protein (quantified using the absorbance at 280 nm and the extinction coefficient of the protein corrected for TMR absorbance at 280 nm, $^{TMR}CF_{280nm}$ = 0.16, 13 920 M^{-1} cm^{-1}).

Crystallization was performed at 20 °C using the vapor-diffusion method. Crystals of HaloTag7 with S5-TMR ligands and dHaloTag7 (D106A variant) with Hy5-TMR ligands were grown by mixing equal volumes of protein solution and a reservoir solution containing 0.1 M MES pH 6.0, 1.0 M lithium chloride and 19-20% (m/v) PEG 6000. Crystals of HaloTag7 with T5-TMR ligand were obtained by mixing equal volumes of protein solution and precipitant solution composed of 0.1 M MES pH 6.0, 0.2 M calcium acetate, 21% (m/v) PEG 8000. In both cases, the crystals were briefly washed in cryoprotectant solution consisting of the reservoir solution with glycerol added to a final concentration of 20% (v/v), prior to flash-cooling in liquid nitrogen.

Single crystal X-ray diffraction data were collected at 100 K on the X10SA beamline at the SLS (PSI, Villigen, Switzerland). All data were processed with XDS[19]. The structures of HaloTag7 in complex with TMR ligands were determined by molecular replacement (MR) using Phaser[20] and HaloTag7-TMR coordinates (PDB ID: 6Y7A) as a search model. Geometrical restraints for TMR ligands were generated using the Grade server[21]. The final models were optimized in iterative cycles of manual rebuilding using Coot[22] and refinement using Refmac5[23] and phenix.refine[24]. Data collection and refinement statistics are summarized in **Table S2**, model quality was validated with MolProbity[25] as implemented in PHENIX. Atomic coordinates and structure factors have been deposited in the Protein Data Bank under accession codes: 7ZJ0 (HaloTag7 S5-TMR), 7ZIY (HaloTag7 T5-TMR) and 7ZIZ (dHaloTag7 Hy5-TMR).

To compare the similarity of ligand conformations found in xHTL/HaloTag docking or crystal structures, the root-mean-square deviation (rmsd) was calculated from the distance d of two superimposed structures from n atoms (**eq. 1**):

$$rmsd = \sqrt{\frac{1}{n}\sum d^2} \qquad (1)$$

rmsd: root-mean-square distance, d: distance between similar atoms, n: number of analyzed atoms.

Fluorophore concentrations

The concentrations of fluorophore-ligands were determined by UV-Vis absorption at their maximum absorption wavelength (**Method Table 1**) using the Lambert-Beer's law (**eq. 2**):

$$\mathrm{Abs} = c \cdot d \cdot \varepsilon \qquad (2)$$

Abs: absorption at λ_{max} [A.U.], c: concentration [M], d: pathway length [cm], ε extinction coefficient [1/(M cm)]

Absorbance was measured with a Nanodrop 2000c™ spectrophotometer (ThermoFisher) using 2 µL of solution (d = 0.1 cm) or 500 µL in a polystyrene cuvette (Sarstedt, 10 x 4 x 45 mm, d = 1 cm). Samples were prepared in PBS pH 7.4 (Gibco), 0.1% w/v SDS in activity buffer or 0.1% v/v TFA in ethanol, depending of the fluorophore properties and according to literature precedents (**Method Table 1**).[5, 26] In-house characterized extinction coefficients ε (for detailed description see below) were used to calculate the concentration of fluorophore ligands (see **Method Table 1, Method Figure 3**). Fluorescent molecules were prepared as stock solutions in dry DMSO (> 1 mM) and diluted such that the DMSO concentration never exceed 1% (v/v) in experiments.

Water-Dioxane titration

Solutions of SiR-(x)HTLs (5 µM, 100 µL) were prepared in 20/80 to 90/10 (v/v) water-dioxane mixtures (dry, Acros) in transparent flat-bottom, chimney well polypropylene 96-well plates (Greiner Bio-One) in technical triplicates. Absorbance spectra were recorded between 400 and 750 nm in a microplate reader (Spark20M, Tecan) with 2 nm step size. The maximal absorbance of SiR at 646 nm (λ_{max}) was blank corrected in each condition. The data was normalized to the max. absorbance measured (SiR-S5) and presented as the ratio of absorbance variations between 0 and 1. The data from three technical replicates was averaged and plotted against the dielectric constants ε_R of the water-dioxane mixtures.[27] A sigmoidal function (**eq. 3**) was fitted to the data yielding D_{50}-values as the point of inflection. Mean values and standard deviations from three independent experiments are presented.

$$Abs = \frac{1}{(1 + 10^{(D50-\varepsilon_R)})} \quad (3)$$

Abs: Norm. absorbance, ε_R: dielectric constant, D_{50}: ε_R at half-maximal absorbance.

Quantum yield

The fluorophore-xHTLs (500 nM) were incubated with or without 100 µM (d)HaloTag7 protein in activity buffer in 800 µL Glass Crimp Neck Vial (8 mm, Fisherbrand™, used for TMR, MaP555, CPY, MaP618) or in 2 mL Quarz cuvettes (used for JF_{635}, SiR) for 2 h at rt. Quantum yields were measured in technical triplicates by exciting the fluorophore at its maximum absorbance (λ_{abs}) (**Method Table 1**) in an absolute quantum yield spectrometer (Hamatsu, Quantaurs-QY, model C11347). Mean values and standard deviations from three individual measurements (n = 3) are given.

Extinction coefficients

Prior to extinction coefficient measurements the fluorophore-xHTL ligands were quantified by qNMR using a dioxane standard (5 µM). A dilution series of 0.5, 1, 1.5, 2 and 3 µM of fluorophore-xHTL was prepared in activity buffer, 0.1% SDS in activity buffer or in presence of 100 µM (d)HaloTag7 protein in a total volume of 200 µL (clear bottom non-binding 96-well plates, Greiner). Absorbance spectra were recorded on a plate reader (Spark20M, Tecan) from 400 to 700 nm. The data was baseline corrected and the maximum absorbance values were plotted against the concentration. A linear function (**eq. 4**) was fitted to the data and extinction coefficients were calculated from the slope b which was corrected for the path length using the Lambert-Beer's law (**eq 1.**). The path length in 96-well plates was obtained measuring the absorbance of SiR-CA in 0.1% SDS for which the extinction coefficient was previously reported.[9]

$$Abs = a + bx \quad (4)$$

Abs: absorbance [A.U.], x: fluorophore concentration [M].

Fluorescence increase upon protein binding assay

The fluorophore-(x)HTLs (50 nM) were incubated in presence and absence of (d)HaloTag7 protein (100 µM) for 30 min at 37 °C in activity buffer containing 1% (w/v) BSA in a black flat bottom 384 well plate (Greiner, 20 µL). Fluorescence emission scans were recorded by exciting the fluorophore and measuring the fluorescence emission intensity over a spectral range covering the maximum emission (2 nm step, 10 nm bandwidth) on a microplate reader (Spark20M, Tecan) using the settings summarized in **Method Table 5**. For representation, the fluorescence emission was normalized to the max. intensity measured for each dye (*e.g.* SiR-S5). The data from three technical replicates were averaged and the fluorescence intensity increase represented by their ratio in presence and absence of the respective HaloTag protein (F/F_0) at the fluorescence emission maxima was obtained (**Method Table 5**). The uncertainty was calculated through propagation of uncertainty from the replicate's standard deviations.

Method Table 5. Spectral settings used for fluorescence turn-on assay.

**Fluorophore**	**λ_{ext} [nm]**	**λ_{em} [nm]**	**$\lambda_{em,max}$ [nm]**
JF_{525}	500 ± 10	540 - 750	558
TMR	530 ± 10	570 - 750	580
MaP555	530 ± 10	570 - 750	580
JF_{585}	560 ± 10	602 - 750	612
CPY	580 ± 10	620 - 800	634
MaP618	580 ± 10	620 - 800	638
JF_{635}	600 ± 10	638 - 800	660
SiR	605 ± 10	652 - 800	670
JF_{656}	610 ± 10	650 - 800	670

Affinity measurement by fluorescence polarisation assays

The dissociation constants K_D of fluorescent xHTL (10 nM) and (d)HaloTag proteins [0 – 100 µM] were determined in activity buffer supplemented with 0.5% (w/v) Bovine Serum Albumin (BSA, Fraktion V, Roth) as previously described.[1] All measurement were performed in black flat bottom low-volume 384 well plates (Greiner, 20 µL) at 37 °C. The fluorescence polarization (FP, **eq. 5**) was measured in technical triplicates on a microplate reader (Spark20M, Tecan). Excitation and emission settings used for various dyes are listed in

Method Table 6 and the gain and G factor were optimized for each dye individually and kept constant over the replicates. Maximum FP values of each dye were determined using covalently labeled HaloTag7 (100 µM) labeled with the respective fluorophore-HTL substrate (20 nM).

$$\mathrm{FP} = \frac{I_{II} - I_{\perp}G}{I_{II} + I_{\perp}G} \qquad (5)$$

FP: fluorescence polarization, I_{II}: fluorescence intensity parallel to the excitation light polarisation, $I_{\perp}$: fluorescence intensity perpendicular to the excitation light polarisation, G: grating factor $I_{II}/ I_{\perp}$.

Data was normalized between 0 - 1 *i.e.* the minimum FP value provided by the free fluorophore and the maximum FP value provided by the fluorophore fully bound (or corresponding covalent labeling). Technical triplicates were averaged obtaining mean and standard deviations (S.D.) and a single-site binding model (**eq. 6**) was fitted to the data to estimate K_D values. Confidence intervals and standard deviations of fitted parameters were estimated with the Monte Carlo[28] method with N = 1000. K_D values are represented with 95% confidence interval (CI 95%). All experiments were performed in at least three individual replicates and all data was used for the fit ($x_n \geq 3$).

$$\mathrm{Y} = \frac{1}{1+\frac{\mathrm{K}_d}{[\mathrm{HT7}]}} \qquad (6)$$

Y: Normalized FP, A: min. FP, B: max. FP, [HT7]: (d)HaloTag7 protein concentration [M], K_D: dissociation constant [M].

Method Table 6. Spectral settings used for fluorescence polarization assays.

Fluorophore	λ_{ext} [nm]	λ_{em} [nm]
TMR	535 ± 12.5	595 ± 17.5
MaP555	535 ± 12.5	595 ± 17.5
CPY	600 ± 10	660 ± 10
SiR/JF_{635}	635 ± 10	655 ± 10

Stopped-Flow kinetic fluorescence polarisation assay

Binding rate constants were determined by measuring fluorescence anisotropy changes over time with a BioLogic SFM-400 stopped flow instrument (BioLogic Science Instruments). 4 µM of HaloTag7 protein was mixed in a 1:1 (v/v) ratio (single-mix sequence) with 1 µM fluorophore-xHTL in activity buffer at 37 °C. Fluorescence intensity and fluorescence anisotropy were measured over 300 s in 0.1 ms intervals. Monochromator wavelengths for excitation and longpass (LP) emission filters are summarized in

Method Table 7.

Method Table 7. Filter-settings used for stopped-flow kinetics fluorescence polarization experiments.

Fluorophore	λ_{ext} [nm]	Emission filter LP [nm]
TMR/MaP555	553	570
JF_{635}	635	655
SiR	650	665

The dead-time estimated by the instrument software (3.7 ms) was subtracted from time points. Background anisotropy corresponding to the free fluorophore was subtracted from the data and at least 8 technical replicates were averaged. Time-dependent anisotropy data was normalized to the highest polarization values (B) and a second order integrated rate equation (**eq. 7**) was fitted to the data yielding k_1 as the binding rate constant (on-rate). Confidence intervals and standard deviations of fitted parameters were estimated with the Monte Carlo[28] method with N = 1000. k_{on} values are represented with 95% confidence interval (CI 95%).

$$\mathrm{A} = 1 + \frac{\frac{-1}{[D]_0}\cdot\left([D]_0\cdot([D]_0-[HT7]_0)\right)\cdot e^{([D]_0-[HT7]_0)\cdot k_1\cdot t}}{[D]_0\cdot e^{([D]_0-[HT7]_0)\cdot k_1\cdot t}-[HT7]_0} \qquad (7)$$

A: fluorescence anisotropy, $[D]_0$: initial dye concentration, $[HT7]_0$: initial (d)HaloTag7 protein concentration, t: time [s], k_1: on-rate [M^{-1} s^{-1}].

Cell biology and microscopy

All cell lines (U2OS, HeLa Kyoto, HEK293 and Cos-7) were maintained in T-25 flasks (Greiner) in high-glucose Dulbecco's Modified Eagle Medium (DMEM GlutaMAX™, phenol-red, Gibco). Growth medium was supplemented with 10% (v/v) fetal calf serum (FCS) and cells were stored in a humidified tissue culture incubator at 37 °C and 5% CO_2. Cells were passaged using phosphate buffered saline (PBS, pH 7.4, Gibco) and TrypLE™ Select Enzyme (1x, phenol-red free, Gibco) every 2-3 days and regularly tested for mycoplasma contamination. Cell titters were determined using a fluidlab R-300 handheld cell counter.

Stable cell-line establishment

Stable cell lines[15] were generated using the Flp-IN T-REx™ system[29] in U2OS cells (ThermoFisher Scientific). Cells were grown in T-25 flasks to 80% confluence, pcDNA5/FRT/TO plasmid encoding the gene of interest (GOI) and pOG44 plasmid were co-transfected (1:10). The transfection medium was replaced 16-18 h after transfection with fresh growth medium and stable cell lines were selected using 50 µg ml^{-1} hygromycin B (ThermoFisher Scientific) 24 h post-transfection for 2 days. Cells with genomic transgene integration were eventually selected for high-expression level using fluorescence activated cell sorting (FACS) on a FACSMelody™ Cell sorter (BD Biosciences) using xHTL staining (500 nM). The different cell lines used in this study are summarized in **Method Table 8**.

Neuron preparation and maintenance

For neuron culture, µ-Slide 8-well glass-bottom plates (ibidi) were coated with poly-L-ornithine (100 µg/mL) for 20 minutes, washed three times with 1x PBS and coated with laminin (1 µg/mL in 1x HBSS) for 1 hour. New born pups (0-1 days, WISTAR rats) were sacrificed and the hippocampi were isolated. After tryptic digest, mechanical dissection using a pipette was used to obtain a homogenous solution. The solution was filtered through a cell strainer (40 µm pore size) and the cell numbers were measured considering live and dead cells using a Countess® II FL Automated Cell Counter (ThermoScientific). 110'000 live cells were seeded per well. The medium was replaced 2 h after seeding with fresh phenol-red free 27 neurobasal medium (NB) supplemented with Penicilin/Streptomycin (Life Technologies), GlutaMAX and B27 was added. Hippocampal neurons were maintained in a humidified incubator at 37° C and 5% CO_2 atmosphere.

rAAV production and neuron transduction

Recombinant AAVs (rAAVs) were generated as described earlier.[30] In brief, plasmids pRV1 (AAV2 Rep and Cap sequences), pH21 (AAV1 Rep and Cap sequences), pFD6 (Adenovirus helper plasmid) and the AAV plasmid containing the recombinant expression cassette flanked by AAV2 packaging signals (ITRs) were transfected via Polyethylenimine 25000 (PEI25000) into HEK293 cells. The cells were harvested 5 days post transfection and lysed using TNT extraction buffer (20 mM Tris pH7.5, 150 mM NaCl, 1% TX-100, 10 mM MgCl2). The cell debris was pelleted and the cell supernatant treated with Benzonase. The rAAVs were purified from the medium and cell supernatant via FPLC using AVB Sepharose columns, which were subsequently concentrated using centrifugal filter devices (Amicon, Merck KGaA, Darmstadt, Germany) with a MWCO of 100 kDa and buffer exchanged to PBS pH 7.3. Hippocampal neurons were transduced with rAVVs after 5 days in culture. ~ 10^9-10^{10} (0.5 µL) rAAV particles were diluted into 10 µL of phenol-red free NB medium and subsequently added to corresponding samples. After 10 - 16 days in vitro (DIV), the neurons were stained and used for microscopy.

Cell fixation and staining

Cells were fixed after PBS wash using prewarmed 4% (v/v) cell-culture grade paraformaldehyde solution (PFA, Electron Microscopy Sciences) and 0.1% (v/v) glutaraldehyde (GA, Electron Microscopy Sciences) in PBS for 30 min at 37 °C. Excess fixative was quenched using 50 mM ammonium chloride and the cells were subsequently washed with PBS three times. Cells were stored in PBS up to 1 week at 4 °C.

Live-cell staining

For live-cell microscopy imaging, 1.0 to 1.5 x 10^5 cells per well were seeded into tissue culture treated CellCarrier 96 wells black plates (PerkinElmer) or CELLview slide 10 wells (Greiner Bio-One) with an optically clear glass-bottom. The medium was changed 14 h after seeding to regular growth medium, supplemented with 0.1 µg/mL doxycycline to induce protein expression in case of U2OS Flp-IN T-REx™ stable cell lines. Transient transfection was performed using Lipofectamine 3000® reagent (ThermoFisher Scientific) according to the manufacturer's protocol. Live-cell staining was performed 22 h after seeding or transfection. Fluorophore-xHTL were applied in imaging medium (DMEM GlutaMAX™, 10% FCS, phenol-red free, Gibco) at 500 nM concentrations for 2 h at 37 °C prior to imaging.

Flow cytometry

Flow cytometry experiment and fluorescence-activated cell sorting (FACS) of fluorescently labeled U2OS cells were performed on a FACSMelody™ Cell sorter (BD Biosciences). First, cells were grown in 6-well cell-culture plates (Nunc™, Thermo Fisher Scientific) until 80% confluency and detached with 1 mL TrypLE™ (Gibco) for 2 min at rt, resuspended in 2 mL PBS w. 2% FCS containing 500 nM fluorophore-(x)HTLs and filtered through cell-strainer caps (Falcon).

Cells were flowed in BD FACSFlow buffer sheath fluid (BD Biosciences) and a 100-µm nozzle tip. Forward and side scatter (FSC and SSC, respectively) were generated by a 647 nm laser which, and in combination with an 670/20 nm emission filter, was used to monitor SiR fluorescence signals. Cytometer analysis was performed by gating target populations in several 2D plots: (i) FSC area versus SSC area; (ii) SSC/FSC height versus SSC/FSC area and (iii) SiR-fluorescence area versus SSC area. 50,000 U2-OS cells showing highest 10% SiR-fluorescence signal were sorted using the purity mode directly into CellCarrier-24 black plates with an optically clear glass-bottom (PerkinElmer) containing 500 µL growth media with Penicillin G (100 U/mL) and Streptomycin (200 µg/mL). Data analysis was performed with FlowJo (Tree Star) software.

Confocal Fluorescence Microscopy

Live-cell confocal fluorescence images were acquired on a Leica DMi8 microscope (Leica Microsystems) equipped with a Leica TCS SP8 X scanhead and a SuperK white light laser and a 405 nm diode laser (Hoechst channel). A HC PL APO 40x/1.10 W motCORR CS2 water objective (i) or a HC PL APO 63x/1.40 Oil CS2 oil immersion objective (ii) were used in combination with hybrid detectors (HyD). Multi-color images were acquired by sequential imaging to avoid spectral bleed-through. All confocal images were recorded in 1024x1024 pixel resolution (12 bit) with a pixel dwell time of 862 ns and a pinhole size of 1 Airy Unit unless otherwise stated. Imaging settings are summarized in **Method Table 9**. Live cells were maintained in a CO_2 (5%) and temperature-controlled (37 °C) chamber (Life Imaging Services).

Pulse-chase experiments were carried out in µ-Slide VI 0.5 Glass Bottom slides (Ibidi) connected to a custom-build perfusion system by addition or replacement of the staining solution (SiR-xHTLs, 500 nM in HBSS, 1x, no Ca^{2+}/Mg^{2+}, phenol red free, Corning) directly under the microscope and recording of 75 consecutive z-stacks (2 channels, each 10 slices of 2 µm step size) with an imaging speed of 2 stacks per minute. The experiment was repeated three times and the fluorescence intensity of individual nuclei was extracted using the Fiji[31] software and normalized to MaP555-BG/SNAP counter-staining.

Image processing and analysis was performed using the Fiji[31] software where brightness and contrast were adjusted. For image representation, sum or maximum projections were created as indicated. 'Hot' lookup tables (LUT) were applied as indicated. From circular ROIs, the cellular brightness (R_{FI}) was measured in H2B-(d)HaloTag7-T2A-mEGFP[15] U2OS cells (n≥150 cells) from 4 fields of view (FOVs) (**eq. 8**) from

- the fluorescence intensities of the dye in the nucleus (FI_{nuc}^{Dye}) and
- the fluorescence intensities in the cytosolic mEGFP channel (FI_{cyto}^{mEGFP}).

$$R_{FI} = \frac{FI_{nuc}^{Dye}}{FI_{cyto}^{mEGFP}} \qquad (\mathbf{8})$$

R_{FI}: Cellular brightness, FI_{nuc}^{Dye}: Nuclear dye fluorescence intensity on H2B-(d)HaloTag7, FI_{cyto}^{mEGFP}: Cytosolic mEGFP fluorescence intensity.

In H2B-HaloTag7 expressing cells co-seeded with cells not expressing any HaloTag fusion, the signal specificity, *i.e.* signal-over-background ratio (S/B), was calculated from (n≥50 cells, 3 FOVs, **eq. 9**)

- the fluorescence intensities of the dye in the nucleus of (d)HaloTag7 expressing cells ($FI_{HaloTag7}^{Dye,nuc}$) and
- the fluorescence intensities of the dye in the nucleus of non-expressing cells ($FI_{emtpy\ cell}^{Dye,nuc}$).
-

$$S/B = \frac{FI_{HaloTag7}^{Dye,nuc}}{FI_{empty\ cell}^{Dye,nuc}} \qquad (\mathbf{9})$$

S/B: Signal-over-background, $FI_{HaloTag7}^{Dye,nuc}$: nuclear dye fluorescence intensity of cells expressing H2B-HaloTag7, $FI_{empty\ cell}^{Dye,nuc}$: nuclear dye fluorescence intensity of cells expressing no HaloTag7 fusion.

Wide-field Fluorescence Microscopy

Widefield fluorescence imaging was performed on a DMi8 widefield microscope (Leica) equipped with a HC PL APO 40x/1.10 W motCORR CS2 water objective (i) or a HC PL APO 20x/0.8 (dry) objective (iii). SiR fluorescence was excited at 635/18 nm and detected using a 700/75 nm filter. The eGFP intensity was recorded using a 474/27 nm excitation and 525/50 nm emission filter (2x2 pixel binning). Live-cells were maintained in a CO_2 (5%) and temperature-controlled (37 °C) chamber (PeCon). Imaging settings are summarized in **Method Table 9**.

Live-cell labeling kinetics were measured in µ-Slide VI 0.5 Glass Bottom slides (Ibidi). SiR-xHTLs (25 nM in HBSS, 1x, no Ca^{2+}/Mg^{2+}, phenol red free, Corning) were flushed in the imaging chambers directly under the microscope while recording Z-stacks (2 channels, 20 stacks, 2 stacks/minute). Signal brightness (R_{FI}) was extracted as explained before (**eq. 9**) from n≥15 cells. The data was corrected by the background fluorescence (t = 0) and the maximal final intensity (= 1) for each individual dye and plotted against the imaging time. An exponential association function (**eq. 10**) was fitted to the data to determine the rate-constants k and extract the half-labeling time $\tau_{1/2}^{kin}$ (**eq. 11**).

$$y = A \cdot (1 - e^{-kx}) \quad \textbf{(10)}$$

y: normalized fluorescence intensity, x: time [min], A: amplitude, k: rate-constant [min^{-1}].

$$\tau_{1/2}^{kin} = \frac{ln(2)}{k} \quad \textbf{(11)}$$

$\tau_{1/2}^{kin}$: half-labeling time [min], k: rate-constant [min^{-1}].

PAINT Imaging

PAINT experiments were performed using fixed U2OS cells on µ-Slide VI 0.5 Glass Bottom (Ibidi) at a confluence of 80%. For DNA-PAINT experiments cells were permeabilized using 3% IgG-free BSA and Saponin in PBS for 30 min at rt. Afterwards, primary vimentin antibodies (recombinant monoclonal anti-Vimentin, EPR3776, ab92547, abcam) were diluted 1:200 in PBS and added to the chamber. After incubation for 2 h at rt, excess primary antibodies were removed by three washing steps with PBS followed by incubation for 2 h at rt with custom DNA-docking strand labeled secondary antibodies diluted 1:100 in PBS (AffiniPure donkey-anti rabbit IgG (H+L, 711-005-152), 5'-TTCATTACTTCT -3', 1,3 mg mL^{-1}). After removing excess secondary antibodies with three washing steps using PBS, cells were post-fixed with 4% PFA for 10 min at rt and finally washed thrice with PBS. Prior to DNA-PAINT measurements, P1-ATTO655 imager strands (P1 5'-AGAAGTAATG-ATTO655-3') were diluted in imaging buffer (500 mM NaCl in PBS, pH 8.3) to a concentration of 1 nM and added to the chambers.
For sample drift correction and image registration fiducial gold markers (125 nm gold beads, Nanopartz, USA) were used. They were diluted 1:30 in PBS, sonicated for 10 min and 100 µL of the solutions were added to the microscopy chambers. After settlement of the gold beads (5 min), the samples were washed thrice with PBS.

For single-colour HT-PAINT and the determination of the single-molecule binding kinetics, fluorescent xHTLs were diluted in PBS to a final concentration of 1 nM and added to the samples. For exchange 2-color PAINT[32] the xHTLs were diluted to 3-5 nM in PBS and injected to the sample using a microfluidic system (Bruker) at a flow-rate of 600 µL/min, sequentially, including a PBS wash step at a flow-rate of 600 µL/min in between imaging cycles. For DNA-PAINT measurements, P1-ATTO655 imager strands were diluted in DNA-PAINT imaging buffer and added to the sample at a concentration of 1 nM.

Single-molecule fluorescence microscopy was performed at the commercial N-STORM super-resolution microsystem (Nikon, Japan). The optical setup was equipped with an oil immersion objective (objective Apo, 100x, NA 1.49) and an EMCCD camera (DU-897U-CS0-#BV, Andor Technology, Ireland). SiR and JF_{635}-xHTLs or DNA-PAINT imager strands were excited with a collimated 647 nm laser beam at an intensity of 1.1 kW cm^{-2} (measured at the objective) at highly inclined and laminated optical sheet mode (HILO). PAINT data was reconstructed from 30.000 – 40.000 frames recorded with an integration time of 100 to 150 ms, depending on the experiment. Resulting total imaging times ranged between 66 to 75 min. Following parameters were used for image acquisition: camera integration time of 100-150 ms in active frame transfer mode with an EMCCD camera gain of 200, a pre amp gain of 1 and at an effective pixel size of 158 nm. Software tools NIS Elements (Nikon, Japan), LCControl (Agilent, USA), and Micro-Manager 1.4.22[33] were used for optical setup control and image acquisition. For 2-color single-molecule microscopy, a microfluidic system (Bruker, USA) was used for automated exchange of fluorescent ligands.

PAINT-Image processing and determination of single-molecule binding kinetics

Localization of single molecules and the reconstruction of super-resolution images were performed with the modular software package Picasso[34]. Single-molecule spots were identified in individual frames using the integrated Gaussian maximum likelihood estimation using the following parameter: Min. net. gradient of 10 000-15 000, a baseline of 205, a sensitivity of 4.78 and a quantum efficiency of 0.95. The images were subsequently corrected for lateral drift using the position of fiducial marker via an integrated redundant cross-correlation algorithm. Single-molecule localizations were then filtered based on SiR, JF_{635} and ATTO655 single-molecule footprints (PSF symmetry $0.6 < FWHM(x)/FWHM(y) < 1.3$) and intensity thresholds. Binding events from the same origin that were spanning over multiple consecutive frames were spatiotemporally linked within a radius of five times the nearest-neighbor based analysis (NeNa[33]) localization precision and allowing a maximum dark time of 5 consecutive frames.

The spatial resolution was calculated by decorrelation analysis[35] and Fourier Ring Correlation (FRC)[36] analysis as reported previously. The average binding times (bright time, τ_b) and off-rate constant (k_{off}) of the fluorophore-xHTLs were determined from reconstructed super-resolution images. In brief, spatiotemporally linked single binding events (n > 300) were picked manually from reconstructed super-resolution images using the Picasso software. The relative frequency of τ_b was then plotted in Origin software 2019[37] and fitted with a gaussian distribution to determine the average binding time. k_{off} was then calculated as $1/\tau_b$. For PAINT imaging, 'Hot' LUT were applied for data representation.

MINFLUX Microscopy

For MINFLUX experiment, U2OS vimentin-HaloTag7[38] cells were seeded on glass coverslips (precision cover glasses, thickness No. 1.5H, tol. ± 5 µm, 18 mm Ø, Martinsried), grown until a confluence of 60% was reached and chemically fixed as described above. Cells were permeabilized using 0.1% Triton-X100 for 10 min at rt followed by 3x PBS-wash and afterwards incubated with a primary mouse anti-vimentin antibody (Sigma Aldrich, cat. V6389,1:500, 1 h, rt) and a secondary goat anti-mouse, AlexaFluor488-labeled antibody Thermo Fisher, cat. A32723,1:1000, 1 h, rt) with 5 washing steps with PBS w. 1% (w/v) BSA in between and after. Next, the cells were incubated with fiducial markers (5 min, rt, NanoParz, cat. A12-40-980-CTAB-DIH-1-25) washed three times with PBS w. 1% (w/v) BSA and stained with 2 nM SiR-T5 in PBS w. 1% (w/v) BSA.

MINFLUX imaging was performed on an Abberior 3D MINFLUX (Abberior Instruments GmbH, Göttingen, Germany) built on a motorized inverted microscope IX83 (Olympus, Tokyo, Japan) and equipped with 640 nm and 561 nm MINFLUX laser lines, 488 nm and 405 nm confocal lines. Detection was performed with APDs in the spectral windows 720-685 nm and 650-685 nm. Images were acquired using the default 2D imaging sequence, with an L in the last iteration step of 40 nm, and photon limit of 200 photons. Pinhole was set to 0.67 A.U. and excitation power at the periscope for the first iteration was ~ 35 µW.

Five filters were applied in the post-processing step to avoid false single molecule emission events. To exclude detections originated from transient background, we set the maximum thresholds of 1.2 for the center frequency ratio (CFR) test and 100 kHz for the detected fluorescence rate.[39] Localizations from the same emission trace, *i.e.* with same TiD, further than three standard deviations with respect to the mean trace position were considered outliers and excluded from the trace. Only the traces containing at least 3 localizations within the first 180 minutes of the measurement were considered to calculate the localization precision. The experimental localization precision was estimated by co-aligning the mean values of all localizations obtained from individual emission traces fitted with a Gaussian function to estimate the overall standard deviation.[40] For MINFLUX imaging, 'Hot' LUT were applied for data representation.

Live-cell STED Microscopy

Live cells STED nanoscopy was performed using an Abberior STED Expert Line 595/775/RESOLFT QUAD scanning microscope (excitation lines: 355 nm, 405 nm, 485 nm, 561 nm, 640 nm; STED lines: 595 nm and 775 nm; RESOLFT lines: 405 nm, 488 nm) equipped with a UPlanSApo 100x/1.4 oil immersion objective lens (Abberior Instruments, iv), or an Abberior STED Infinite line 660/775 QUAD scanning microscope (excitation lines: 520 nm, 561 nm, 640 nm, multiphoton; STED lines: 655 nm and 775 nm) equipped with a 60x/1.42 UPLXAPO60XO oil immersion objective lens (Abberior Instruments, v, both Abberior Instruments GmbH). Detection was performed with avalanche photodiodes (APD) and spectral detection. Fluorophores, microscope and imaging settings are summarized in **Method Table 10**. For STED imaging, 'Hot' LUT were applied for data representation.

STED performance was assessed by measuring the Full Width at Half Maximum (FWHM) of single intermediate filament fibers revealed by Vimentin-HaloTag7. STED images were acquired in an 8x8 µm window with a pixel dwell time of 10 µs and a pixel size of 20 nm with 2 average line scans. Image analysis was performed with *ImageJ*[31] by extracting fluorescence intensity profiles perpendicular to vimentin filaments. Mean filament diameters were calculated from at least ten individual fibrils and from at least three individual images (n ≥ 3) by fitting a gaussian function (**eq. 12**), yielding FWHM from **eq. 13**.

$$y = y_0 + \frac{A}{\omega\sqrt{\frac{\pi}{2}}} e^{-2\frac{(x-x_c)^2}{\omega^2}} \quad (12)$$

y: normalized fluorescence intensity, x: x-coordinate [µm], y_0: offset, A: area, ω: width, x_c: center.

$$FWHM = \omega\sqrt{2\ln(2)} \quad (13)$$

FWHM: Full Width at Half Maximum, ω: Gauss width.

Photobleaching by multi-frame STED acquisition was recorded in an 8x8 µm window (detailed settings are summarized in **Method Table 10**) and exemplary magnification of single mitochondrial tubules are shown. Bleaching curves were generated by extracting the mean pixel values over the imaging series using *ImageJ.* The image borders were excluded from the evaluation to avoid imaging artefacts. The data was background corrected and normalized to the first frame intensity. Mean intensities from at least three individual experiments (n ≥ 3) were plotted against the frame number and individually fitted to a mono-exponential decay equation (**eq. 14**) to derive the frame number before reaching half the initial intensity (frame number at half-intensity $\tau_{1/2}$). Mean values and standard deviation of $\tau_{1/2}$ are presented.

$$I = I_0 \cdot e^{\frac{-x}{\tau_{1/2}}} \quad (14)$$

I: normalized fluorescence intensity, I_0: Initial intensity, x: frame number, $\tau_{1/2}$: frame number at half-intensity.

3D-STED images were generated by recording consecutive x-y-frames with 80% axial STED (z-direction).

Statistical analysis and reproducibility.
Propagation of uncertainty for variables from products and quotients was calculated using **eq. 15**.

$$\sigma = |C|\sqrt{\left(\frac{\sigma_A}{A}\right)^2 + \left(\frac{\sigma_B}{B}\right)^2} \quad (15)$$

σ: standard deviation, A, B: variables, σ_A, σ_B: standard deviation of variables A and B, $C = \bar{A} * \bar{B}\ or\ C = \frac{\bar{A}}{\bar{B}}$.

Statistical significance of a sample set over a reference set (n ≥ 3) was probed by performing two-tailed t-test (spectroscopic characterization of Φ, ε, and F/F_0 and confocal as well as STED bleaching $\tau_{1/2}$). A Welch correction for larger sample sizes was used (for cellular R_{FI} and S/B, n ≥ 50). Statistical analysis were performed with the *OriginLab*[37] software. p-values > 0.05 were considered as non-significant (n.s.) and p ≤ 0.0025 as significant as indicated in the respective figure captions.

Method Table 8. Plasmids, stable U2OS FlpIn TRex cell lines (SCL) and rAAVs derived thereof.

Name	Addgene#	Plasmid	Gene	SCL	rAAV
pET51b(+)_HaloTag7 [a]	167266	pET51b(+)	HaloTag7	-	-
pET51b(+)_dHaloTag7 [a]	167267	pET51b(+)	dHaloTag7 = HaloTag7-D106A	-	-
pCDNA5/FRT/TO_ NLS-HaloTag7-SNAP-tag	-	pCDNA5/FRT/TO	NLS-HaloTag7-SNAP-tag	Yes	-
pCDNA5/FRT/TO_H2B-HaloTag7_T2A_mEGFP [c]	187070	pCDNA5/FRT/TO	H2B-HaloTag7 and mEGFP	Yes	-
pCDNA5/FRT/TO_H2B-dHaloTag7_T2A_mEGFP	187071	pCDNA5/FRT/TO	H2B-dHaloTag7 and mEGFP	Yes	-
pCDNA5/FRT/TO_TOM20-HaloTag7_T2A_mEGFP [b]	187072	pCDNA5/FRT/TO	TOM20-HaloTag7 and mEGFP	Yes	-
pCDNA5/FRT/TO_TOM20-dHaloTag7_T2A_mEGFP	187073	pCDNA5/FRT/TO	TOM20-dHaloTag7 and mEGFP	Yes	-
pCDNA5/FRT/TO_LifeAct-HaloTag7_T2A_mEGFP	-	pCDNA5/FRT/TO	LifeAct-HaloTag7 and mEGFP	-	-
pCDNA5/FRT/TO_Vimentin-HaloTag7_T2A_mEGFP [c]	187074	pCDNA5/FRT/TO	Vimentin-HaloTag7 and mEGFP	Yes	-
pCDNA5/FRT/TO_Vimentin-dHaloTag7_T2A_mEGFP	187075	pCDNA5/FRT/TO	Vimentin-dHaloTag7 and mEGFP	Yes	-
pCDNA5/FRT/TO_H2B-HaloTag7 [c]	169329	pCDNA5/FRT/TO	H2B-HaloTag7	Yes	-
pCDNA5/FRT/TO_H2B-dHaloTag7	-	pCDNA5/FRT/TO	H2B-dHaloTag7	Yes	-
pCDNA5/FRT/TO_TOM20-HaloTag7 [c]	169330	pCDNA5/FRT/TO	TOM20-HaloTag7	Yes	-
pCDNA5/FRT/TO_TOM20-dHaloTag7	-	pCDNA5/FRT/TO	TOM20-dHaloTag7	Yes	-
pCDNA5/FRT/TO_NES-HaloTag7 [c]	-	pCDNA5/FRT/TO	NES-HaloTag7	-	-
pCDNA5/FRT/TO_Ig-κ-HaloTag7-PDGFR [c]	-	pCDNA5/FRT/TO	Ig-κ-HaloTag7-PDGFR	-	-
pCDNA5/FRT/TO_Ig-κ-dHaloTag7-PDGFR	-	pCDNA5/FRT/TO	Ig-κ-dHaloTag7-PDGFR	-	-
pCDNA5/FRT/TO_CalR-HaloTag7-KDEL [c]	-	pCDNA5/FRT/TO	CalR-HaloTag7-KDEL	-	-
pCDNA5/FRT/TO_CalR-dHaloTag7-KDEL	-	pCDNA5/FRT/TO	CalR-dHaloTag7-KDEL	Yes	-
pCDNA5/FRT/TO_LamP1-HaloTag7 [c]	-	pCDNA5/FRT/TO	LamP1-HaloTag7	-	-
pCDNA5/FRT/TO_TOM20-dHaloTag7_T2A_Vimentin-HaloTag7	-	pCDNA5/FRT/TO	TOM20-dHaloTag7 and Vimentin-HaloTag7	Yes	-
pCDNA5/FRT/TO_TOM20-dHaloTag7_T2A_Lifeact-HaloTag7	187076	pCDNA5/FRT/TO	TOM20-dHaloTag7 and Lifeact-HaloTag7	-	-
pCDNA5/FRT/TO_TOM20-dHaloTag7_T2A_Cox8-HaloTag7	187077	pCDNA5/FRT/TO	TOM20-dHaloTag7 and Cox8-HaloTag7	Yes	-
pCDNA5/FRT/TO_TOM20-dHaloTag7_T2A_LamP1-HaloTag7	187078	pCDNA5/FRT/TO	TOM20-dHaloTag7 and LamP1-HaloTag7	Yes	-
pCDNA5/FRT/TO_TOM20-dHaloTag7_T2A_CalR-HaloTag7-KDEL	187079	pCDNA5/FRT/TO	TOM20-dHaloTag7 and CalR-HaloTag7-KDEL	Yes	-
hSyn-Cox8A-HaloTag7-Pro30-SNAP-WPRE-SV40	-	hSyn	Cox8A-HaloTag7-SNAP	-	Yes
hSyn-Lyn11-HaloTag7-mEGFP-WPRE-SV40	-	hSyn	Lyn11-HaloTag7-mEGFP	-	Yes
CRISPR Vimentin-HaloTag [d]	-	-	Vimentin-HaloTag7	/	/

Plasmid/cell-line published in [a] Wilhelm, Kühn *et al.* (2021)[1], [b] Frei *et al.* (2019)[14], [c] Frei *et al.* (2021)[15] and [d] Butkevich *et al.* (2018)[41].
SCL = Stable cell lines U2OS Flp-In TREx.

Method Table 9. Conventional fluorescence microscopy data acquisition parameters.

Fig./Vid.	Label	Ligand	Set-up	Obj.	Excitation [nm] (%)	Emissinon [nm]	Comment
4A	H2B-HaloTag7	JF_{525}-T5	A	i	525 (5)	535 - 600	Sum projections
		MaP555-T5			550 (16)	560 - 610	
		JF_{585}-S5			585 (3)	595 - 685	
		MaP618-S5			620 (10)	630 - 790	
		JF_{635}-S5			635 (3)	645 - 780	
		SiR700-S5			670 (10)	680 - 795	
4B/ Vid.S1	HaloTag7-SNAP-tag^{NLS}	SiR-S5	A	i	640 (4)	650 - 690	Ratiometric and sum projection
		BG-MaP555			550 (4)	560 - 620	
4E	Hoechst	/	A	i	405 (2)	432 - 500	
	H2B-HaloTag7	SiR-T5			633 (3)	650 - 790	
	MaP555-CA	MaP555-CA			550 (10)	560 - 600	
6D	H2B-HaloTag7<br>H2B-dHaloTag7	MaP555-Hy5	A	i	550 (16)	560 - 610	Sum projections
		MaP618-Hy4			620 (10)	630 - 790	
		SiR-Hy4			635 (3)	645 - 780	
7A	H2B-HaloTag7	SiR700-S5	A	i	680 (4)	680 - 795	
	TOM20-dHaloTag7	CPY-Hy4			600 (3)	610 - 650	
7B	H2B-dHaloTag7	MaP555-Hy5	A	i	550 (3)	560 - 600	Sum projections<br>Seq. imaging
	TOM20-HaloTag7	SiR-S5			590 (12)	600 - 635	
	LamP1-SNAP^{f}-tag	JF_{585}-BG			635 (13)	645 - 680	
	Actin	SiR700			690 (12)	720 - 795	
S6	H2B-HaloTag7	TMR-S5/T5/CA	A	i	550 (3)	560 - 600	Sum projections
S7A	NES-HaloTag7	SiR-T5	A	i	633 (2)	650 - 790	Sum projections<br>2 Lines average
	IgK-HaloTag7-PDGFR			ii	633 (10)		
	CalR-HaloTag7-KDEL			i	633 (3)		
	LamP1-HaloTag7			i	633 (10)		
	TOM20-HaloTag7			ii	633 (3)		
	Vimentin-HaloTag7			i	633 (3)		
	Hoechst			i	405 (2)	432 - 500	
S7B	H2B-HaloTag7-T2A-mEGFP	SiR-T5	A	i	633 (5)	650 - 790	Ratiometric and sum projection
		mEGFP			488 (5)	500 - 540	
S7C	Lyn11/Cox8A-HaloTag7	SiR-S5	C	iv	640 (1)	650 - 757	2 Lines accu.
S8	H2B-HaloTag7-T2A-mEGFP	SiR-S5	A	i	633 (3)	650 - 790	Ratiometric and sum projection
		mEGFP			488 (50)	500 - 540	
S9A	H2B-HaloTag7	SiR-T5	A	i	633 (3)	650 - 790	Confocal plane
			B	i	635 (20)	663 - 738	z-stack
S9B	H2B-HaloTag7	SiR-T5	B	iii	635 (20)	663 - 738	sum projection
S16	H2B-dHaloTag7	JF_{525}-Hy5	A	i	530 (4)	540 - 600	Sum projections
		MaP555-Hy5			550 (4)	560 - 600	
		JF_{585}-Hy4			585 (3)	595 - 685	
		MaP618-Hy4			620 (10)	630 - 790	
		JF_{635}-Hy4			635 (1)	645 - 780	
		SiR700-Hy4			670 (10)	680 - 795	
S17	IgK-dHaloTag7-PDGFR	MaP555-Hy5	A	i	550 (2)	560 -600	Sum projections
	H2B-HaloTag7	SiR-S5			633 (2)	650 - 795	
	LifeAct-HaloTag7	SiR-S5			633 (2)	650 - 795	
	TOM20-dHaloTag7	MaP555-Hy5			550 (1)	560 - 620	

Microscopes: **A** SP8-FALCON (confocal), **B** DMi8 (widefield), **C** Abberior STED Expert line (confocal).
Objectives: (i) HC PL APO 40x/1.10 W motCORR CS2 water objective, (ii) HC PL APO 63x/1.40 Oil CS2 oil immersion objective, (iii) HC PL APO 20x/0.8 (dry) objective, (iv) UPlanSApo 100x/1.4 oil immersion objective lens.

Method Table 10. STED microscopy data acquisition parameters.

Fig./Vid.	Label	Ligand	Set-up	Excitation [nm] (%)	STED [nm] (%)	Pixel dwell time [µs]	Pixel size [nm]	Size [µm]	Emission [nm]	Comment
5C	TOM20-HaloTag7	JF$_{635}$-S5<br>JF$_{635}$-CA	A	640 (4)	775 (20)	10	30	10 x 10	650 - 757	2 Lines accu., 4 frame/min
5F	Lyn11-HaloTag7	SiR-S5	A	640 (1)	775 (20)	15	80 (x-y), 20 (z)	40 x 40 x 0.8	650 - 757	2 Lines accu., 40 z-frames, 90% 3D-STED
7D	TOM20-dHaloTag7<br>LamP1-HaloTag7	CPY-Hy4<br>SiR-S5	B	561 (40)<br>640 (2)	660 (20)<br>775 (20)	10	30	10 x 10	571 - 630<br>650 - 757	2 Lines accu.. 2 frames/min
7F/ 5	TOM20-dHaloTag7<br>Cox8A-HaloTag7	CPY-Hy4<br>SiR-S5	B	561 (40)<br>640 (2)	660 (20)<br>775 (20)	10	20	2.4 x 3.2	575 - 630<br>670 - 757	40 x 50 nm z-stacks, 3 Lines accu. 100% 3D-STED
S12A	Vim-HaloTag7	SiR-T5<br>SiR-Halo	A	640 (2)	775 (15)	10	20	8 x 8	650 - 757	2 Lines accu., STED & Conf.
S12B	Vim-HaloTag7	MaP555-T5	B	561 (10)	660 (10)	15	30	10 x 10	571 -650	2 Lines accu., STED & Conf.
		MaP618-S5<br>JF$_{635}$-S5<br>SiR-S5	A	640 (6)<br>640 (6)<br>640 (2)	775 (15)	15	30	10 x 10	571 -693	
S12C	TOM20-HaloTag7	MaP555-T5<br>MaP555-CA<br>JF$_{585}$-CA	B	561 (2)	660 (30)	15	30	15 x 15	650	3 Lines accu., 0.2 frames/min
S12D	TOM20-HaloTag7	MaP618-S5<br>MaP618-CA	A	640 (6)	775 (20)	15	30	10 x 10	650 - 757	2 Lines accu., 1 frame/min
S12E	TOM20-HaloTag7	SiR-S5<br>SiR-Halo	A	640 (4)	775 (20)	10	30	10 x 10	650 - 757	2 Lines accu., 4 frames/min
S12F	TOM20-HaloTag7	SiR-T5	A	640 (8)	775 (40)	15	30	10 x 10	650 - 757	2 Lines accu.
S12G	Lyn11-HaloTag7	SiR-S5	A	640 (1)	775 (20)	15	30	10 x 10	650 - 757	2 Lines accu.
S13	Cox8A-HaloTag7	SiR-S7/-Halo	A	640 (2)	775 (20)	15	30	10 x 10	650 - 757	2 Lines accu.
S18C/ 3	TOM20-dHaloTag7	MaP555-Hy5	B	561 (2)	660 (30)	15	30	15 x 15	650	3 Lines accu.,0.2 frames/min
		MaP618-Hy4<br>JF$_{635}$-Hy4<br>SiR-Hy4	A	640 (6)<br>640 (4)<br>640 (4)	775 (20)<br>775 (20)<br>775 (20)	15<br>10<br>10	30<br>30<br>30	10 x 10<br>10 x 10<br>10 x 10	650 - 757<br>650 - 757<br>650 - 757	2 Lines accu., 1 frames/min<br>2 Lines accu., 4 frames/min<br>2 Lines accu., 4 frames/min
S19B	TOM20-dHaloTag7<br>LamP1-HaloTag7	MaP555-Hy5<br>SiR-S5	B	561 (4)<br>640 (2)	775 (15)<br>775 (20)	10<br>10	100/40	80 x 80<br>10 x 10	571 - 630<br>650 – 757	3 Lines accu., Conf (overview) & STED (zoom)
S19C						Sees Fig. 7D				
S19D	TOM20-dHaloTag7<br>LamP1-HaloTag7	MaP618-Hy4<br>SiR-S5	A	561 (40)<br>640 (4)	775 (20)<br>775 (10)	10	20	2.4 x 3.2	575 - 630<br>670 – 757	40 x 50 nm z-stacks, 3 Line accu.. 100% 3D-STED
Vid. 4	TOM20-dHaloTag7	SiR-S5	A	640 (2)	775 (10)	5	20	6 x 6	670 – 757	~1 frame/s

Microscopes: **A** Abberior STED Expert line with 660/775 depletion lasers, **B** Abberior STED Expert Line with 595/775 depletion lasers.

Supplementary Figures

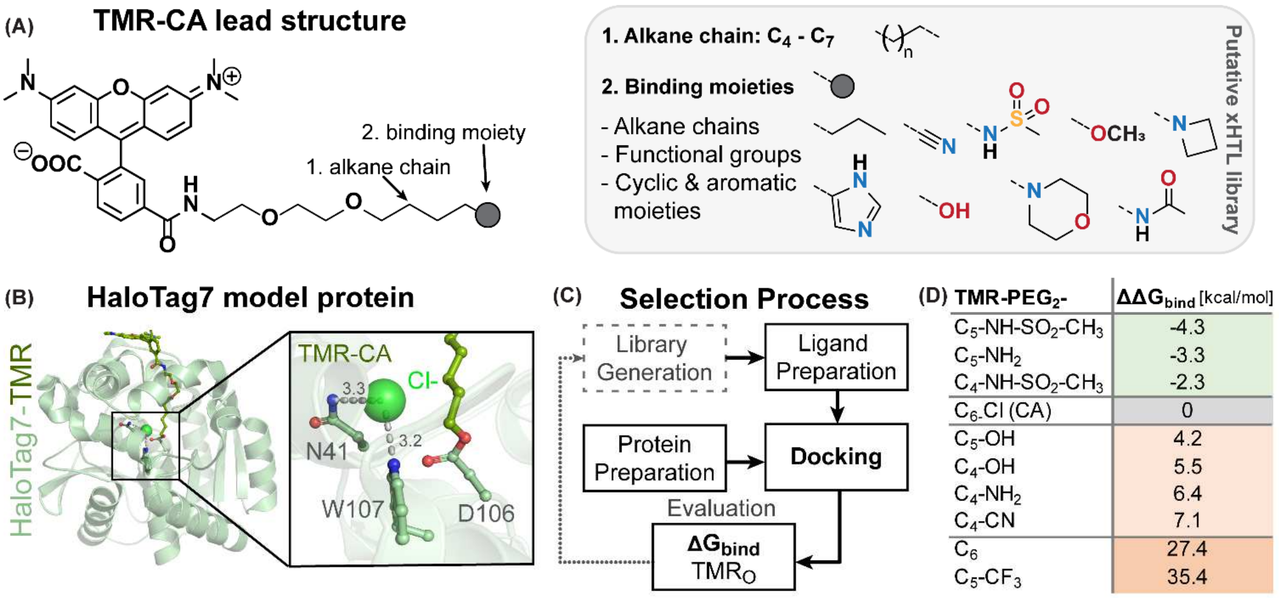

Figure S. 1 | Computational screening of xHTLs candidates.
(A) TMR ligand chemical structure, which was used as lead structure for dockings. Structural elements that were altered for ligand design are represented in the lower insert, namely the terminal alkane chain length (1) and binding moiety (2) generating 2000 xHTLs candidates. (B) Crystal structure of HaloTag7-TMR (PDB ID: 6Y7A)[1] with zoom onto the active site residues. HaloTag7 is represented as cartoon while the alkane-PEG-TMR and important residues are represented as sticks. Distances are indicated in Å. HaloTag7-TMR was used as a target for docking tentative xHTLs. (C) Computational screening process. ΔG_{bind} predicted from docking was calculated for TMR ligands in the open form (TMR_O) and evaluated in comparison to redocking of TMR-HTL such that $\Delta\Delta G = {}^{TMR\text{-}HTL}\Delta G - {}^{candidate}\Delta G$. F. Table summarizing the $\Delta\Delta G_{bind}$ for 9 exemplary xHTL candidates. $\Delta\Delta G_{bind}$ from induced-fit docking ranged from 35.4 (worst binding) to -4.3 kcal/mol (best binding). Best $\Delta\Delta G_{bind}$ were obtained for C4- or C5-aliphatic linkers terminating with hydrogen bond donor moieties such as amines or sulfonamides ($\Delta\Delta G_{bind}$ -4.3 to -2.3 kcal/mol). While primary amines were predicted as good binders ($\Delta\Delta G_{bind}$ = -3.3 kcal/mol), they were not further considered because of the literature-reported low cell permeability and metabolic instability of primary amines.[42] Fluorinated ligands such as trifluorosulfonamides, not initially present in virtual screening, were later considered as they are reported to improve cell permeability.[43]

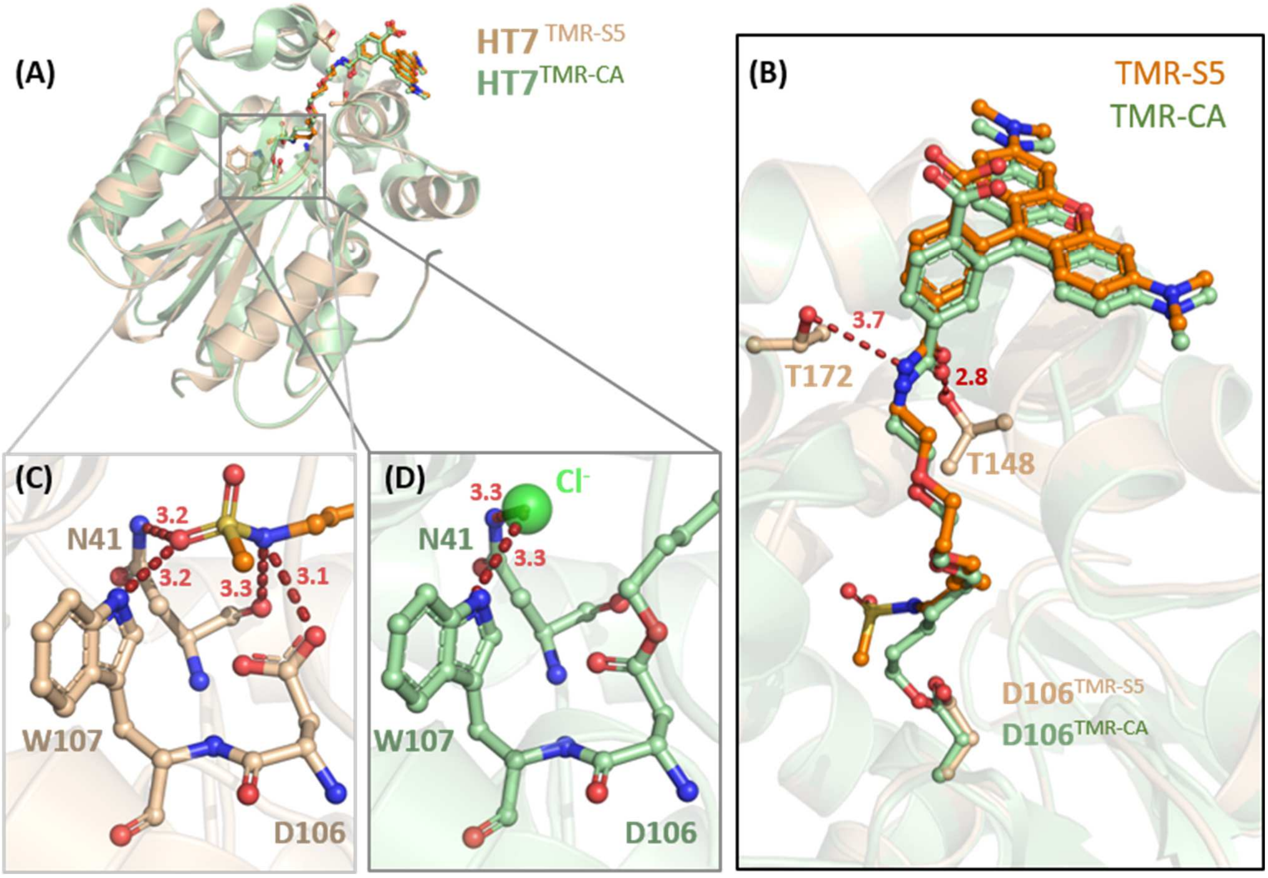

Figure S. 2 | Structural analysis of the TMR-S5/HaloTag7 complex.

(A) Structural comparison between the TMR-S5/HaloTag7 complex (PDB ID: 7ZJ0, 1.5 Å resolution) and TMR-CA/HaloTag7 covalent complex (PDB ID: 6Y7A, 1.4 Å resolution)[1]. Tertiary structures are represented as cartoons. The ligands and relevant residues are represented as sticks. (B) Magnification on the TMR-ligands binding sites. Distances in Å. The TMR moieties are located at the protein's surface while the alkane chains are buried in the protein hydrophobic tunnel as previously described.[1] (C) Magnification on the S5 binding site. Polar interactions between S5 and HaloTag7 residues: TMR-S5 amide moiety interacts with D106 while the sulfonamide group occupies the chloride binding pocket between N41 and W107. (D) Magnification on the covalent bond between TMR-CA and the HaloTag7 protein. The TMR-labeled HaloTag7 features a chloride ion (green sphere) inside the active center where the sulfonamide is located in the S5 complexed structure.

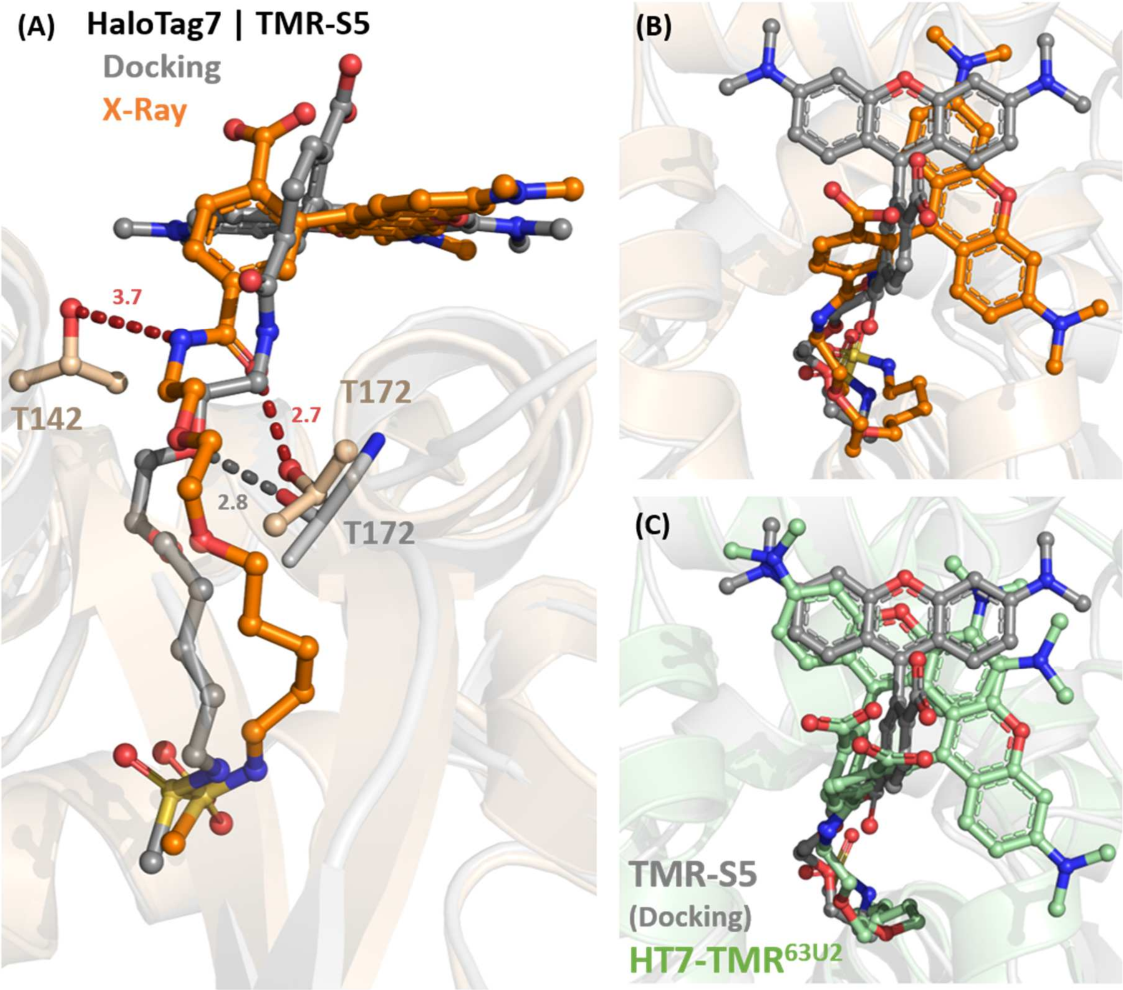

Figure S. 3 | Structural comparison of the TMR-S5 docking results with the TMR-S5/HaloTag7 X-ray structure.
(A) Structural comparison between the TMR-S5 alkane chain orientation in the hydrophobic channel of HaloTag7. X-Ray structure PDB ID: 7ZJ0 at 1.5 Å resolution. Tertiary structures are represented as cartoons. The ligands and relevant residues are represented as sticks. Putative hydrogen bonds with T142/172 presented as dashed lines, distances in Å. The PEG-alkane chain accommodates different conformations to reach the active site in the docking and X-ray structures. B. Magnification on the TMR binding sites of TMR-S5/HaloTag7 complexes from docking or X-ray structures. The TMR moieties are both located at the protein's surface but reveal major difference in their positioning. C. Comparison of the TMR binding sites of TMR-S5/HaloTag7 docking structure and HaloTag7 structure covalently labeled with TMR-CA (PDB ID: 63U2 at 1.8 Å resolution).[44]

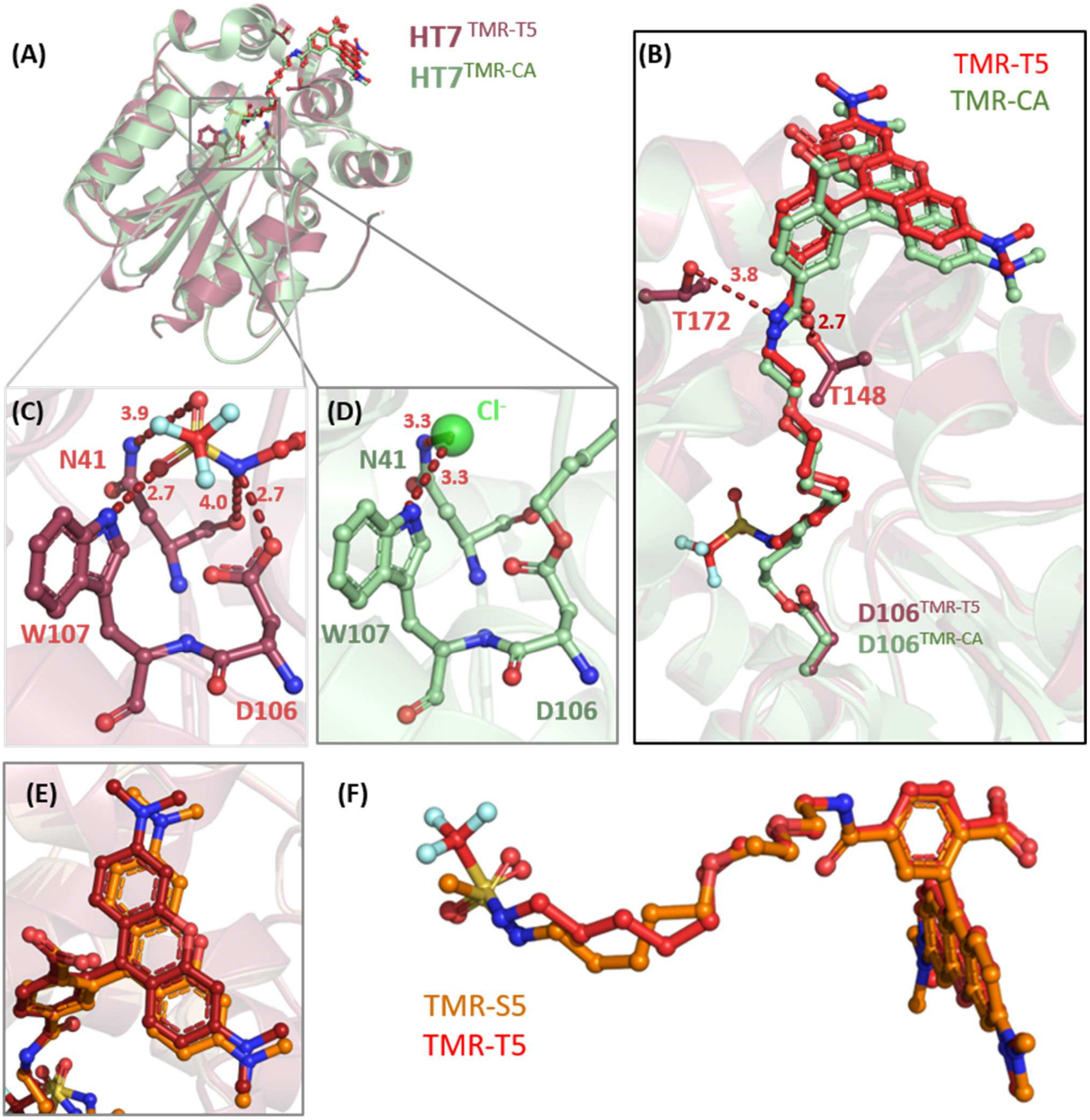

Figure S. 4| Structural analysis of the TMR-T5/HaloTag7 complex.

(A) Structural comparison between the TMR-T5/HaloTag7 complex (PDB ID: 7ZJY, 1.5 Å resolution) and TMR-CA/HaloTag7 covalent complex (PDB ID: 6Y7A, 1.4 Å resolution)[1]. Tertiary structures are represented as cartoons. The ligands and relevant residues are represented as sticks. (B) Magnification on the TMR-ligands binding sites. Distances in Å. The TMR moieties are located at the protein's surface while the alkane chains are buried in the protein hydrophobic tunnel as previously described.[1] (C) Magnification on the T5 binding site. Polar interactions between T5 and HaloTag7 residues: TMR-T5 amide moiety interacts with D106 while the sulfonamide group occupies the chloride binding pocket between N41 and W107. (D) Magnification on the covalent bond between TMR-CA and the HaloTag7 protein. The TMR-labeled HaloTag7 features a chloride ion (green sphere) inside the active center where the sulfonamide is located in the T5 complexed structure. (E) Structural comparison of the S5/T5 TMR position on the surface of HaloTag7 (F) Structural comparison of the S5/T5 conformations found in the respective HaloTag7 crystal structures.

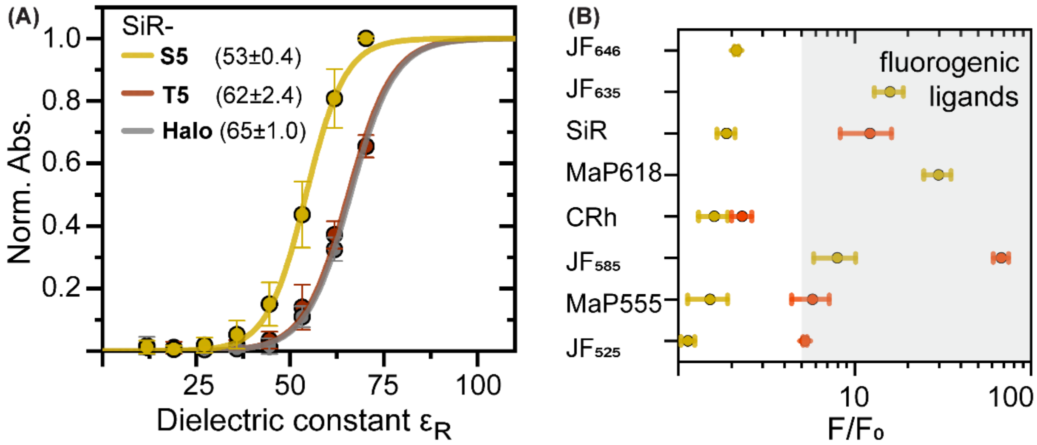

Figure S. 5 | Spectral and fluorogenic property tuning of xHTLs.
(A) SiR-(x)HTL water-dioxane titration curves. 3-Carboxy rhodamines transition from their spirocyclic (non-fluorescent) to the zwitterionic (fluorescent) state with increasing dielectric constant ε_R. By measuring the absorbance at 650 nm at varying water and 1,4-dioxane ratios, we determined the D_{50} value of the probes, which is defined as the ε_R at half-maximal absorbance. The D_{50} value of a fluorophore reflects its propensity to exist in the spirocyclic form. Sigmoidal fit to averaged (n=3) and normalized (to max. absorbance from SiR-S5) data yielded the D_{50}-values (in brackets ± standard deviation), demonstrating that SiR-S5 (53.0 ± 0.4) has a lower propensity to exist in the spirocyclic form than SiR-T5 (62.3 ± 2.4).
(B) xHTL fluorescence intensity increase (F/F_0) upon HaloTag7 binding of fluorescent probes of S5 (yellow) and T5 (red). Mean values (n>3) and standard deviation presented. xHTLs with a F/F_0 < 5 were considered as fluorogenic.

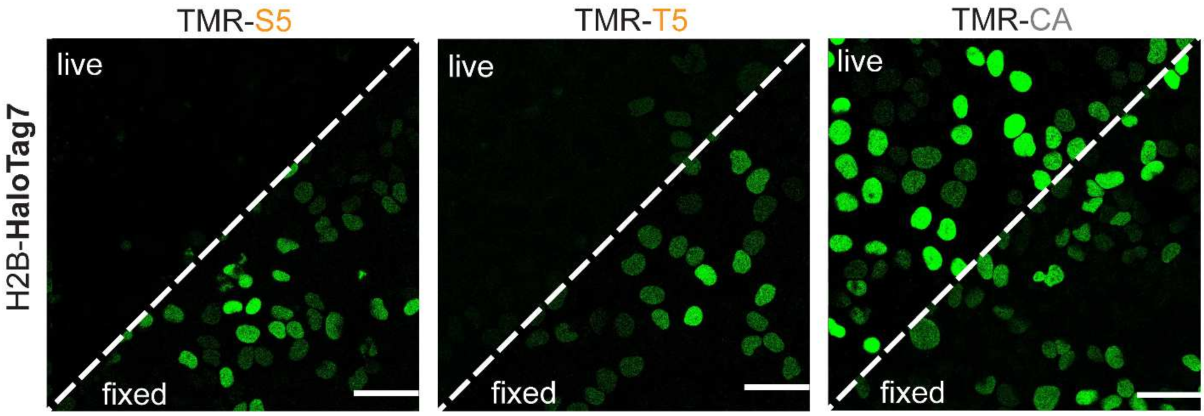

Figure S. 6 | Live-cell staining characterization of TMR-xHTLs.
Live- and fixed-cell confocal microscopy comparison of TMR-xHTLs probes. Histone2B-HaloTag7 expressing U2OS cells stained with 500 nM (x)HTLs. Sum projections. Scale bars: 10 µm. TMR-S5 and -T5 are overall not live-cell compatible in contrast to TMR-CA.

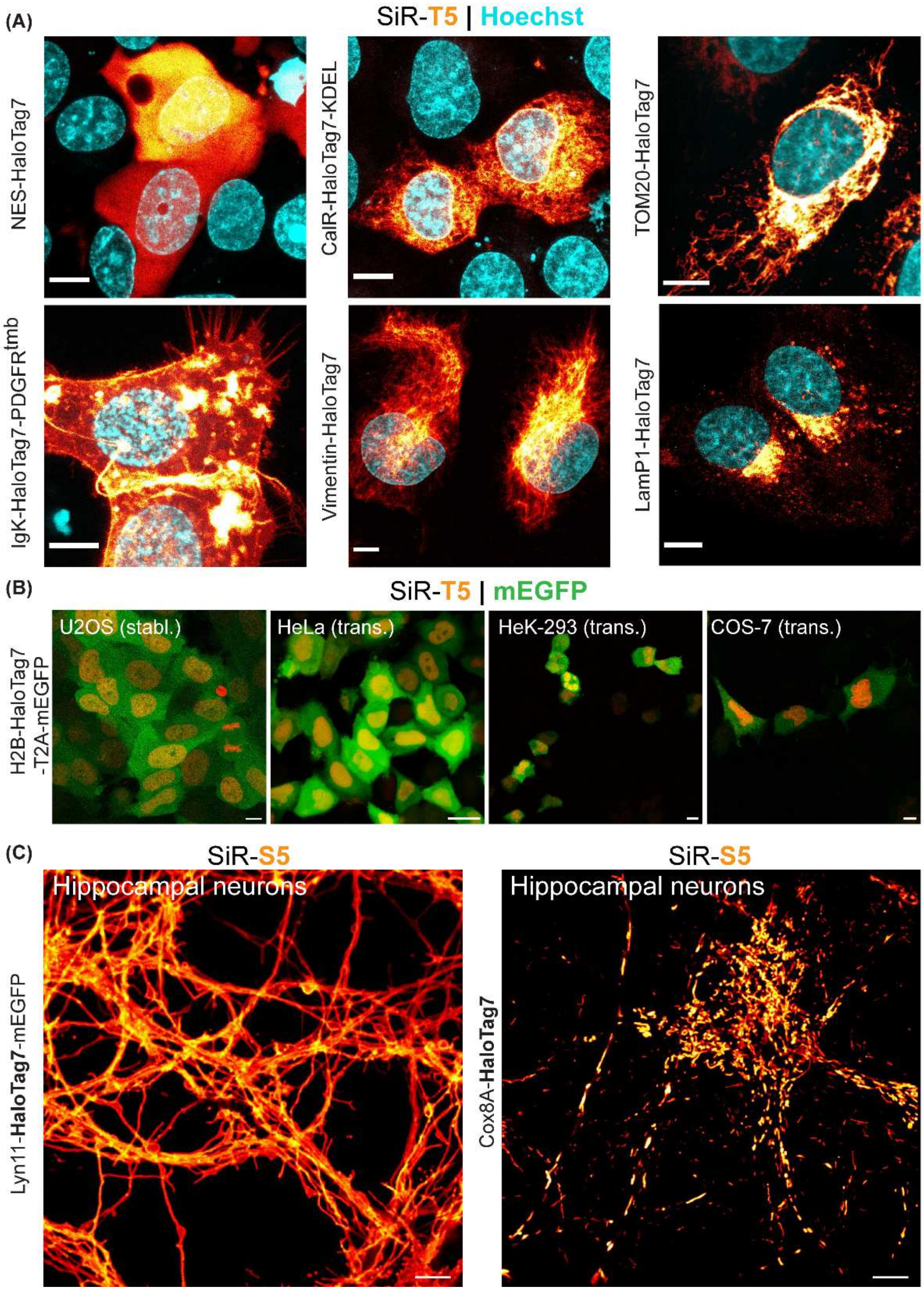

Figure S. 7 | Live-cell staining compatibility of SiR-xHTLs with different cellular targets and cell types.
(A) Images of sub-cellular structures in U2OS cells. HaloTag7-fusion protein overexpressed, stained with SiR-S5 (500 nM) and Hoechst (1 µg/mL) and live imaged by confocal microscopy (max. projection). Localizations (fusion protein): Cytoplasm (NES), outer-plasma membrane (IgK/PDGFRtmb), endoplasmic reticulum (CalR/KDEL), intermediate filaments (vimentin), mitochondria surface (TOM20) and lysosomes (LamP1). Scale bars: 10 µm. 'Hot' LUT were applied.
(B) Live-cell confocal images of U2OS cells stable (stabl.) expressing H2B-HaloTag7-T2A-mEGP and compared to transient transfection (trans.) with the same plasmid of HeLa Kyoto, HEK-293 and COS-7 cells. Max. projections. Scale bars: 10 µm.
(C) Primary rat hippocampal neurons expressing Lyn11- (outer membrane) and Cox8A (mitochondrial matrix) -HaloTag7 fusions. Neurons were transduced with rAAV carrying the respective transgene at 5 DIV and imaged at 10 DIV. Scale bars: 10 µm. 'Hot' LUT were applied.

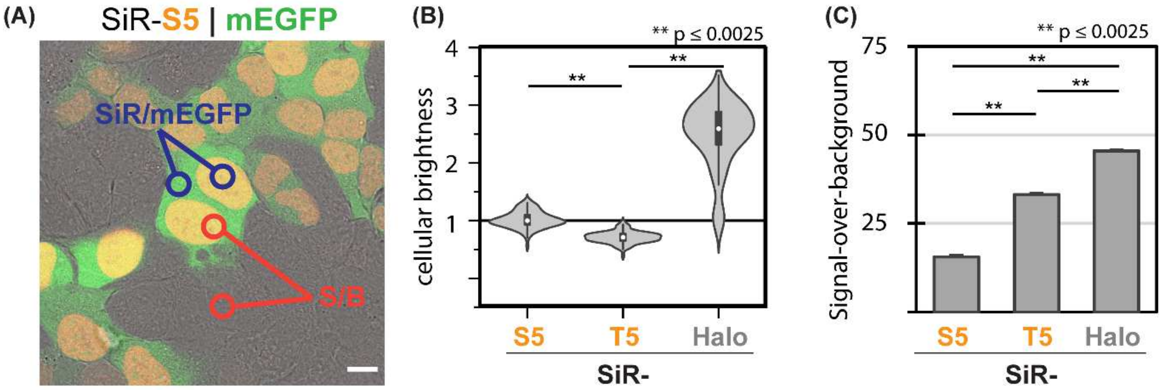

Figure S. 8 | xHTL live-cell staining characterization
(A) Confocal image illustrating the calculation of parameters employed to evaluate the SiR-(x)HTL staining quality. Live-cell confocal image of H2B-HaloTag7-T2A-mEGFP expressing U2OS cells stained with 500 nM SiR-S5. Overlay of SiR (red), EGFP (green) and bright-field (gray) channels, sum projections. The fluorescence intensity from single nuclei was normalized by the cytosolic mEGFP fluorescence intensity of the same cell (expression control) to assess the cellular brightness. The signal-over-background ratio (S/B) was evaluated by the ratio of the nuclear fluorescence intensity of cells expressing H2B-HaloTag7 or not. Scale bar: 10 µm. (B) Comparison of cellular brightness obtained for different SiR-(x)HTLs as explained before. n ≥ 150 cells, 4 images from at least two individual experiments. SiR-S5 shows superior cellular brightness (on HaloTag7) than SiR-T5 by 1.5-fold but the covalent staining remains generally brighter by a factor of 2 – 3 compared to any xHTL staining. Violin plot = light grey, whisker plot = dark grey, box = 25%–75% percentile and whiskers = 5%–95% percentile, circle = mean. Populations were normalized to the mean brightness of SiR-S5 (horizontal reference line). Significance was calculated using two-sided t-tests including the Welch correction, not significant (n.s.): p > 0.05 (*), significant: p ≤ 0.0025 (**). (C) Comparison of live cell S/B of SiR-(x)HTL stains under confocal imaging conditions (n ≥ 150 cells, 4 images from at least two individual experiments, mean values ± S.E.M.). SiR-T5 shows improved S/B by 2.4-fold over SiR-S5 but remains nevertheless 20 ± 1% lower than HaloTag7 covalent labeling.

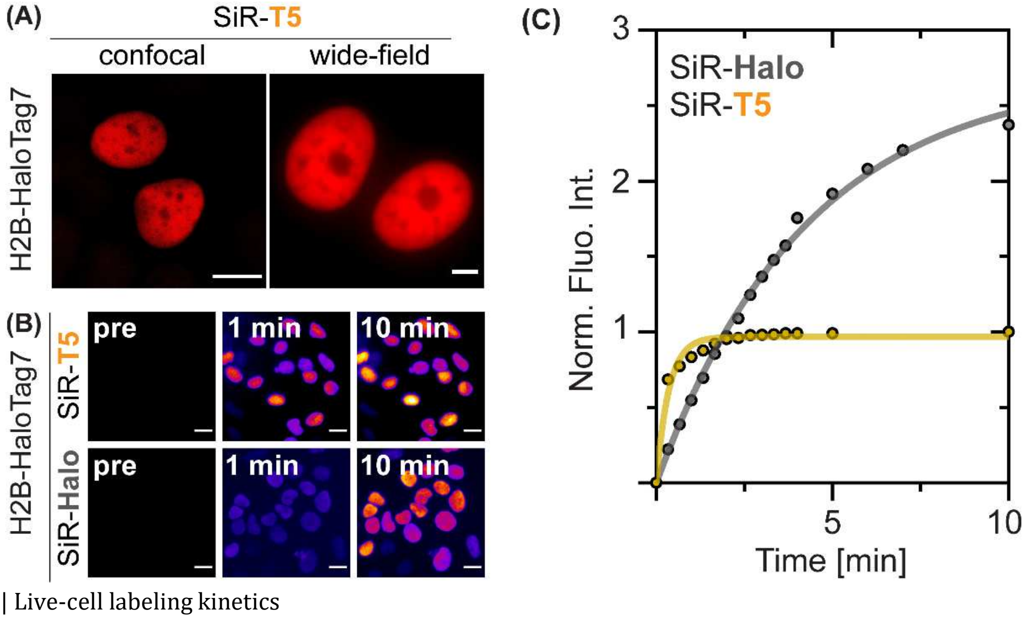

Figure S. 9 | Live-cell labeling kinetics
(A) SiR-T5 staining under confocal (500 nM) and wide-field (100 nM) microscopy conditions. Live U2OS cells expressing H2B-HaloTag7. Scale bars: 5 µm. SiR-T5 enables to image cells in wide-field microscopy despite the impossibility to conduct washing steps. (B) Live-cell staining kinetics comparing covalent and non-covalent SiR staining. U2OS express H2B-HaloTag7 stained with 25 nM SiR-(x)HTLs and imaged on a wide-field microscopy. Scale bars: 50 µm. (C) Signal from (B) (n ≥ 15 cells from one experiment) was normalized to maximum final signal obtained after 10 min for SiR-T5. Average data from n ≥ 15 cells (from one experiment) was fitted with a mono-exponential association function yielding the half-labeling times $\tau_{1/2}^{kin}$ (mean ± standard fitting error). SiR-T5: 0.2 ± 0.1 min, SiR-Halo: 2.9 ± 0.6 min.

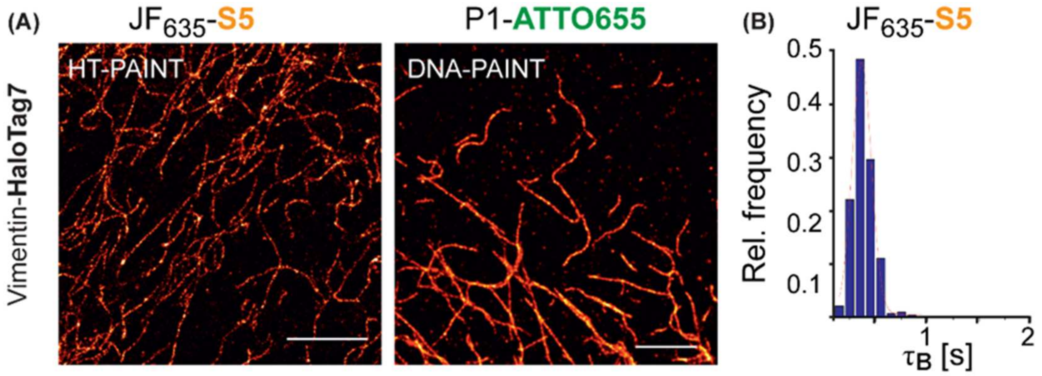

Figure S. 10 | Comparison of HaloTag-based PAINT and DNA-PAINT.

(A) Comparison of HaloTag-based PAINT and DNA-PAINT imaging. For HaloTag based PAINT, fixed U2OS cell endogenously expressing vimentin-HaloTag7[38] were stained with JF_{635}-S5 (1 nM). For DNA-PAINT, anti-vimentin immunostaining was used to label vimentin from the same cell-line with a DNA-oligonucleotide (docking strand). Complementary imager strand (P1-Atto655, 1 nM) was used for staining. Scale bars: 1 µm. Reconstructed super-resolution images with 31 nm (HaloTag-based PAINT) or 32 nm (DNA-PAINT) spatial resolution (decorrelation analysis). The experimental localization precision was calculated to be 11 nm (NeNA). Due to the smaller label size, HaloTag-fusions should in principle grant higher spatial resolution as compared to DNA-labeled antibodies. However, due to the vimentin heterogeneity this was not observable. The discontinuous appearance of the intermediate filaments revealed by JF_{635}-S5 might be explained by the heterozygote vimentin-HaloTag7 tagging.[38] Also, as previously reported, HaloTag7 shows lower labeling efficiency in fixed samples.[45] (B) Exemplary relative frequency distribution recorded for JF_{635}-S5. A Gaussian function was fitted to the data to determine the mean bright times (τ_B) in experiment performed as described in A. Analogously, the τ_B [ms] was obtained for additional xHTLs (mean ± standard deviation): SiR-S5 = 715 ± 15, JF_{635}-S5 = 365 ± 5, SiR-T5 = 1125 ± 15. The inverse to τ_B yields the kinetic unbinding constant k_{off} [s^{-1}]: JF_{635}-S5: 2.74, SiR-S5: 1.68, SiR-T5: 1.50.

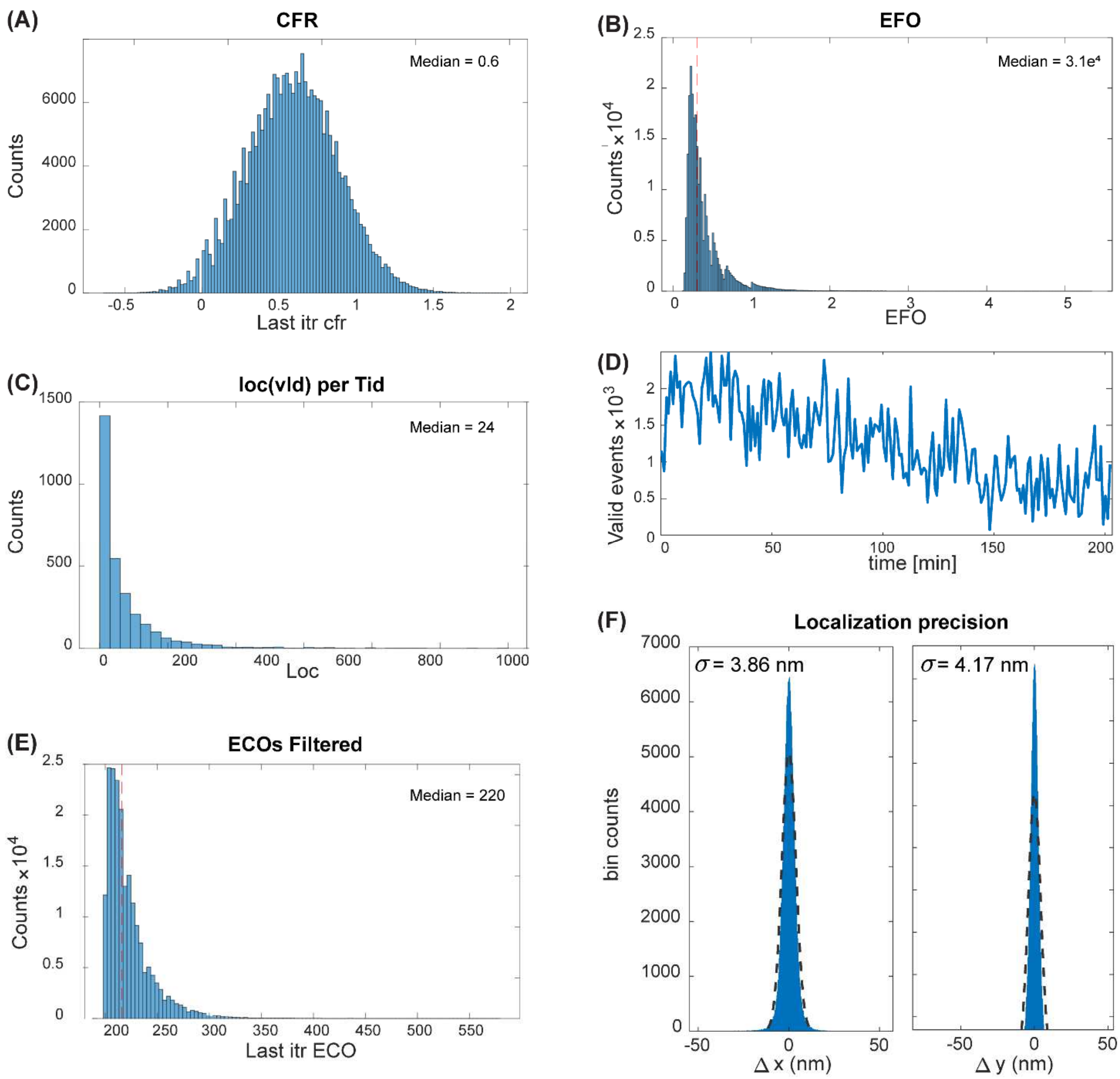

Figure S. 11 | Characterization of xHTLs performance in MINFLUX microscopy.

(A) – (D) Raw data used for filtering of MINFLUX measurements shown in Fig 5B. Center frequency ratio (CFR) from last iteration (itr), median CFR = 0.6 (A). Effective frequency at offset (EFO), median EFO = 30 kHz (B). Number of valid (val) localized events (loc) per trace-iD (Tid), median loc/TiD = 34 (C). Valid event count over time (D). Only events occurring from until 180 min were considered for MINFLUX data analysis. (E) Effective counts at offset (ECO) from after filtering. 220 photons (median) were used in the last iteration step for localization. (F) The localisation precision of MINFLUX measurements using SiR-T5 shown in **Fig. 5B** was calculated to be 3.86 nm (x) and 4.17 nm (y), respectively, after filtering the data. The discontinuous appearance of the intermediate filaments revealed by SiR-T5 (**Fig. 5B**) might be explained by the heterozygote vimentin-HaloTag7 tagging.[38]

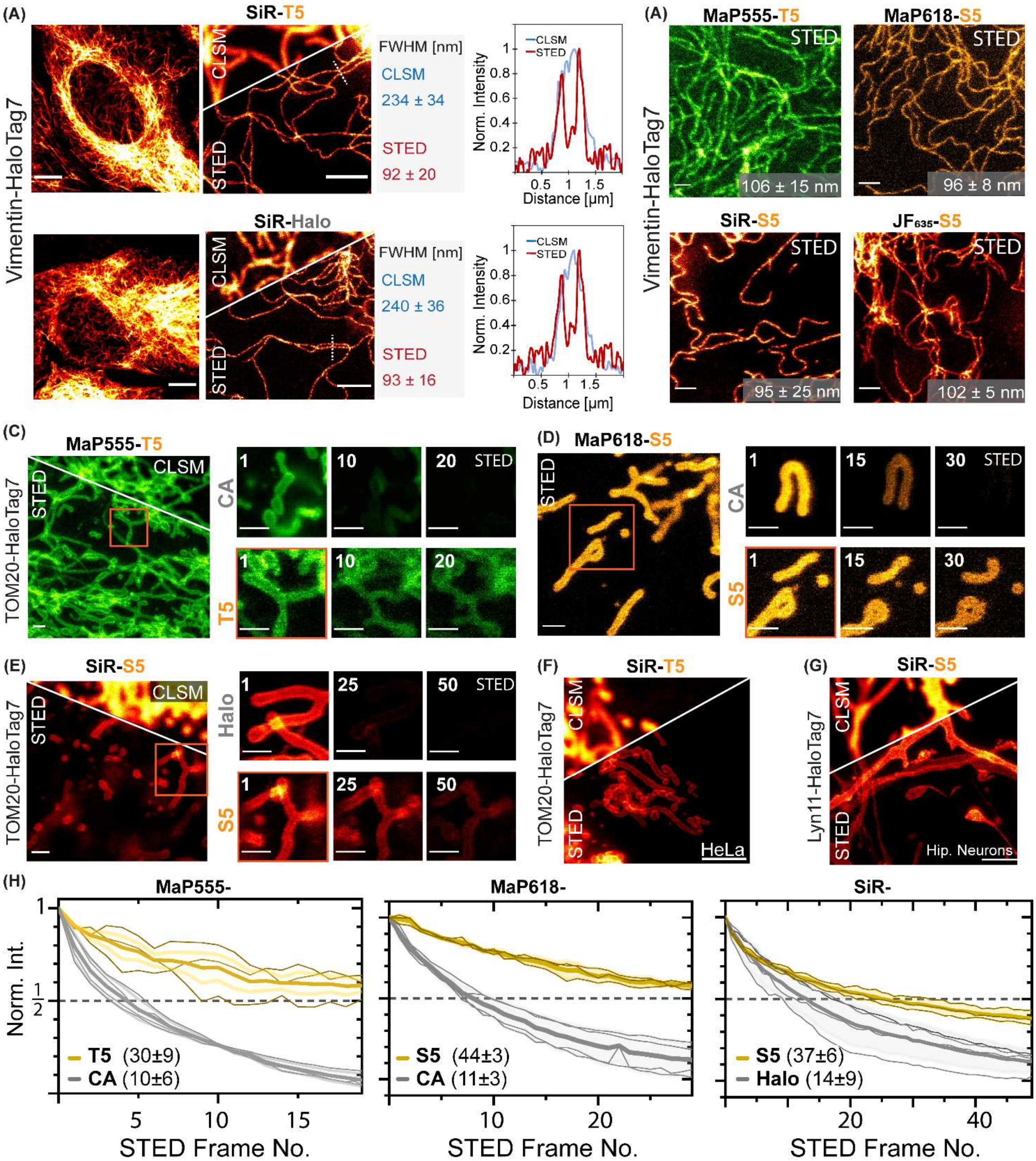

Figure S. 12 | STED photobleaching resistance of xHTLs compared to covalent HTLs.

A. Comparison of confocal laser scanning microscopy (CLSM) and STED images of intermediate filaments of live U2OS cells endogenously expressing vimentin-HaloTag7[38] and stained with SiR-T5 and SiR-Halo. Left side: confocal and STED images comparison. Middle: Comparison of the FWHM achieved under confocal and STED microscopy conditions (n > 15 filaments from ≥ 2 samples). Right side: representative intensity profile along two adjunct intermediate filaments. B. Live-cell STED-images using different xHTLs. Same cell line as in A. Scale bar: 1 µm. The mean filament diameter was characterized by calculating the full with at half maximum (FWHM) from fluorescence intensity profiles perpendicular to vimentin filaments under STED microscopy conditions (n > 15 filaments from ≥ 2 samples) as indicated in the bottom-right corner. C-E. Multi-frame STED imaging of U2OS mitochondria (TOM20-HaloTag7) stained with various xHTLs and HTLs. Left side: Overview

STED image (8x8 µm) of cells expressing TOM20-HaloTag7 stained with different xHTLs (500 nM) and used for signal quantification during multi-frame STED imaging. Scale bar: 1 µm. Middle: Exemplary zoom on dynamic mitochondria during multi-frame STED-imaging comparing exchangeable xHTL and covalent HTL staining. Frame numbers indicated in the top left corner. Scale: 1 µm. F. Comparison of CLSM and STED images of live HeLa Kyoto cells mitochondria (TOM20-HaloTag7) stained with SiR-T5 (500 nM). Scale bar: 1 µm. G. Comparison of CLSM and STED images of Lyn11-HaloTag7 in cultured rat hippocampal neurons transduced with rAAV carrying the respective transgene at day 5, stained with SiR-T5 (500 nM) and imaged at day 10 *in-vitro*. Scale bars: 1 µm. Scale bar: 1 µm. H. Bleaching curves for the data represented in C-E (thick lines: mean value and standard deviation, thin lines: individual experiments) and corresponding mean intensity half-life τ given in brackets (n ≥ 3, from at least 2 individual experiments).

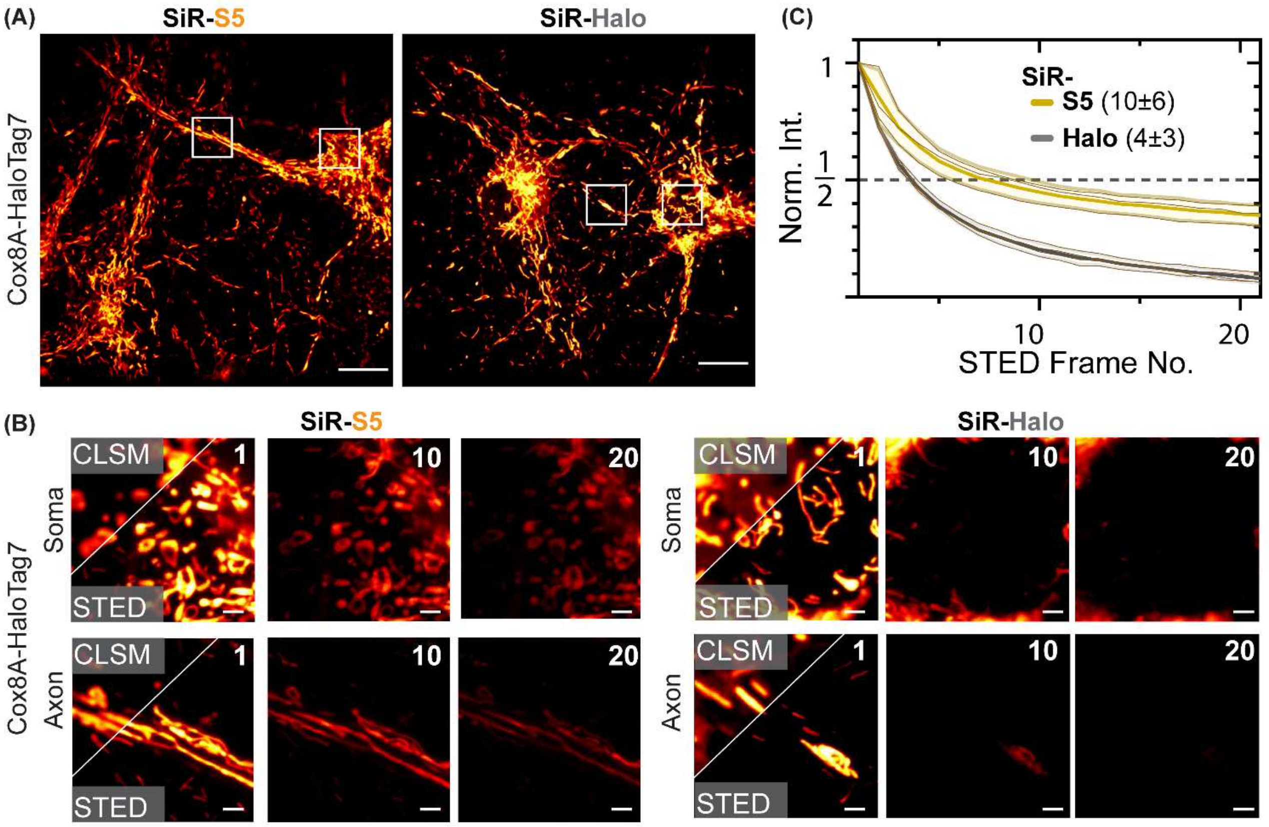

Figure S. 13 | Multi-frame STED imaging of xHTLs in rat hippocampal neurons

A. Confocal overview images of mitochondria (Cox8A-HaloTag7) of cultured rat hippocampal neurons and stained with SiR-S5 or -HTLs (500 nM). Scale bar: 10 µm. B. B. Multi-frame STED imaging (10x10 µm) of axonal or somatic mitochondria from images shown in A. Frame numbers indicated in the top left corner. Scale bar: 1 µm. C. Bleaching curves for the data represented in B (thick lines: mean value and standard deviation, thin lines: individual experiments) and corresponding mean intensity half-life τ given in brackets (n = 3, from 1 individual experiments).

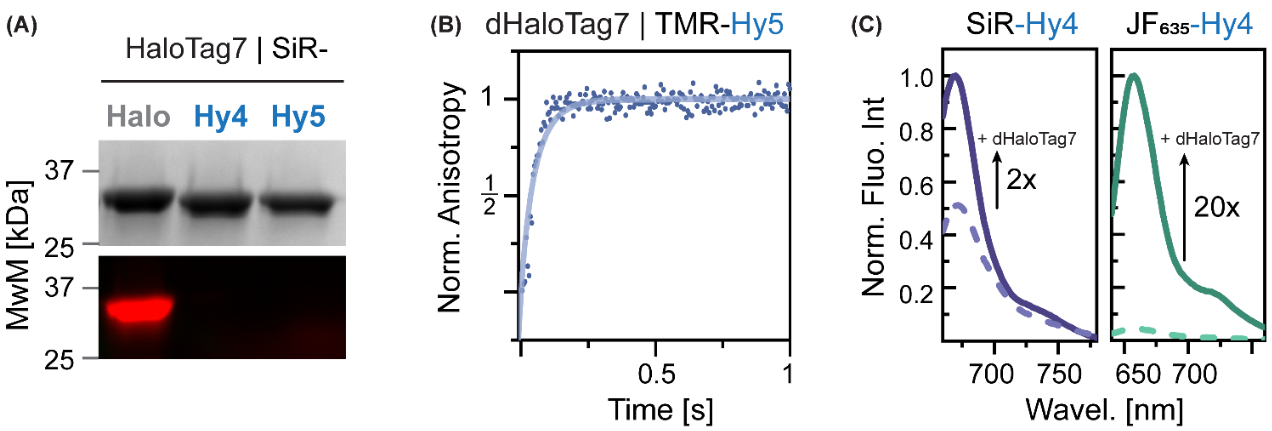

Figure S. 14 | Development of a second exchangeable xHTL/HaloTag7 protein pair.
A. HaloTag7 covalent labeling experiments. SDS-PAGE followed by in-gel fluorescence scan and Coomassie-staining. SiR derivatives of HTL/Hy4/Hy5 ligands were incubated with HaloTag7. xHTLs show non-covalent binding to HaloTag7 in contrast to HTL covalent labeling (SiR-Halo). MwM – Molecular weight marker. C. Binding kinetics of TMR-Hy5 to dHaloTag7 measured by stopped-flow fluorescence anisotropy experiments. Non-linear fitting from 10 kinetic traces yielded kinetic on-rate (k_{on}) C. Fluorescence emission spectra of free SiR/JF_{635}-Hy4 (dashed lines) or SiR/JF_{635}-Hy4 bound to dHaloTag7 (plain line). Arrows indicate fluorescence intensity increase (F/F_0) upon protein binding. SiR-Hy4 exhibits a low fluorescence increase upon protein binding which was restored by using the more fluorogenic dyes JF_{635}.

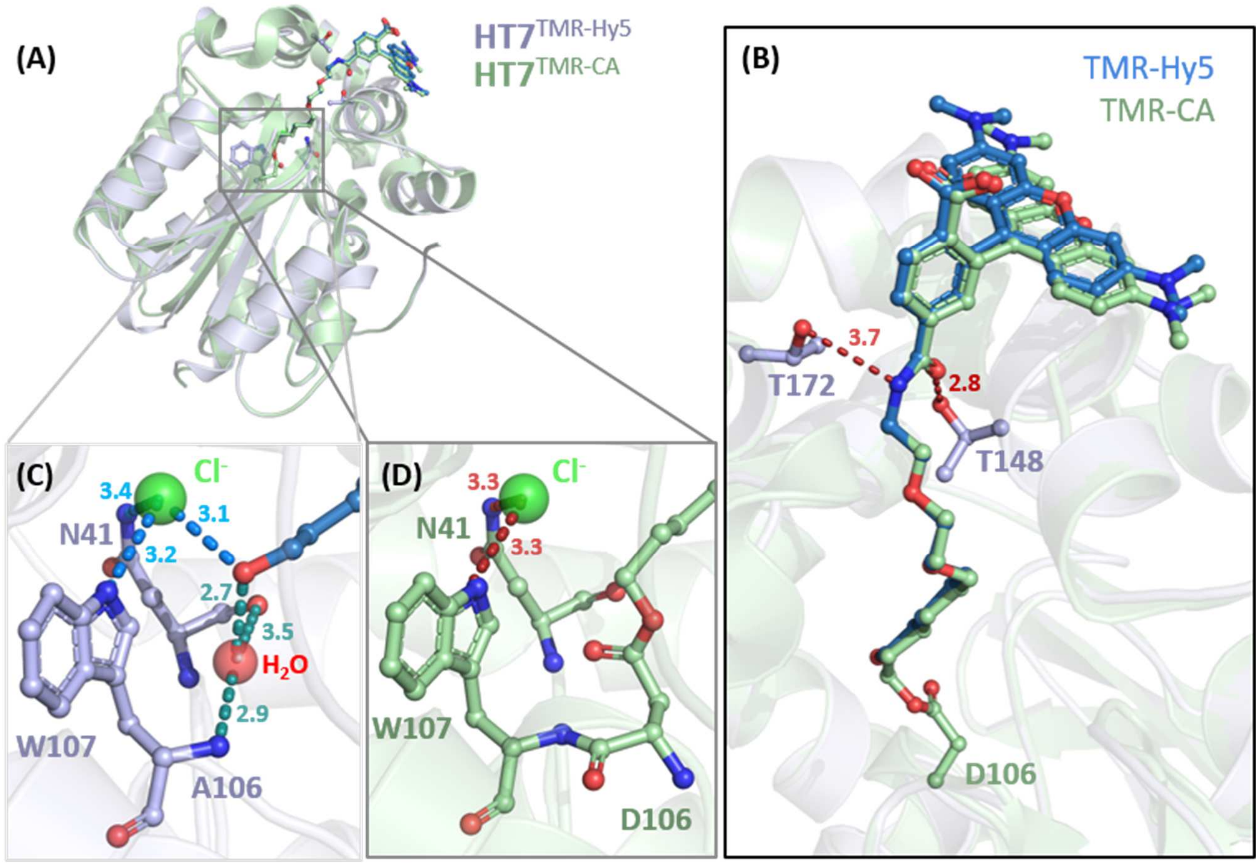

Figure S. 15 | Structural analysis of the TMR-Hy5/dHaloTag7 complex.

A. Structural comparison between the TMR-Hy5/dHaloTag7 complex (PDB ID: 7ZJZ, 1.5 Å resolution) and TMR-CA/HaloTag7 covalent complex (PDB ID: 6Y7A, 1.4 Å resolution)[1]. Tertiary structures are represented as cartoons. The ligands and relevant residues are represented as sticks. B. Magnification on the TMR-ligands binding sites. Distances in Å. The TMR moieties are located at the protein's surface while the alkane chains are buried in the protein hydrophobic tunnel as previously described.[1] C. Magnification on the Hy5 binding site. Polar interactions between Hy5 and dHaloTag7 residues: TMR-Hy5 interacts with a structural water and chloride-ion molecule, presented as spheres. The chloride ions forms hydrogen bonds with W107 and N41 site chain and the water molecule occupies the space freed by the D106A mutation and interacts with the W107 and N41 main chain. D. Magnification on the covalent bond between TMR-CA and the HaloTag7 protein. The TMR-labeled HaloTag7 features a chloride ion (green sphere) like found also in the TMR-Hy5/dHaloTag7 complex.

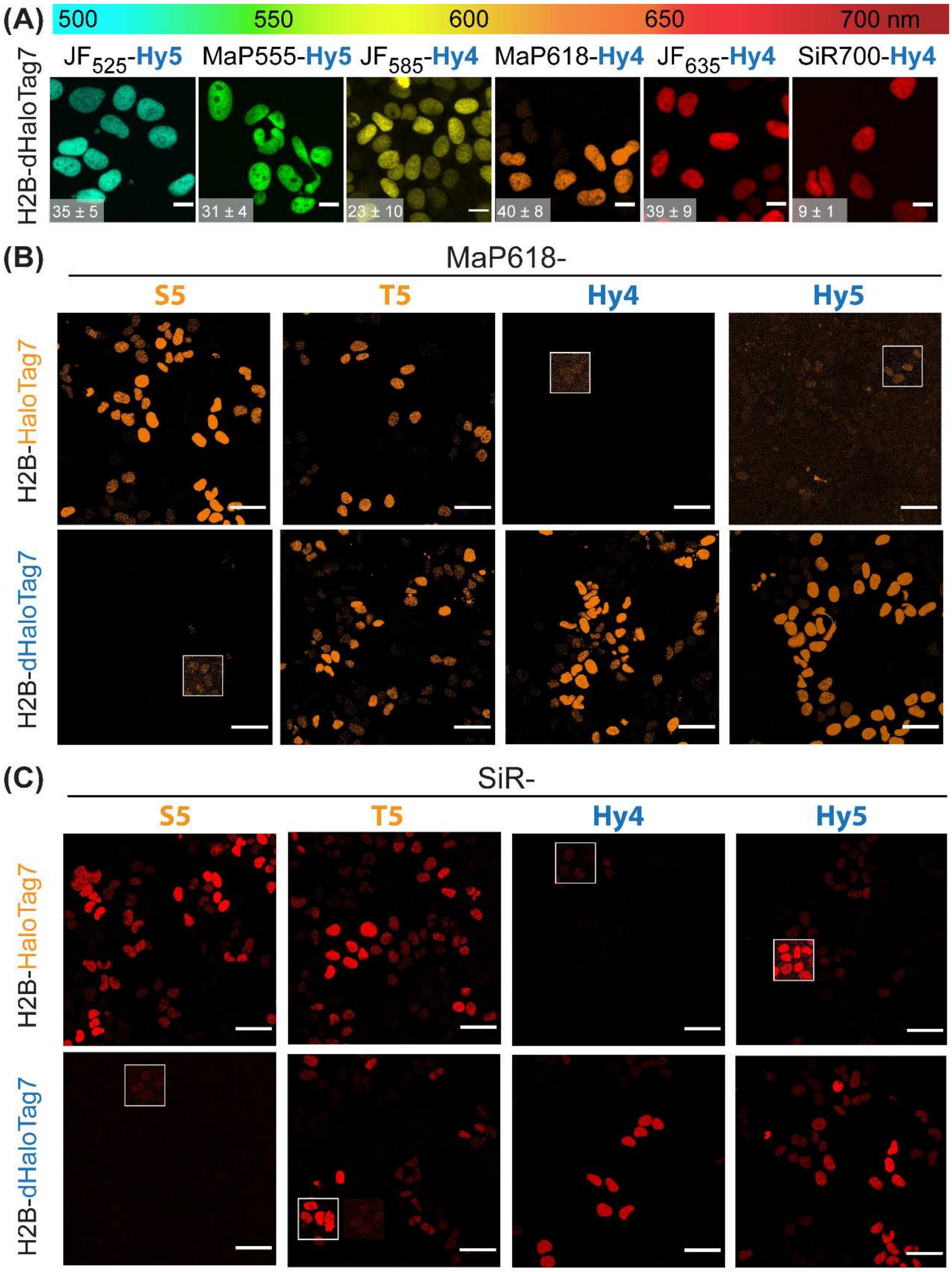

Figure S. 16 | (d)HaloTag7-specific xHTLs Hy4/Hy5 are combinable with S5/T5.
(A) Live-cell confocal images of different fluorescent xHTL probes covering the spectrum. H2B-dHaloTag7 expressing U2OS cells stained with 500 nM Hy5/Hy4 xHTLs probes and imaged via live-cell confocal microscopy. Sum projections. Scale: 10 µm. Signal-over-background ratios are given in bottom-left corner. (B) & (C) Staining specificity of MaP618- and SiR-xHTL. Live-cell confocal images of U2OS cells expressing H2B-HaloTag7 or -dHaloTag7 (500nM). White rectangles show increased contrast. Scales. 50 µm.

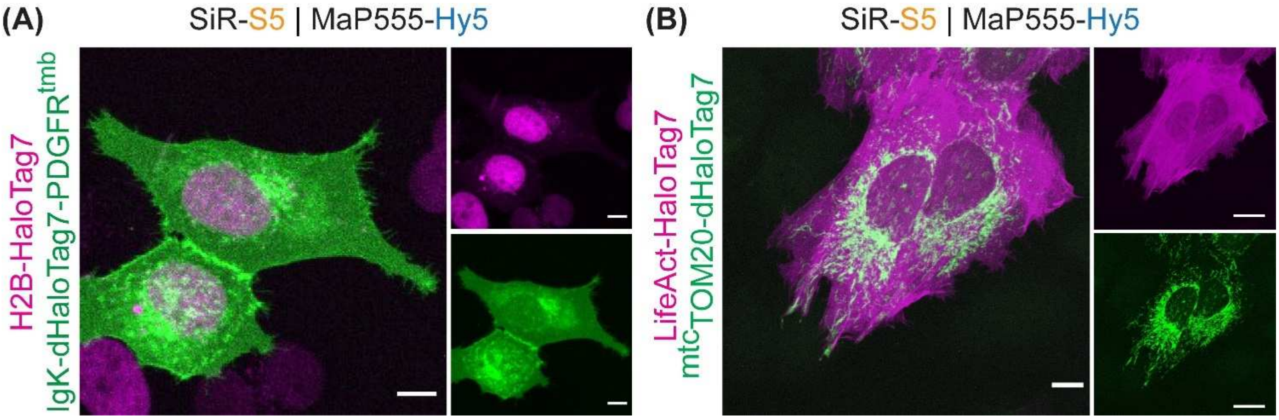

Figure S. 17 | xHTL/HaloTag pairs for dual-color imaging

Dual color images using xHTLs-(d)HaloTag7 pairs at different subcellular localization of live U2OS cells. 500 nM of xHTLs. Localizations: A. Nucleus (H2B) and plasma membrane (IgK/PDGFR), B. actin (LifeAct) and mitochondria (TOM20). Scale: 10 µm. Images are all max. projection.

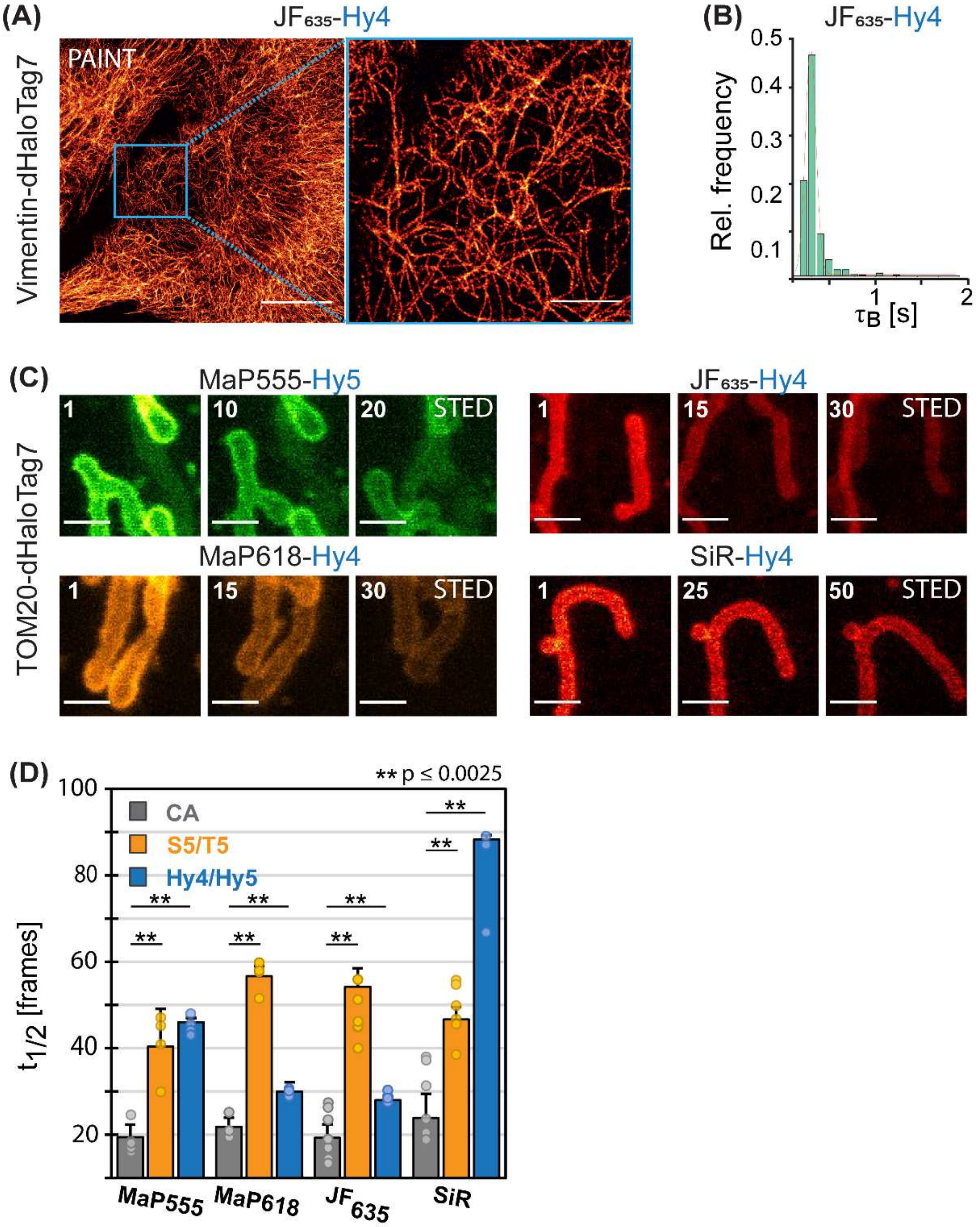

Figure S. 18 | Super-resolution fluorescence microscopy enabled by Hy4/5 and dHaloTag7.
(A) Reconstructed super-resolution image of fixed U2OS cells' intermediate filaments obtained by HaloTag-based PAINT microscopy using xHTLs and dHaloTag7 (36 nm resolution). Cells overexpressing Vimentin-dHaloTag7 were stained with JF_{635}-Hy4 (3 nM). Scale bars: 10 µm (overview) or 2 µm (magnified region). B. Exemplary relative frequency distribution recorded for JF_{635}-Hy4. A Gaussian function was fitted to the data to determine the mean bright times (τ_B) in experiment performed as described in A. Analogously, the τ_B [ms] parameter was obtained of additional xHTLs (mean ± S.D.): SiR-S5 = 715 ± 15, JF_{635}-S5 = 365 ± 5, SiR-T5 = 1125 ± 15, SiR-Hy4: 282 ± 4 and JF_{635}-Hy4: 233 ± 2. The inverse to τ_B yields the kinetic unbinding constant k_{off} [s^{-1}]: JF_{635}-Hy4: 4.35, SiR-Hy4: 3.4. C. Multi-frame STED images of U2OS mitochondria stained with xHTLs. Cells expressing TOM20-dHaloTag7 were stained with MaP555-Hy5 or MaP618-, JF_{635}-, SiR-Hy4 (500 nM). Frame numbers indicated in top-left corner. Scale bars: 10 µm. D. Comparison of STED bleaching experiments. Number of frames at which the intensity reaches half the initial one (half-life $\tau_{1/2}$) for various fluorophore-(x)HTLs. Normalized mean intensities were background corrected and fitted with a mono-exponential decay function. The half-life $\tau_{1/2}$ is represented. Individual data (dots), mean values (bars ± S.D., n≥3 experiments). Significance was calculated using two-sided t-test, not significant (n.s.): $p > 0.05$ (*), significant: $p \leq 0.0025$ (**).

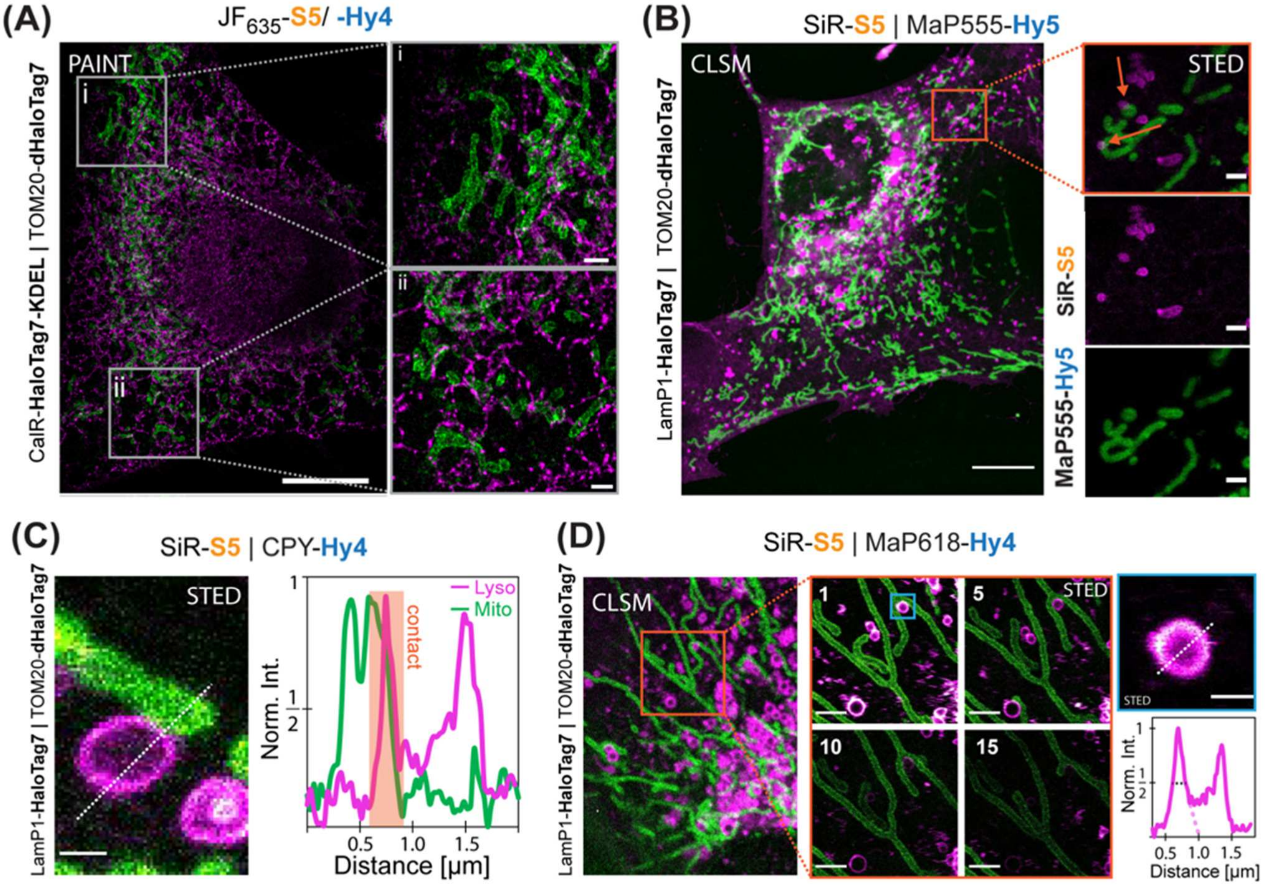

Figure S. 19 | Dual-color super-resolution microscopy using xHTLs.

A. Two-target super-resolution image of fixed U2OS cell's mitochondria and endoplasmic reticulum obtained by HaloTag-based PAINT microscopy using xHTLs. The cells were sequentially imaged with JF_{635}-S5 (5 nM) and JF_{635}-Hy4 (3 nM) targeting the endoplasmic reticulum (CalR-HaloTag7-KDEL) and mitochondria (TOM20-dHaloTag7), respectively. White boxes indicate magnified regions. Scale bars: 10 µm (overview) and 2 µm (magnified region). B. Dual-color, live-cell confocal and STED image of U2OS cells' lysosome and mitochondria labeled with xHTLs (500 nM). Cells express TOM20-HaloTag7 and LamP1-dHaloTag7 through a T2A translational fusion and were labeled by SiR-S5 (pink) and MaP555-Hy5 (green), respectively. Orange arrows indicate mitochondria-lysosome contact sites in the magnified region. Scale bars: 10 µm (overview) or 1 µm (magnified region). C. Dual-color STED image (magnification of image presented in Fig 2G) showing U2OS mitochondria-lysosome contacts (left panel). Cells were stained with SiR-S5 (pink) and CPY-Hy4 (green, 500 nM each). Scale bar: 0.5 µm. Right panel: fluorescence intensity profile along the white dashed line along the mitochondria-lysosome contact site from the STED image. It highlights the hollow lysosome and its thin contact with mitochondria at sub-diffraction resolution. D. Multi-frame STED imaging of U2OS cells expressing LamP1-HaloTag7 and TOM20-dHaloTag7 stained with SiR-S5 (pink) and MaP618-Hy4 (green, 500 nM each) respectively. Scale bar: 0.5 µm. The fluorescence profile along the white dashed line of the top right lysosome STED image highlights the hollow lysosome structure. The mean vesicular membrane diameter FWHM (black dashed line) under STED microscopy conditions was calculated to be 195 ± 85 (n = 15 vesicles).

Supplementary Tables

Table S. 1 | xHTL binding and spectral characterization

	Fluo-xHTL		Spectral prop.			Binding properties		Applications					
	xHTL	Dye	λ_{ext} [nm]	λ_{em} [nm]	Φ_{HT} ± S.D. %	K_D (CI 95%) [nM]	k_1 ± S.D. [10^6 M^{-1} s^{-1}]	F/F_0 ± S.D.	K_D^{HT7}/K_D^{dHT7} ± S.E.	Microscopy Conf.^{live}	STED^{live}	PAINT	MINFLUX
HT7 ligands	S5	TMR	555	578	61.3 ± 0.4	311 (275 - 351)	6.0 (5.8 – 6.1)	1.3 ± 0.1	57 ± 7	☐	☐	☐	☐
		CPY	616	636	72.2 ± 0.5	142 (115 - 176)	2.0 (1.9 – 2.0)	1.6 ± 0.3	N.D.	☑	☑	☐	☐
		MaP618	616	636	67.4 ± 0.1	N.D.	N.D.	29.9 ± 0.3	N.D.	☑	☑	☐	☐
		JF_{635}	635	660	59.6 ± 0.9	117 (84 - 162)	6.0 (5.5 – 6.6)	8.7 ± 2.0	N.D.	☑	☑	☑	☑
		SiR	646	672	51.3 ± 0.4	109 (91 - 130)	4.7 (4.4 – 4.9)	2.5 ± 0.7	56 ± 7	☑	☑	☑	☐
	T5	TMR	555	578	60.2 ± 0.1	166 (150 - 185)	5.6 (5.5 – 5.8)	1.4 ± 0.1	10 ± 2	☐	☐	☐	☐
		MaP555	558	578	45.3 ± 0.6	167 (63 - 70)	8.1 (7.8 – 8.5)	5.8 ± 1.4	N.D.	☑	☑	☐	☐
		CPY	616	636	68.8 ± 0.5	60 (48 - 75)	4.4 (4.3 – 4.5)	2.3 ± 0.3	N.D.	☑	☑	☐	☐
		SiR	646	672	50.2 ± 0.1	67 (48 - 93)	5.5 (5.1 – 5.8)	10 ± 3.9	4 ± 1	☑	☑	☑	☑
			λ_{ext}	λ_{em}	Φd_{HT} ± S.D.	K_D (C I95%)	k_1 ± S.D.	F/F_0 ± S.D.	K_D^{dHT7}/K_D^{HT7} ± S.E.	Conf.^{live}	STED^{live}	PAINT	MINFLUX
dHT7 ligands	Hy4	TMR	555	578	57.9 ± 0.4	1004 (836 - 1204)	14.7 (14.2 – 15.3)	1.1 ± 0.2	113 ± 21	☐	☐	☐	☐
		CPY	618	636	71.1 ± 0.5	219 (150 - 382)	9.4 (9.0 – 9.8)	1.5 ± 0.3	N.D.	☑	☑	☐	☐
		MaP618	618	636	68.3 ± 0.2	N.D.	N.D.	37 ± 6.5	N.D.	☑	☑	☐	☐
		JF_{635}	635	660	68.2 ± 0.3	272 (170 - 436)	19.0 (16.7 – 22.1)	19 ± 2.7	N.D.	☑	☑	☑	☐
		SiR	646	672	50.7 ± 0.4	385 (253 - 589)	13.3 (12.2 – 14.4)	1.3 ± 0.8	108 ± 14	☑	☑	☑	☐
	Hy5	TMR	555	578	60.9 ± 0.4	125 (101 - 154)	9.3 (8.9 – 9.7)	1.4 ± 0.2	65 ±16	☑	☐	☐	☐
		MaP555	558	578	53.8 ± 1.2	186 (123 - 189)	15.6 (14.9 – 16.3)	1.4 ± 0.2	N.D.	☑	☑	☐	☐
		SiR	646	672	61.2 ± 0.4	86 (74 - 100)	11.2 (10.7 – 11.7)	1.9 ± 0.2	51 ± 7	☑	☑	☑	☐

Φ_{HT} / Φ_{dHT} – Quantum Yield bound to excess (d)HaloTag7, CI 95% - 95% confidence interval, F/F_0 – Fluorescence intensity increase upon (d)HaloTag7 binding, S.D. – standard deviation, S.E. – standard error, N.D. – Not determined.

Table S. 2 | Data collection and refinement statistics for the crystal structure.

Data collection	HaloTag7 S5-TMR (7ZJ0)	HaloTag7 T5-TMR (7ZIY)	dHaloTag7 Hy5-TMR (7ZIZ)
Space group	P1	P1	$P2_12_12$
Unit-cell parameters			
a, b, c (Å)	44.29, 49.93, 78.72	44.24, 46.06, 79.06	77.83, 88.71, 44.20
α, β, γ (°)	71.23, 89.91, 67.81	94.41, 90.00, 109.51	90.00, 90.00, 90.00
Radiation source	PXII-X10SA, SLS	PXII-X10SA, SLS	PXII-X10SA, SLS
Wavelength (Å)	0.99988	0.99996	1.00008
Temperature (K)	100	100	100
Resolution range (Å)	50-1.50 (1.60-1.50)	50-1.70 (1.80-1.70)	50-1.50 (1.60-1.50)
No. of observed reflections	165811 (29286)	108087 (15170)	347669 (62060)
No. of unique reflections	85890 (14710)	59538 (8598)	49719 (8612)
Multiplicity	1.9 (2.0)	1.8 (1.8)	7.0 (7.2)
Completeness (%)	91.4 (88.6)	92.3 (84.7)	99.8 (99.6)
R_{merge} (%)	3.9 (43.2)	3.6 (23.4)	6.2 (34.4)
$\langle I/\sigma(I) \rangle$	11.8 (2.5)	12.3 (3.0)	18.7 (6.2)
$CC_{1/2}$ (%)#	99.7 (77.0)	99.8 (90.6)	99.8 (97.1)
**Refinement**			
Molecules per a.u.	2	2	1
No. of reflections	85886	59535	49714
No. of reflections in test set	4295	2977	2486
Resolution range (Å)	43.36-1.50	39.40-1.70	44.35-1.50
No. of non-hydrogen atoms			
Protein	4728	4714	2360
Ligand/ion	132	104	51
Water	417	271	233
Total	5277	5089	2644
R (%)	17.47	19.10	16.92
R_{free} (%)	20.52	22.43	19.48
RMS deviations from ideal			
bonds (Å)	0.013	0.012	0.011
angles (°)	1.264	1.226	1.188
B-factors (Å^2)			
Protein	19.26	20.32	16.69
Ligand/ion	22.25	21.03	18.41
Water	26.61	26.23	25.79
Average	19.92	20.65	17.52
Wilson B (Å^2)	16.69	19.22	15.44
Ramachandran statistics (%)			
favored regions	96.0	95.4	96.9
allowed regions	4.0	4.6	3.1
disallowed regions	0	0	0
Clashscore	1.78	1.70	1.06

Values in parentheses are for the highest resolution shell; #as implemented in XDS[19].

Table S. 3 | Fluorescence intensity increase of fluorogenic xHTL probes upon target binding.

Dye	S5	T5	Hy4	Hy5
JF_{525}	1.1 ± 1.6	5.2 ± 0.2	N.D.	1.4 ± 0.4
TMR	1.3 ± 0.1	1.4 ± 0.1	1.1 ± 0.2	1.4 ± 0.2
MaP555	1.5 ± 0.4	5.8 ± 1.4	N.D.	1.4 ± 0.2
JF_{585}	67.5 ± 6.7	85.8 ± 12.7	69.3± 4.9	N.D.
CPY	1.6 ± 0.3	2.3 ± 0.3	N.D.	N.D.
MaP618	29.9 ± 0.3	N.D.	37.1 ± 6.5	N.D.
JF_{635}	8.7 ± 2.0	N.D.	18.9 ± 2.7	N.D.
SiR	2.5 ± 0.7	10.2 ± 3.9	1.3 ± 0.8	1.9 ± 0.2
SiR700	2.8 ± 0.6	N.D.	2.9 ± 0.3	N.D.

S5/T5 binding to HaloTag7 and Hy4/Hy5 binding to dHaloTag7. N.D. – not determined.

Table S. 4 | Single-molecule binding kinetics and image resolution in HT-PAINT

Dye	Ligand	τ_b [ms] ± S.D.	k_{off} [s^{-1}]	resolution [nm]
SiR	S5	715 ± 15	1.7	34
JF_{635}	S5	365 ± 5	2.7	31
SiR	T5	1125 ± 15	1.4	32
SiR	Hy4	282 ± 4	3.4	37
JF_{635}	Hy4	233 ± 2	4.4	36
Atto655	P1 (DNA 9mer)	n.d.	n.d.	32

The spatial resolution was calculated by decorrelation analysis[35] and Fourier Ring Correlation (FRC)[36].

Protein sequences

>His-TEV-HaloTag7 (and variant)

MHHHHHHHHHHENLYFQGIGTGFPFDPHYVEVLGERMHYVDVGPRDGTPVLFLHGNPTSSYVWRNIIPHVAPTHRCIAPDLIGMG
KSDKPDLGYFFDDHVRFMDAFIEALGLEEVVLVIHXWGSALGFHWAKRNPERVKGIAFMEFIRPIPTWDEWPEFARETFQAFRTT
DVGRKLIIDQNVFIEGTLPMGVVRPLTEVEMDHYREPFLNPVDREPLWRFPNELPIAGEPANIVALVEEYMDWLHQSPVPKLLFWG
TPGVLIPPAEAARLAKSLPNCKAVDIGPGLNLLQEDNPDLIGSEIARWLSTLEI

Green: His-tag, Purple: TEV-cleavage site, Blue: HaloTag7, X = D for HaloTag7 & X = A for dHaloTag7.

NMR spectra

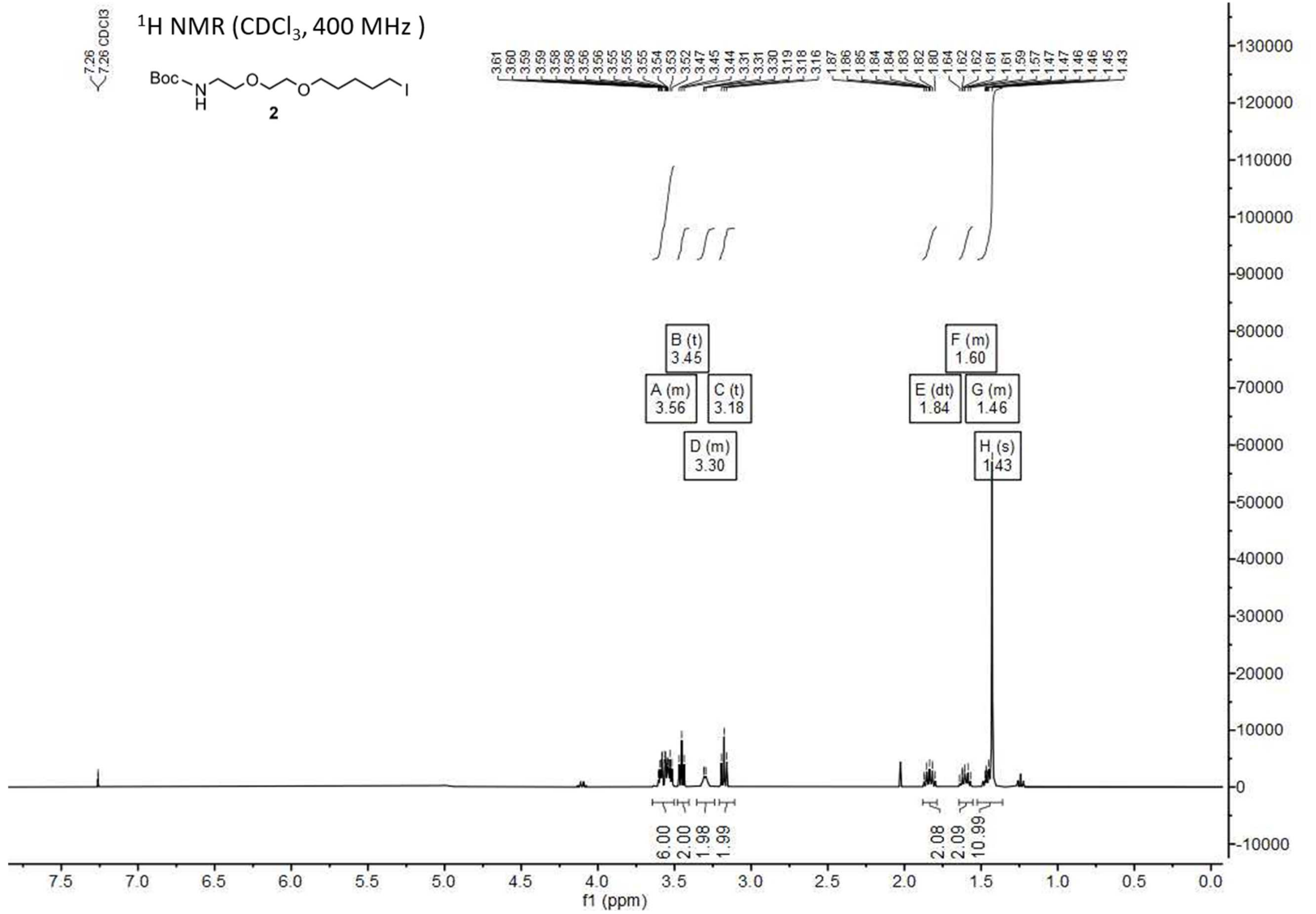

Spectrum 1. ^{1}H-NMR ($CDCl_3$) of Boc-PEG$_2$-C$_5$-I (**2**).

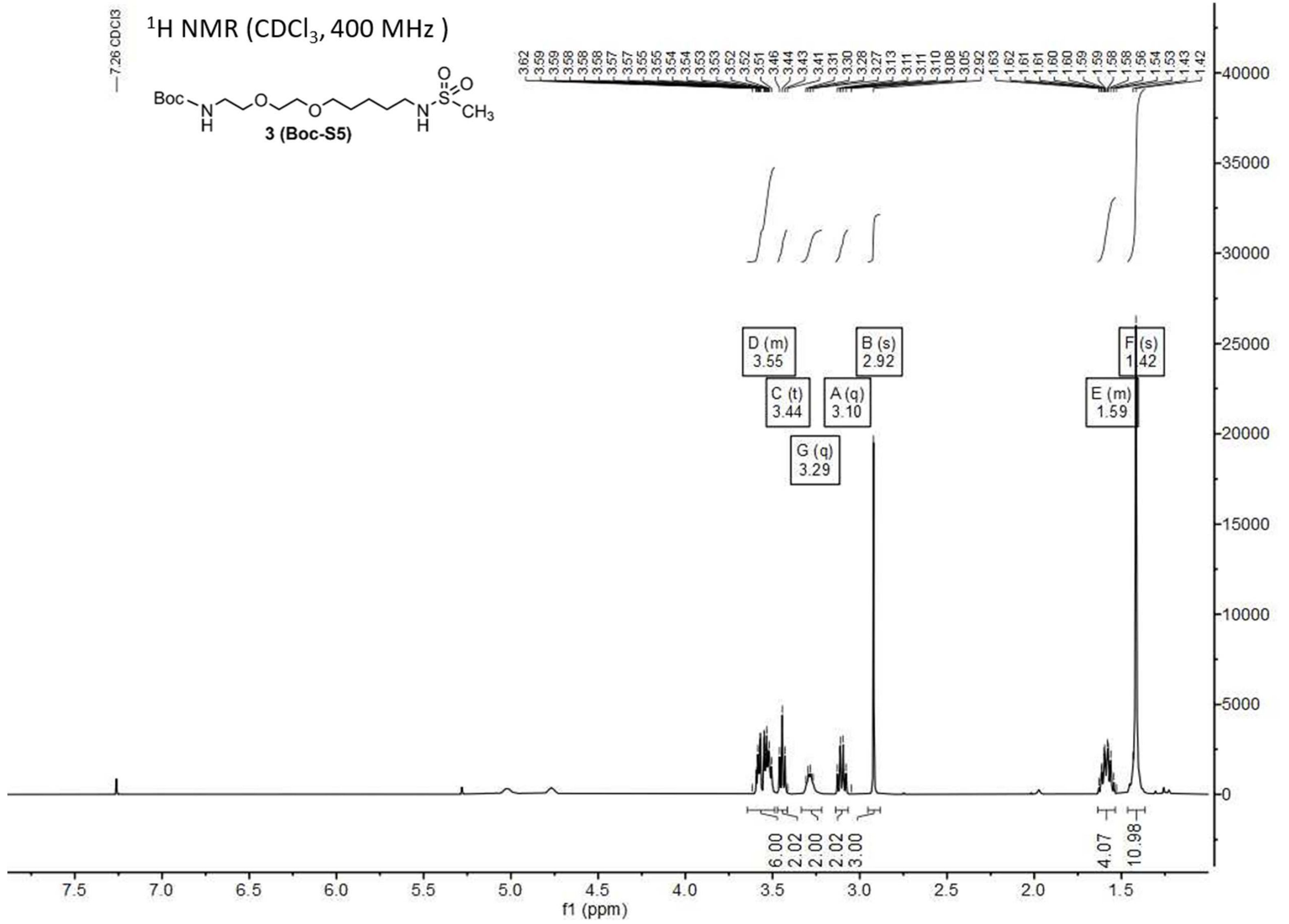

Spectrum 2. ^{1}H-NMR ($CDCl_3$) of Boc-S5 (**3**).

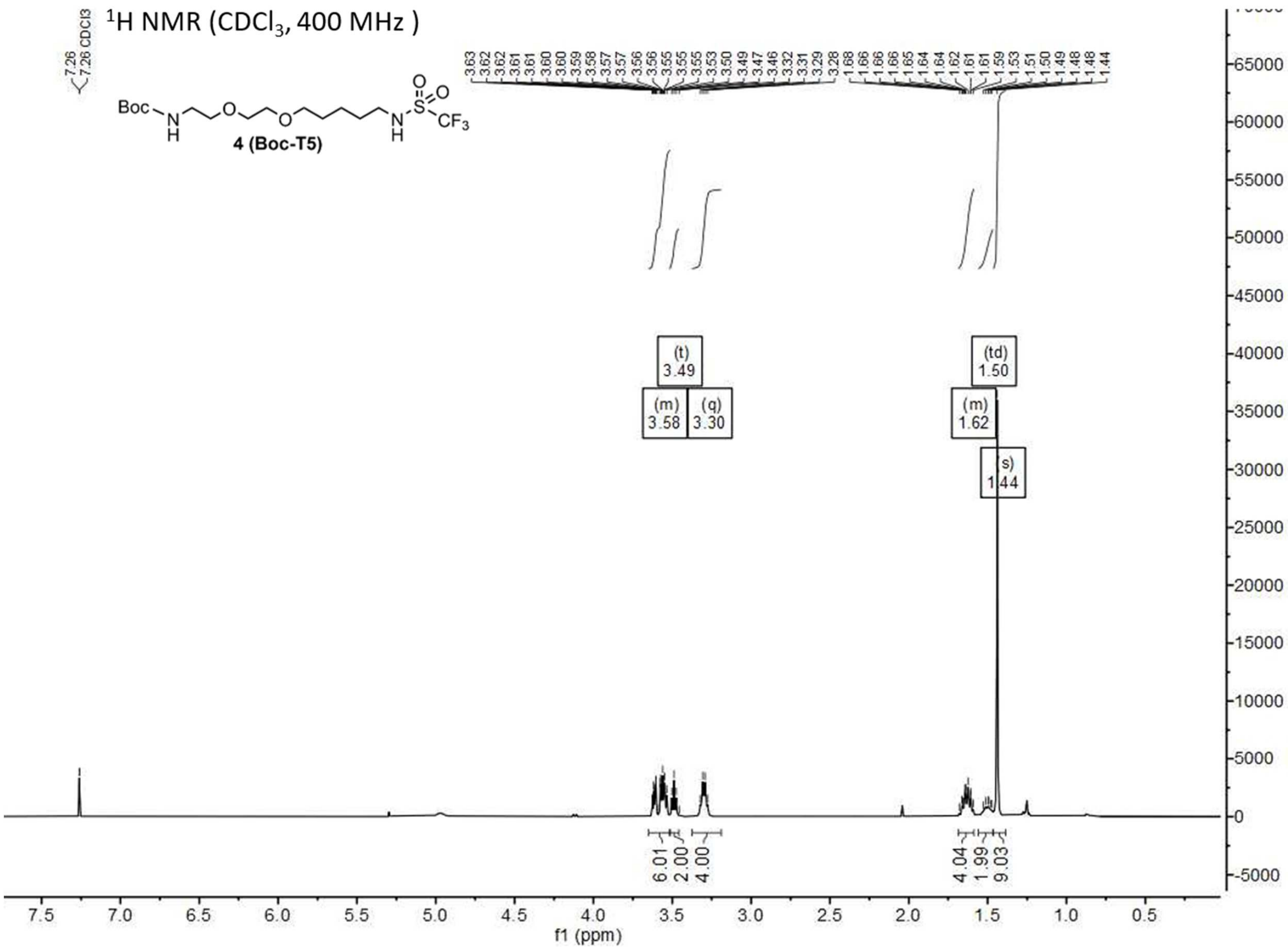

Spectrum 3. ^{1}H-NMR ($CDCl_3$) of Boc-T5 (**4**).

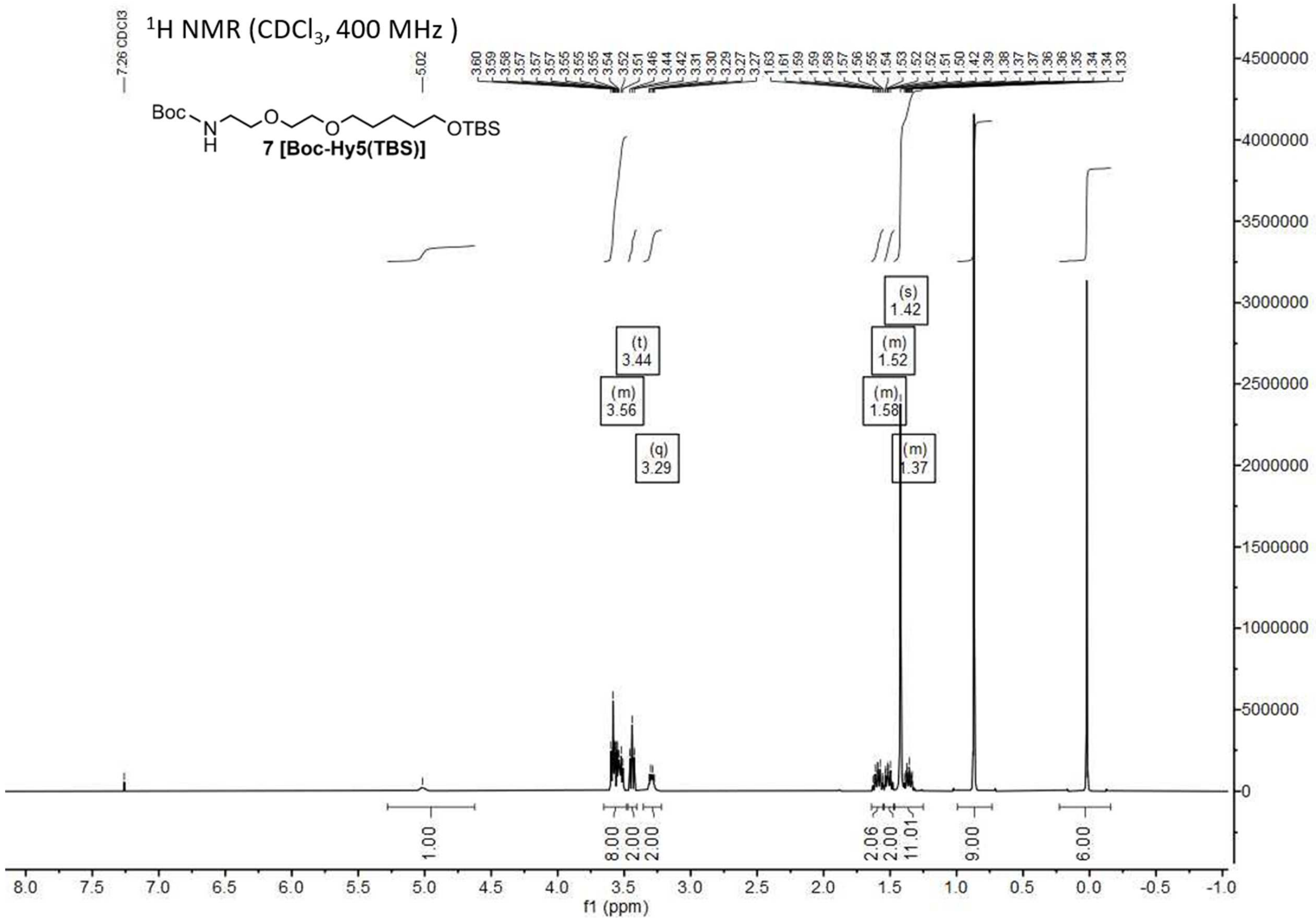

Spectrum 4. ^{1}H-NMR ($CDCl_3$) of Boc-Hy5-TBS (**7**).

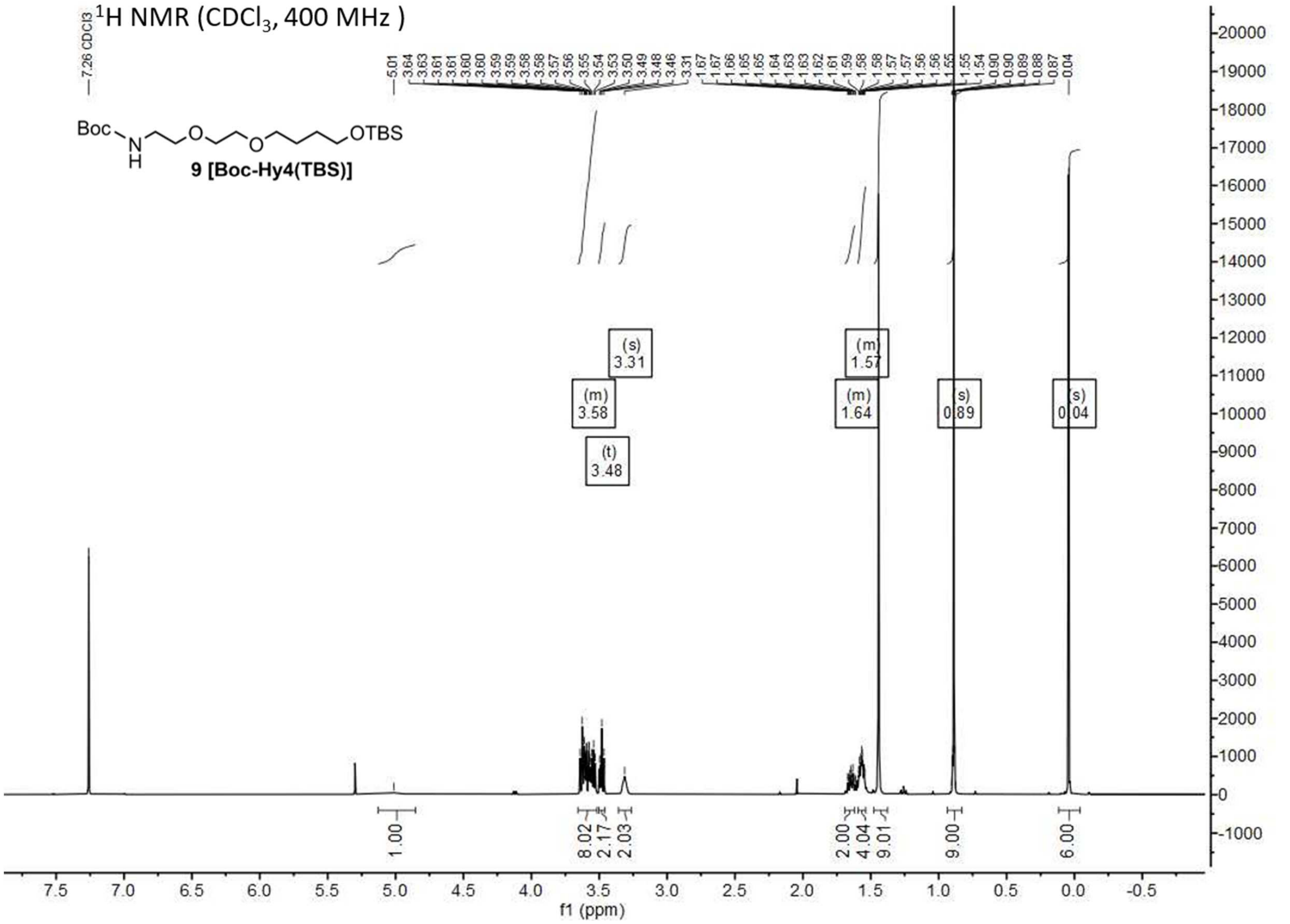

Spectrum 5. ¹H-NMR ($CDCl_3$) of Boc-Hy4-TBS (**9**).

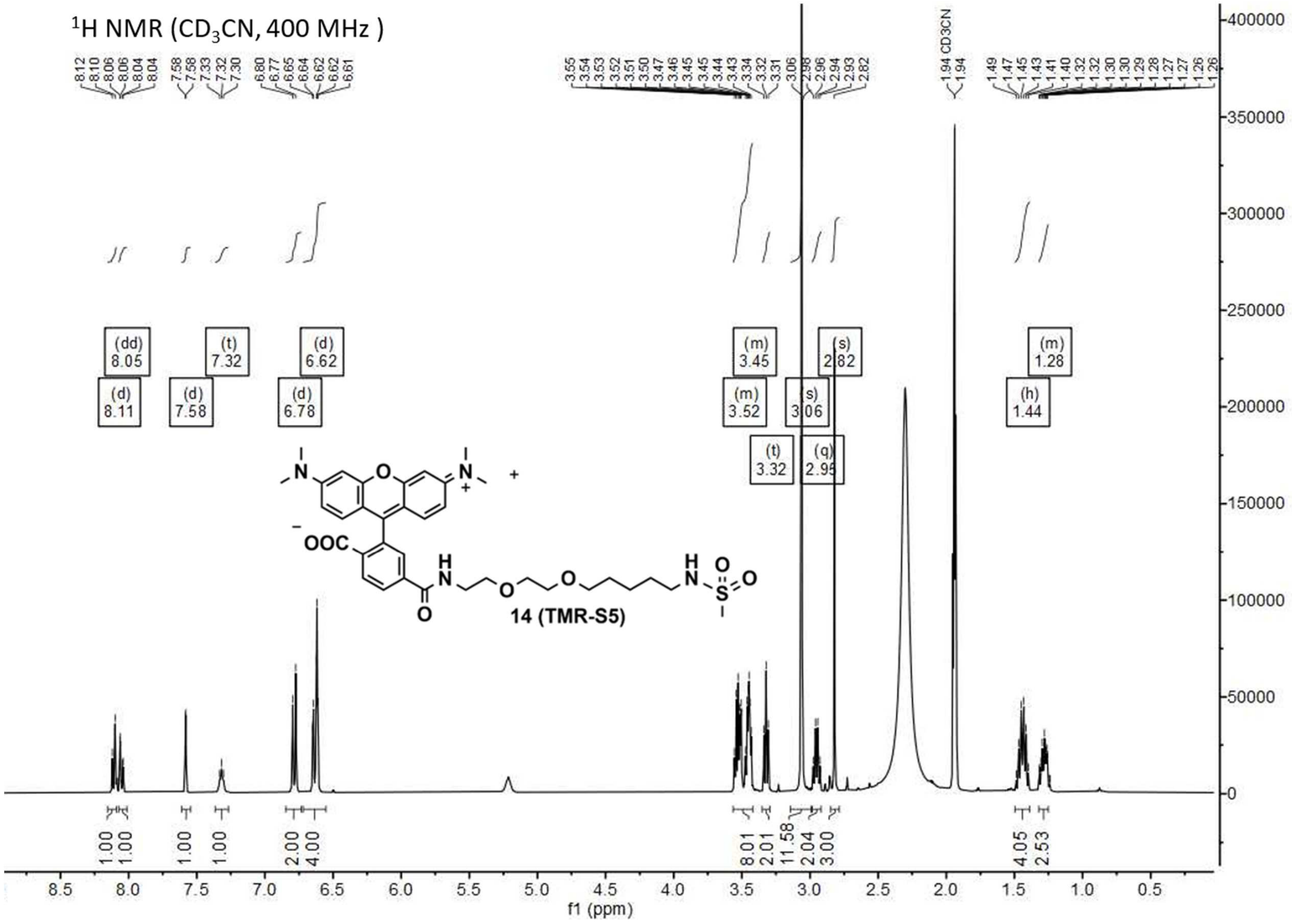

Spectrum 6. ^{1}H-NMR (CD_3CN) of TMR-S5 (**14**).

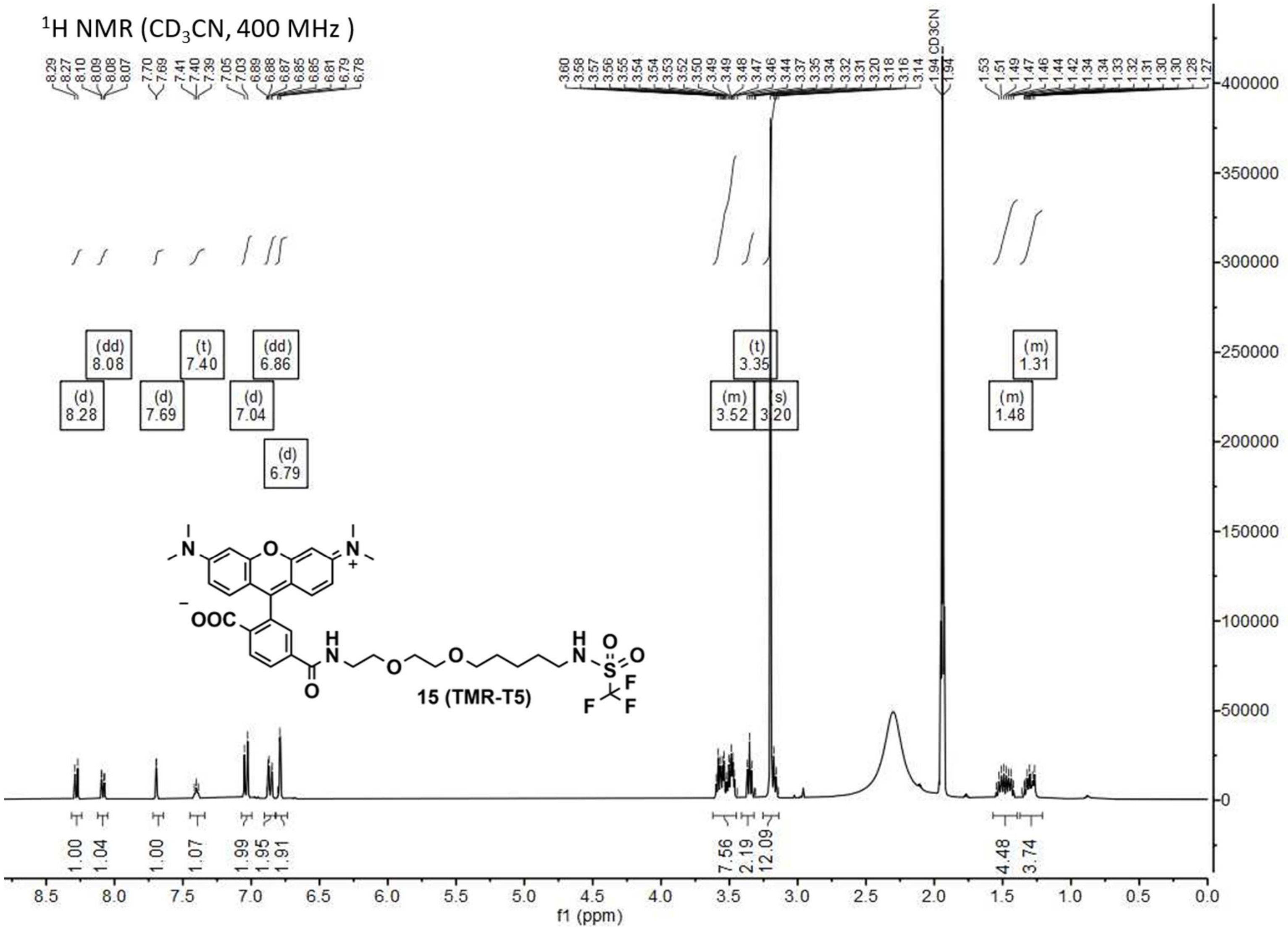

Spectrum 7. ^{1}H-NMR (CD_3CN) of TMR-T5 (**15**).

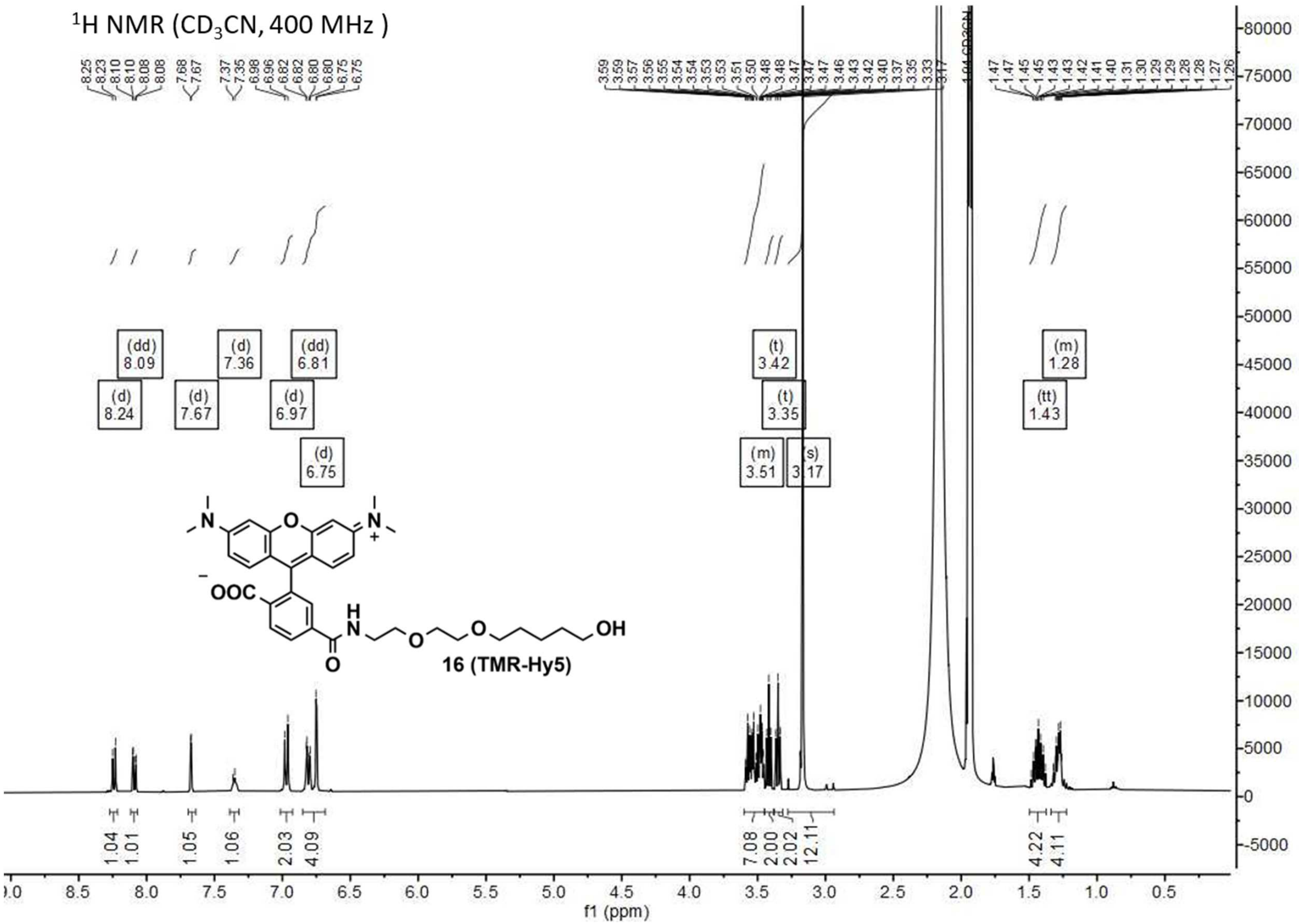

Spectrum 8. ^{1}H-NMR (CD_3CN) of TMR-Hy5 (**16**).

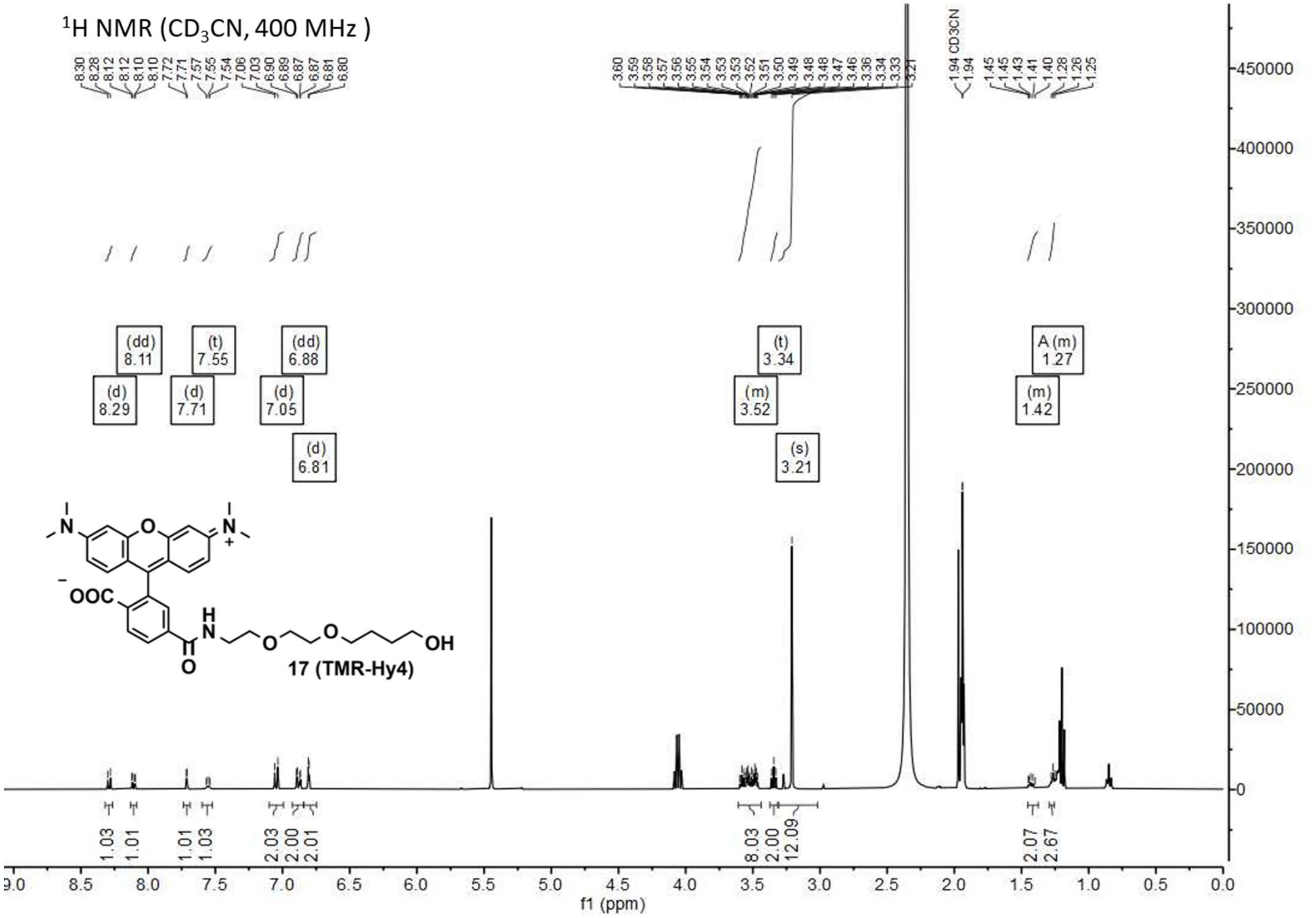

Spectrum 9. ^{1}H-NMR (CD_3CN) of TMR-Hy4 (**17**).
Residual DCM and EtOAc.

References

1. Wilhelm, J.; Kühn, S.; Tarnawski, M.; Gotthard, G.; Tünnermann, J.; Tänzer, T.; Karpenko, J.; Mertes, N.; Xue, L.; Uhrig, U.; Reinstein, J.; Hiblot, J.; Johnsson, K., Kinetic and Structural Characterization of the Self-Labeling Protein Tags HaloTag7, SNAP-tag, and CLIP-tag. *Biochemistry* **2021,** *60* (33), 2560–2575.
2. Friesner, R. A.; Banks, J. L.; Murphy, R. B.; Halgren, T. A.; Klicic, J. J.; Mainz, D. T.; Repasky, M. P.; Knoll, E. H.; Shelley, M.; Perry, J. K.; Shaw, D. E.; Francis, P.; Shenkin, P. S., Glide: a new approach for rapid, accurate docking and scoring. 1. Method and assessment of docking accuracy. *Journal of medicinal chemistry* **2004,** *47* (7), 1739–1749.
3. Shan, Y.; Kim, E. T.; Eastwood, M. P.; Dror, R. O.; Seeliger, M. A.; Shaw, D. E., How does a drug molecule find its target binding site? *JACS* **2011,** *133* (24), 9181–9183.
4. Schrödinger, L. *The PyMOL molecular graphics system v.2.1.1*, The PyMOL molecular graphics system v.2.1.1.
5. Grimm, J. B.; Muthusamy, A. K.; Liang, Y.; Brown, T. A.; Lemon, W. C.; Patel, R.; Lu, R.; Macklin, J. J.; Keller, P. J.; Ji, N.; Lavis, L. D., A general method to fine-tune fluorophores for live-cell and in vivo imaging. *Nat. Meth.* **2017,** *14* (10), 987–994.
6. Mudd, G.; Pi, I. P.; Fethers, N.; Dodd, P. G.; Barbeau, O. R.; Auer, M., A general synthetic route to isomerically pure functionalized rhodamine dyes. *Methods and applications in fluorescence* **2015,** *3* (4), 045002.
7. Wang, L.; Tran, M.; D'Este, E.; Roberti, J.; Koch, B.; Xue, L.; Johnsson, K., A general strategy to develop cell permeable and fluorogenic probes for multicolour nanoscopy. *Nat. Chem.* **2020,** *12* (2), 165–172.
8. Butkevich, A. N.; Mitronova, G. Y.; Sidenstein, S. C.; Klocke, J. L.; Kamin, D.; Meineke, D. N. H.; D'Este, E.; Kraemer, P.-T.; Danzl, J. G.; Belov, V. N.; Hell, S. W., Fluorescent Rhodamines and Fluorogenic Carbopyronines for Super-Resolution STED Microscopy in Living Cells. *Angew. Chem. Int. Ed.* **2016,** *55* (10), 3290–3294.
9. Lukinavičius, G.; Umezawa, K.; Olivier, N.; Honigmann, A.; Yang, G.; Plass, T.; Mueller, V.; Reymond, L.; Corrêa, I. R.; Luo, Z.-G.; Schultz, C.; Lemke, E. A.; Heppenstall, P.; Eggeling, C.; Manley, S.; Johnsson, K., A near-infrared fluorophore for live-cell super-resolution microscopy of cellular proteins. *Nat. Chem.* **2013,** *5* (2), 132–139.
10. Grimm, J. B.; English, B. P.; Chen, J.; Slaughter, J. P.; Zhang, Z.; Revyakin, A.; Patel, R.; Macklin, J. J.; Normanno, D.; Singer, R. H.; Lionnet, T.; Lavis, L. D., A general method to improve fluorophores for live-cell and single-molecule microscopy. *Nat. Meth.* **2015,** *12* (3), 244-250.
11. Lukinavičius, G.; Reymond, L.; Umezawa, K.; Sallin, O.; D'Este, E.; Göttfert, F.; Ta, H.; Hell, S. W.; Urano, Y.; Johnsson, K., Fluorogenic Probes for Multicolor Imaging in Living Cells. *JACS* **2016,** *138* (30), 9365–9368.
12. Liu, Y.; Miao, K.; Dunham, N. P.; Liu, H.; Fares, M.; Boal, A. K.; Li, X.; Zhang, X., The Cation-π Interaction Enables a Halo-Tag Fluorogenic Probe for Fast No-Wash Live Cell Imaging and Gel-Free Protein Quantification. *Biochemistry* **2017,** *56* (11), 1585–1595.
13. Cabrita, L. D.; Gilis, D.; Robertson, A. L.; Dehouck, Y.; Rooman, M.; Bottomley, S. P., Enhancing the stability and solubility of TEV protease using in silico design. *Protein science : a publication of the Protein Society* **2007,** *16* (11), 2360–2367.
14. Frei, M. S.; Hoess, P.; Lampe, M.; Nijmeijer, B.; Kueblbeck, M.; Ellenberg, J.; Wadepohl, H.; Ries, J.; Pitsch, S.; Reymond, L.; Johnsson, K., Photoactivation of silicon rhodamines via a light-induced protonation. *Nat. Commun.* **2019,** *10* (1), 4580.
15. Frei, M. S.; Tarnawski, M.; Roberti, M. J.; Koch, B.; Hiblot, J.; Johnsson, K., Engineered HaloTag variants for fluorescence lifetime multiplexing. *Nat. Meth.* **2021,** *19*, 65–70.
16. Kim, J. H.; Lee, S.-R.; Li, L.-H.; Park, H.-J.; Park, J.-H.; Lee, K. Y.; Kim, M.-K.; Shin, B. A.; Choi, S.-Y., High cleavage efficiency of a 2A peptide derived from porcine teschovirus-1 in human cell lines, zebrafish and mice. *PloS one* **2011,** *6* (4), e18556.
17. Liu, Z.; Chen, O.; Wall, J. B. J.; Zheng, M.; Zhou, Y.; Wang, L.; Vaseghi, H. R.; Qian, L.; Liu, J., Systematic comparison of 2A peptides for cloning multi-genes in a polycistronic vector. *Sci. Rep.* **2017,** *7* (1), 2193.
18. Gibson, D. G.; Young, L.; Chuang, R.-Y.; Venter, J. C.; Hutchison, C. A.; Smith, H. O., Enzymatic assembly of DNA molecules up to several hundred kilobases. *Nat. Meth.* **2009,** *6* (5), 343–345.
19. Kabsch, W., XDS. *Acta crystallographica. Section D, Biological crystallography* **2010,** *66* (Pt 2), 125–132.
20. McCoy, A. J.; Grosse-Kunstleve, R. W.; Adams, P. D.; Winn, M. D.; Storoni, L. C.; Read, R. J., Phaser crystallographic software. *Journal of applied crystallography* **2007,** *40* (Pt 4), 658–674.
21. GlobalPhasingLtd, Grade Web Server.
22. Emsley, P.; Lohkamp, B.; Scott, W. G.; Cowtan, K., Features and development of Coot. *Acta crystallographica. Section D, Biological crystallography* **2010,** *66* (Pt 4), 486–501.
23. Murshudov, G. N.; Skubák, P.; Lebedev, A. A.; Pannu, N. S.; Steiner, R. A.; Nicholls, R. A.; Winn, M. D.; Long, F.; Vagin, A. A., REFMAC5 for the refinement of macromolecular crystal structures. *Acta crystallographica. Section D, Biological crystallography* **2011,** *67* (Pt 4), 355–367.

24. Adams, P. D.; Afonine, P. V.; Bunkóczi, G.; Chen, V. B.; Davis, I. W.; Echols, N.; Headd, J. J.; Hung, L.-W.; Kapral, G. J.; Grosse-Kunstleve, R. W.; McCoy, A. J.; Moriarty, N. W.; Oeffner, R.; Read, R. J.; Richardson, D. C.; Richardson, J. S.; Terwilliger, T. C.; Zwart, P. H., PHENIX: a comprehensive Python-based system for macromolecular structure solution. *Acta crystallographica. Section D, Biological crystallography* **2010,** *66* (Pt 2), 213–221.
25. Chen, V. B.; Arendall, W. B.; Headd, J. J.; Keedy, D. A.; Immormino, R. M.; Kapral, G. J.; Murray, L. W.; Richardson, J. S.; Richardson, D. C., MolProbity: all-atom structure validation for macromolecular crystallography. *Acta crystallographica. Section D, Biological crystallography* **2010,** *66* (Pt 1), 12–21.
26. Wang, C.; Taki, M.; Sato, Y.; Tamura, Y.; Yaginuma, H.; Okada, Y.; Yamaguchi, S., A photostable fluorescent marker for the superresolution live imaging of the dynamic structure of the mitochondrial cristae. *Proceedings of the National Academy of Sciences of the United States of America* **2019,** *116* (32), 15817–15822.
27. Critchfield, F. E.; Gibson, J. A.; Hall, J. L., Dielectric Constant for the Dioxane—Water System from 20 to 35°. *JACS* **1953,** *75* (8), 1991–1992.
28. Straume, M.; Johnson, M. L., Monte Carlo Method for determining complete confidence probability distributions of estimated model parameters. In *Numerical Computer Methods*, Elsevier: 1992; Vol. 210, pp 117–129.
29. Malecki, M. J.; Sanchez-Irizarry, C.; Mitchell, J. L.; Histen, G.; Xu, M. L.; Aster, J. C.; Blacklow, S. C., Leukemia-associated mutations within the NOTCH1 heterodimerization domain fall into at least two distinct mechanistic classes. *Mol Cell Biol* **2006,** *26* (12), 4642-51.
30. Zolotukhin, S.; Potter M Fau - Zolotukhin, I.; Zolotukhin I Fau - Sakai, Y.; Sakai Y Fau - Loiler, S.; Loiler S Fau - Fraites, T. J., Jr.; Fraites Tj Jr Fau - Chiodo, V. A.; Chiodo Va Fau - Phillipsberg, T.; Phillipsberg T Fau - Muzyczka, N.; Muzyczka N Fau - Hauswirth, W. W.; Hauswirth Ww Fau - Flotte, T. R.; Flotte Tr Fau - Byrne, B. J.; Byrne Bj Fau - Snyder, R. O.; Snyder, R. O., Production and purification of serotype 1, 2, and 5 recombinant adeno-associated viral vectors. **2002,** (1046-2023 (Print)).
31. Schindelin, J.; Arganda-Carreras, I.; Frise, E.; Kaynig, V.; Longair, M.; Pietzsch, T.; Preibisch, S.; Rueden, C.; Saalfeld, S.; Schmid, B.; Tinevez, J.-Y.; White, D. J.; Hartenstein, V.; Eliceiri, K.; Tomancak, P.; Cardona, A., Fiji: an open-source platform for biological-image analysis. *Nat. Meth.* **2012,** *9* (7), 676–682.
32. Agasti, S. S.; Wang, Y.; Schueder, F.; Sukumar, A.; Jungmann, R.; Yin, P., DNA-barcoded labeling probes for highly multiplexed Exchange-PAINT imaging. *Chemical science* **2017,** *8* (4), 3080–3091.
33. Edelstein, A. D.; Tsuchida, M. A.; Amodaj, N.; Pinkard, H.; Vale, R. D.; Stuurman, N., Advanced methods of microscope control using μManager software. *Journal of biological methods* **2014,** *1* (2).
34. Schnitzbauer, J.; Strauss, M. T.; Schlichthaerle, T.; Schueder, F.; Jungmann, R., Super-resolution microscopy with DNA-PAINT. *Nat. Protoc.* **2017,** *12* (6), 1198–1228.
35. Descloux, A.; Grußmayer, K. S.; Radenovic, A., Parameter-free image resolution estimation based on decorrelation analysis. *Nat. Meth.* **2019,** *16* (9), 918-924.
36. Nieuwenhuizen, R. P. J.; Lidke, K. A.; Bates, M.; Puig, D. L.; Grünwald, D.; Stallinga, S.; Rieger, B., Measuring image resolution in optical nanoscopy. *Nat. Meth.* **2013,** *10* (6), 557-562.
37. *Origin(Pro) v.2018b*, OriginLab Corporation: 2018.
38. Ratz, M.; Testa, I.; Hell, S. W.; Jakobs, S., CRISPR/Cas9-mediated endogenous protein tagging for RESOLFT super-resolution microscopy of living human cells. *Sci. Rep.* **2015,** *5*, 9592.
39. Schmidt, R.; Weihs, T.; Wurm, C. A.; Jansen, I.; Rehman, J.; Sahl, S. J.; Hell, S. W., MINFLUX nanometer-scale 3D imaging and microsecond-range tracking on a common fluorescence microscope. *Nat. Commun.* **2020,** *12*, 1478.
40. Pape, J. K.; Stephan, T.; Balzarotti, F.; Hell, S. W., Multicolor 3D MINFLUX nanoscopy of mitochondrial MICOS proteins. *PNAS* **2020,** *117*.
41. Butkevich, A. N.; Ta, H.; Ratz, M.; Stoldt, S.; Jakobs, S.; Belov, V. N.; Hell, S. W., Two-Color 810 nm STED Nanoscopy of Living Cells with Endogenous SNAP-Tagged Fusion Proteins. *ACS Chem Biol* **2018,** *13* (2), 475–480.
42. Simplício, A. L.; Clancy, J. M.; Gilmer, J. F., Prodrugs for amines. *Molecules (Basel, Switzerland)* **2008,** *13* (3), 519–547.
43. Zhang, Z.; Shen, W.; Ling, J.; Yan, Y.; Hu, J.; Chen, Y., The fluorination effect of fluoroamphiphiles in cytosolic protein delivery. *Nature Communications* **2018,** *9*, 1377.
44. Deo, C.; Abdelfattah, A. S.; Bhargava, H. K.; Berro, A. J.; Falco, N.; Farrants, H.; Moeyaert, B.; Chupanova, M.; Lavis, L. D.; Schreiter, E. R., The HaloTag as a general scaffold for far-red tunable chemigenetic indicators. *Nature chemical biology* **2021,** *17* (6), 718–723.
45. Thevathasan, J. V.; Kahnwald, M.; Cieśliński, K.; Hoess, P.; Peneti, S. K.; Reitberger, M.; Heid, D.; Kasuba, K. C.; Hoerner, S. J.; Li, Y.; Wu, Y.-L.; Mund, M.; Matti, U.; Pereira, P. M.; Henriques, R.; Nijmeijer, B.; Kueblbeck, M.; Sabinina, V. J.; Ellenberg, J.; Ries, J., Nuclear pores as versatile reference standards for quantitative superresolution microscopy. *Nat. Meth.* **2019,** *16* (10), 1045-1053.
